# Synthesis and Cytotoxic Activity of Lepidilines A–D: Comparison with Some 4,5-Diphenyl Analogues and Related Imidazole-2-thiones

**DOI:** 10.1021/acs.jnatprod.1c00797

**Published:** 2021-11-22

**Authors:** Grzegorz Mlostoń, Mateusz Kowalczyk, Małgorzata Celeda, Katarzyna Gach-Janczak, Anna Janecka, Marcin Jasiński

**Affiliations:** † Faculty of Chemistry, University of Lodz, 91403 Łódź, Poland; ‡ The Bio-Med-Chem Doctoral School of the University of Lodz and Lodz Institutes of the Polish Academy of Sciences, Faculty of Biology and Environmental Protection, University of Lodz, 90237 Łódź, Poland; § Department of Biomolecular Chemistry, Medical University of Lodz, 92215 Łódź, Poland

## Abstract

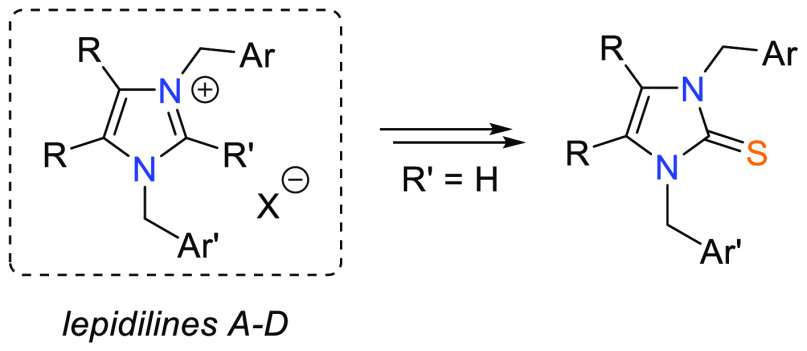

A straightforward access to 2-unsubstituted imidazole *N*-oxides with subsequent deoxygenation by treatment with Raney-nickel followed by *N*-benzylation opens up a convenient route to lepidilines A and C. Both imidazolium salts were used to generate in situ the corresponding imidazol-2-ylidenes, which smoothly reacted with elemental sulfur, yielding imidazole-2-thiones. These reactions were performed either under classical conditions in pyridine solutions or mechanochemically using solid Cs_2_CO_3_ as a base. The structure of lepidiline C was unambiguously confirmed by X-ray analysis of its hexafluorophosphate. An analogous protocol toward lepidilines B and D and their 4,5-diphenyl analogues is less efficient due to observed instability of the key precursors, i.e., the respective 2-methylimidazole *N*-oxides. Comparison of cytotoxic activity against HL-60 and MCF-7 cell lines of all lepidilines, as well as their selected structural analogues (e.g., 4,5-diphenyl derivatives and PF_6_ salts), revealed slightly more potent activity of the 2-methylated series, irrespectively of the type of counterion present in the imidazolium salt. Remarkably, the well-known 1,3-diadamantylimidazolium bromide (the “Arduengo salt”), known as the precursor of the first, shelf-stable NHC representative, and its adamantyloxy analogue displayed the most significant cytotoxic activity in the studied series.

Lepidilines A–D (**1a**–**1d**) belong to the class of imidazolium alkaloids found in extracts prepared from roots of *Lepidium meyenii* (so-called Maca), a South American plant known in folk medicine of Peruvian Indian tribes for more than a thousand years. Over centuries, aqueous extracts as well as dried roots of Maca were used as a natural drug and as a food additive. Currently it is widely explored as a popular dietary supplement easily available not only in the pharmacy but also in the food markets.^1^ Moreover, lepidilines A and C have been used as convenient precursors of nucleophilic carbenes (NHCs) applied for the synthesis of bioactive metal complexes with gold(I), silver(I), and iridium(I) ions.^2^

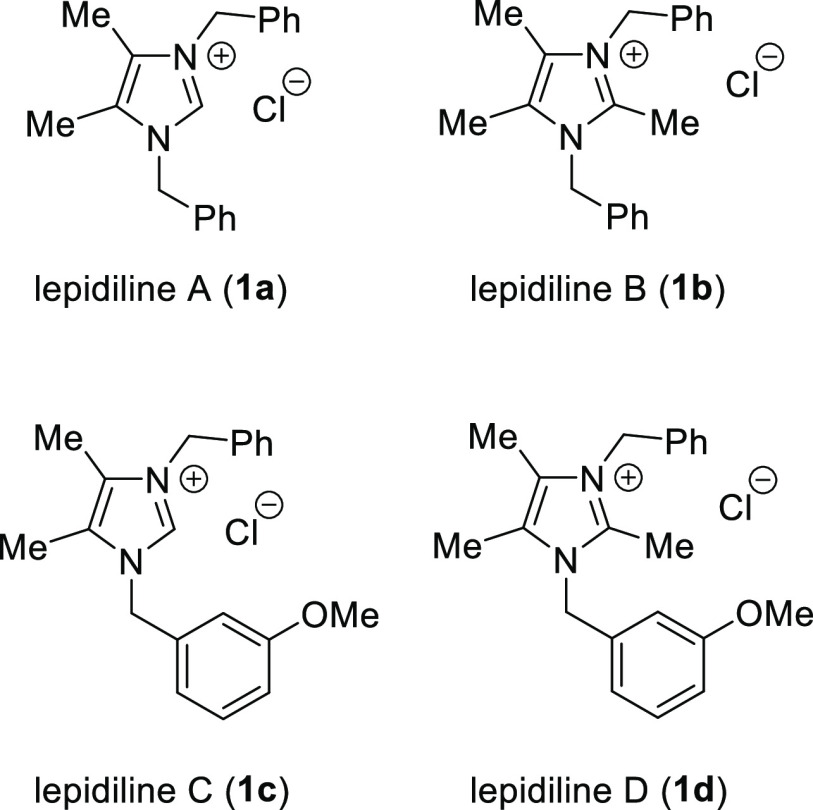



Alkaloids **1a** and **1b** were isolated and identified for the first time in 2003, and the structure of lepidiline A was unambiguously proved by the X-ray analysis.^3^ More recently, two nonsymmetric imidazolium salts, **1c** and **1d** (lepidilines C and D, respectively), were also isolated from Maca extracts, and their structures were elucidated on the basis of spectroscopic data analysis.^4^ Noteworthy, whereas promising anticancer activity of lepidilines A and B was discussed in the original report by Cui et al.,^3^ no information is available on biological properties of lepidilines C and D. Although lepidilines A and B are easily available via double benzylation of 4,5-dimethyl- and 2,4,5-trimethylimidazole,^5^ respectively, for the synthesis of their unsymmetrically substituted analogues C and D the above standard alkylation procedure cannot be applied. Thus, due to the current interest in the chemistry and application of imidazolium salts, the development of general methods for multigram scale synthesis of lepidilines A–D is of practical importance.

In our continuous research on the synthesis and reactivity of imidazole *N*-oxides of type **2**, we demonstrated that they are superior building blocks for the preparation of diverse imidazole derivatives.^6^ As shown in Scheme 1, condensation of α-hydroxyiminoketones **3** with imines **4** followed by deoxygenation of the first formed imidazole *N*-oxides **2** offers a convenient access to polysubstituted imidazoles **5** with excellent control on the substitution pattern. Thus, the method allows for preparation of more complex imidazole derivatives bearing either functionalized alkyl or aryl groups located at the N(1), C(2), C(4), and C(5) atoms of the core heterocycle. In the quest for designed lepidiline precursors, diacetyl monoxime (**3a**, R^3^ = R^4^ = Me) and benzyl formaldimines (R^2^ = H) and acetimines (R^2^ = Me) of type **4** are indicated as convenient starting materials. Benzylation of the key 4,5-dimethylimidazoles **5** should lead to desired alkaloids **1** (Scheme 1).

**Scheme 1:**
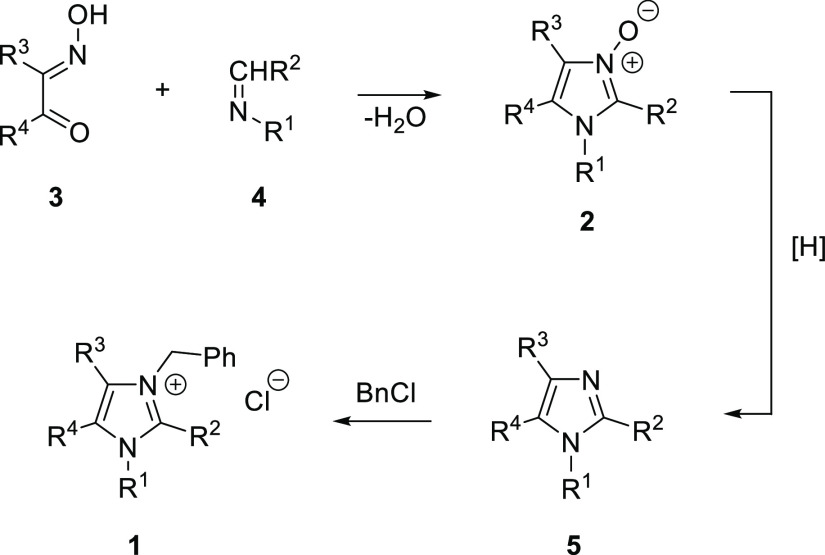
Multistep Synthesis of Lepidilines **1** Starting with Condensation of α-Hydroxyiminoketones **3** with Imines **4**, Followed by Deoxygenation of the Initially Formed Imidazole *N*-Oxides **2** and *N*-Benzylation of the Resulting Imidazoles **5**

The goal of the present study was to elaborate a general method for the preparation of the title compounds and their structural analogues, such as imidazole-2-thiones available via intermediate nucleophilic carbenes. In addition, an unambiguous confirmation of the structure of a representative nonsymmetric alkaloid (lepidiline C or D) is also of interest. Finally, cytotoxic activity of the selected imidazole-based products was tested against two cancer cell lines, HL-60 and MCF-7, and for comparison on normal HUVEC cells.

## Results and Discussion

**Scheme 2:**
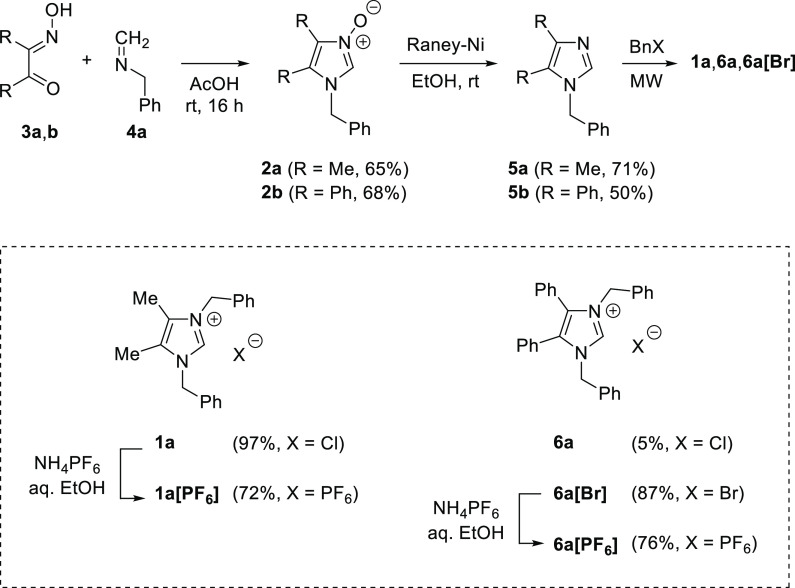
Synthesis of Lepidiline A (**1a**) and Its 4,5-Diphenyl Analogue **6a**

In our recent publication, 2-unsubstituted imidazole *N*-oxides such as **2** were used as key building blocks for the preparation of a series of benzyloxy analogues of lepidiline A.^7^ In the presented study, deoxygenation of *N*-oxides **2** was required to get the desired 1,4,5-tri- and 1,2,4,5-tetrasubstituted imidazoles. In an initial experiment, the known 1-benzyl-4,5-dimethylimidazole *N*-oxide (**2a**)^8^ was treated with freshly prepared Raney-nickel, in EtOH, and the obtained 1-benzyl-4,5-dimethylimidazole (**5a**) was *N*-alkylated with benzyl chloride under microwave (MW) irradiation. The reaction was complete after 5 min, yielding the expected lepidiline A (**1a**) in nearly quantitative yield (Scheme 2). The same method was applied for the synthesis of the 4,5-diphenyl analogue of **1a** (i.e., compound **6a**), but in the case of benzylation with BnCl a very low conversion of ca. 5% was observed after 60 min of heating, while application of BnBr as a more reactive electrophile provided the expected imidazolium bromide **6a[Br]** in 87% yield after 45 min. Furthermore, the anion exchange aimed at preparation of hexafluorophosphates derived from **1a** and **6a[Br]** was easily achieved by treatment of the starting salts with NH_4_PF_6_ in aqueous EtOH.

**Scheme 3:**
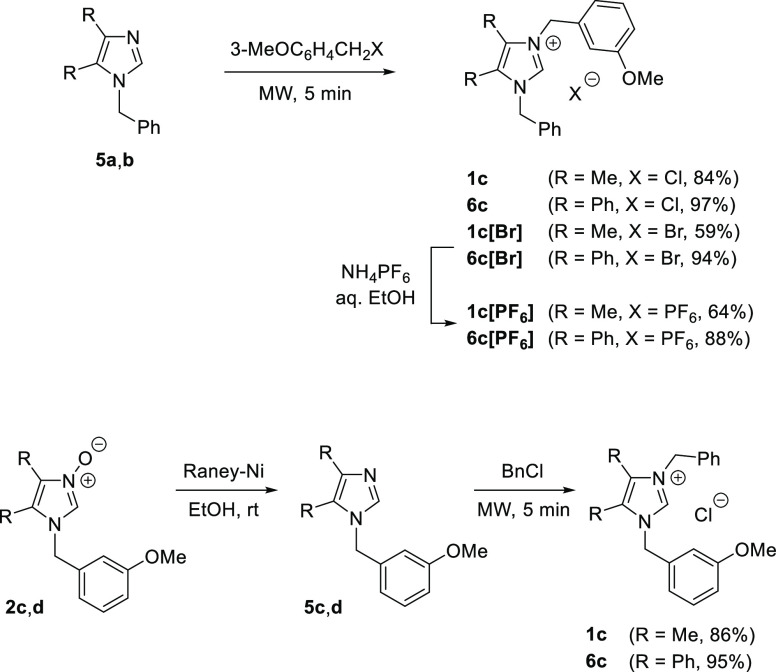
Syntheses of Lepidiline C (**1c**), Its 4,5-Diphenylimidazolium Analogue **6c**, and the Anion Exchange Reactions Leading to the Corresponding Hexafluorophosphates **1c[PF**_**6**_**]** and **6c[PF**_**6**_**]**

**Figure 1:**
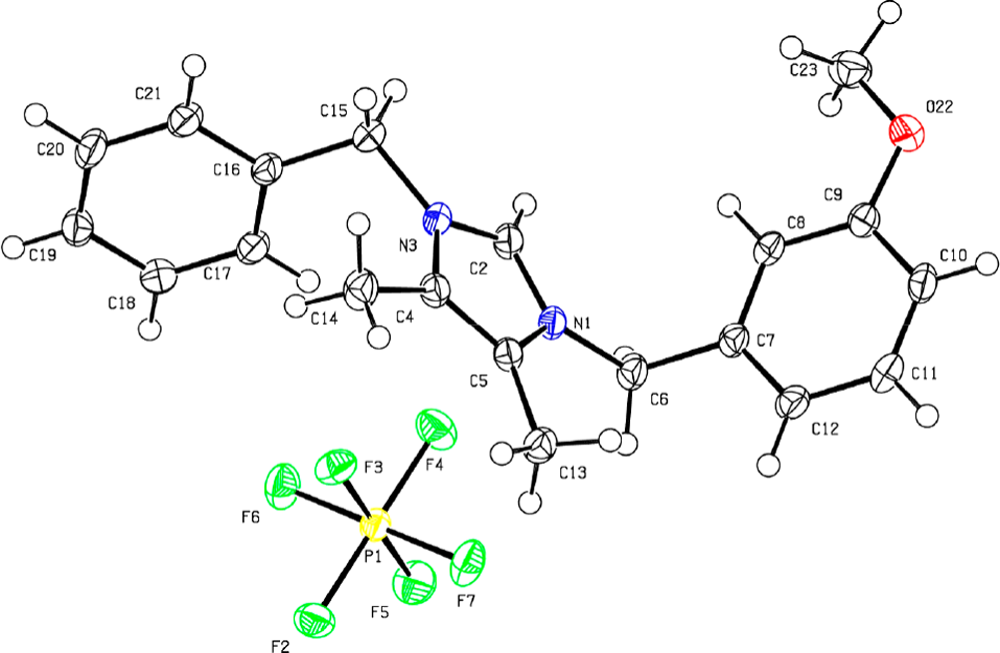
X-ray analysis of imidazolium hexafluorophosphate **1c[PF**_**6**_**]** derived from lepidiline C.

To the best of our knowledge, synthesis of lepidiline C has not yet been elaborated and reported. In our hands, imidazole **5a** was successfully alkylated with *m*-methoxybenzyl chloride under MW conditions to afford the expected imidazolium alkaloid **1c** in 84% yield (Scheme 3). The product was isolated as a viscous oil, which after recrystallization from an *i*-Pr_2_O/CH_2_Cl_2_ mixture gave a colorless solid with a melting point of 94–96 °C. The measured temperature of the Cr → I phase transition in **1c** was clearly different from that reported for lepidiline C isolated from natural sources (mp 225–228 °C). Nevertheless the ^1^H and ^13^C NMR spectra of the obtained material corresponded well with the reported chemical shifts.^4^ Fortunately, the anion exchange in **1c** for PF_6_^–^ enabled growth of fine single crystals suitable for X-ray analysis, which unambiguously confirmed the expected structure of the imidazolium cation in **1c[PF**_**6**_**]** (Figure 1).^9^ By analogy, the 4,5-diphenylimidazolium analogues of lepidiline C, **6c** and **6c[PF**_**6**_**]**, were prepared using imidazole **5b** as the starting material (Scheme 3). Whereas replacement of Cl^–^ by Br^–^ has practically no impact on the chemical shifts of signals in the ^1^H NMR spectra of imidazolium salts of type **1** and **6**, introduction of PF_6_^–^ in the 2-unsubstituted series resulted in a remarkable high-field shift of the diagnostic signals attributed to C(2)-*H*. For example, the aforementioned singlets for **1c**, **1c[Br]**, and **1c[PF**_**6**_**]** were found at δ 10.80, 10.76, and 8.62, respectively.

In order to demonstrate flexibility of the presented synthetic method for the preparation of unsymmetric imidazolium salts, the same lepidiline C and its diphenyl analogue **6c** were prepared in an alternative protocol using 1-(3-methoxybenzyl)-functionalized *N*-oxides **2c** and **2d** (Scheme 3). For example, after smooth deoxygenation of **2c** followed by treatment of the resulting imidazole **5c** with BnCl, the expected **1c** was isolated in fair 54% overall yield (for two steps). These experiments indicate that the preparation of the target unsymmetric imidazolium salts can be readily achieved by using different sets of starting materials, i.e., α-hydroxyiminoketones, benzylamines, and benzyl halides, and thereby, the applied protocol enhances the utility of this method for the preparation of differently substituted imidazolium salts.

**Scheme 4:**
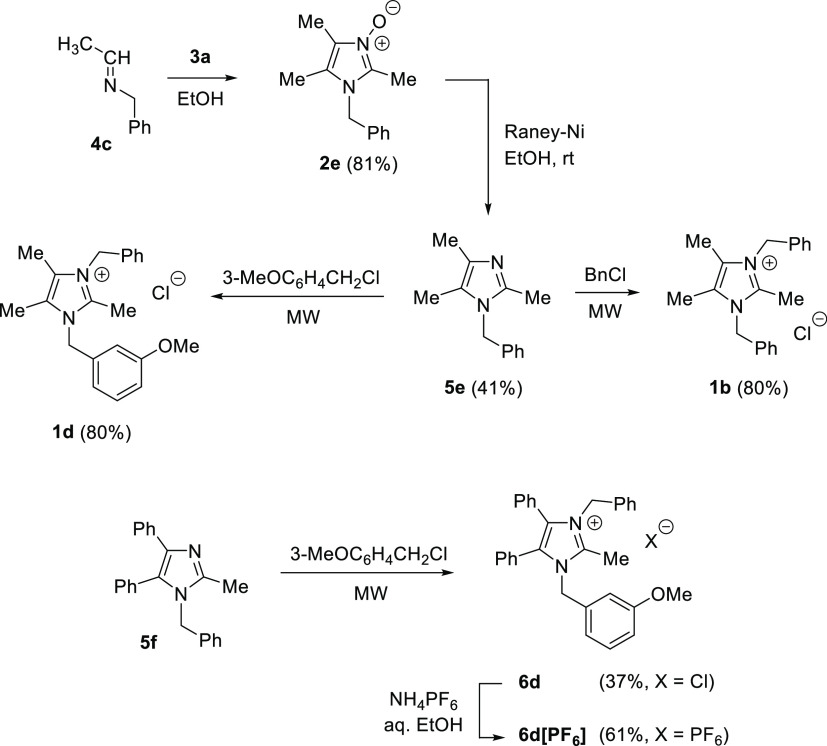
Three-Step Synthesis of Lepidilines B (**1b**) and D (**1d**) Starting with *N*-Benzyl Acetimine (**4c**) and Diacetyl Monoxime (**3a**) (Above) and Benzylation of **5f** Leading to the 4,5-Diphenyl Analogue of Lepidiline D, i*.*e., Imidazolium Salt **6d** (Below)

The replacement of *N*-benzyl formaldimine (**4a**) by the respective acetimine **4c** in the reaction with α-hydroxyiminoketone **3a** opened up a straightforward access to imidazole *N*-oxide **2e** considered as a suitable precursor of lepidilines B and D (Scheme 4). Indeed, reduction of **2e** followed by *N*-benzylation of **5e** with benzyl chloride or *m*-methoxybenzyl chloride provided desired alkaloids **1b** and **1d**, respectively. In contrast to **2e**, attempted preparation of its 4,5-diphenyl analogue using benzil monoxime (**3b**) and acetimine **4c** was unsuccessful, as the initially formed *N*-oxide suffered decomposition during workup. We assume that the observed decomposition of this imidazole *N*-oxide results from the anticipated limited stability of its 3-hydroxy-2-methylidene tautomer. Apparently, the presence of two Ph substituents located at C(4) and C(5) enables the tautomeric rearrangement and in the presence of moisture leads to unidentified, deeply red-colored product(s). For that reason we waved on the synthesis of 1-benzyl-2-methyl-4,5-diphenylimidazole (**5f**) via the respective imidazole *N*-oxide, and instead the required imidazole **5f** was obtained following a known procedure based on multicomponent condensation of benzil, acetaldehyde, benzylamine, and ammonium acetate in the presence of InCl_3_ used as a catalyst.^10^ Thus, the synthesis of the target 4,5-diphenyl analogue of lepidiline D (i.e., compound **6d**) was achieved using the latter heterocycle **5f** and *m*-methoxybenzyl chloride under MW irradiation, which efficiently accelerated the quaternization process.

**Scheme 5:**
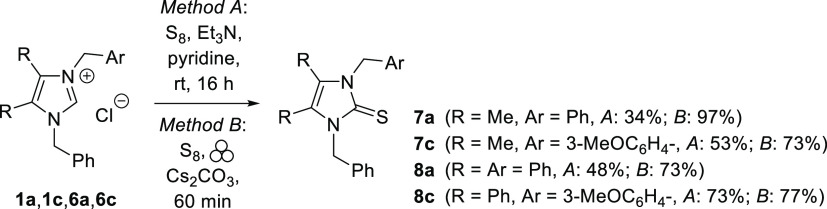
Sulfurization of the *in Situ* Generated Imidazol-2-ylidenes Derived from Lepidilines **1a**,**c** and Their 4,5-Diphenylimidazolium Analogues **6a**,**c** Leading to Non-enolizable Imidazole-2-thiones **7** and **8**

Imidazole-2-thiones are known as biologically active compounds, which display diverse biological activity, and many representatives are recognized as potent antimicrobial, antithyroid, anti-HIV, and anticancer agents.^11^ One of the relatively new and attractive methods for the synthesis of non-enolizable imidazole-2-thiones comprises sulfurization of transient imidazol-2-ylidenes with elemental sulfur.^12^ In a recent report, this method was successfully applied for the conversion of numerous benzyloxy-functionalized imidazolium salts into the corresponding imidazole-2-thiones.^7^ Thus, lepidilines A and C seem to be attractive substrates for further functionalization via the respective intermediate carbenes (NHCs). In a typical experiment, imidazolium chloride **1a** was treated with Et_3_N and S_8_ in dry pyridine solution at room temperature (Scheme 5, Method A). After overnight stirring the expected imidazole-2-thione **7a** was isolated as a crystalline product, although in low 34% yield. In the search for a more efficient protocol, the ball-mill approach was checked by using Cs_2_CO_3_ as a base and a 2-fold excess of elemental sulfur, in the presence of butanone as liquid-assisted grinding solvent (LAGs) (Method B). To our delight, the expected imidazole-2-thione **7a** was formed solely, and the product was isolated in an excellent yield of 97%. The ^13^C NMR spectrum of **7a** confirmed the presence of the thiourea unit in the molecule, as the typical resonance of this diagnostic group was found at δ 162.7. Analogous procedures applied for lepidiline C afforded the respective imidazole-2-thione **7c** isolated as a colorless solid in 53% (Method A) and 73% (Method B) yield. Two more products of that type (i.e., compounds **8a** and **8c**) with a 4,5-diphenylimidazole motif were also obtained in an analogous manner.

Potential biological activity of all four lepidilines A–D (**1a**–**1d**), as well as their 4,5-diphenyl analogues **6a[PF**_**6**_**]**, **6c[PF**_**6**_**]**, and **6d[PF**_**6**_**]** (as hexafluorophosphate salts) and a series of imidazole-2-thiones **7** and **8** obtained therefrom, was evaluated in vitro against two human cancer cell lines: promyelocytic leukemia HL-60 and breast cancer adenocarcinoma MCF-7. Selected analogues were also tested against human umbilical vein endothelial cells (HUVECs) using the MTT cytotoxicity assay. Concentration–response analysis was performed to determine drug concentrations required to inhibit the growth of cells by 50% (IC_50_) after 48 h of incubation. The obtained results are summarized in Table 1 and compared with activity of the known anticancer agent doxorubicin (DXR) used as a positive control.^13^

**Table 1:**
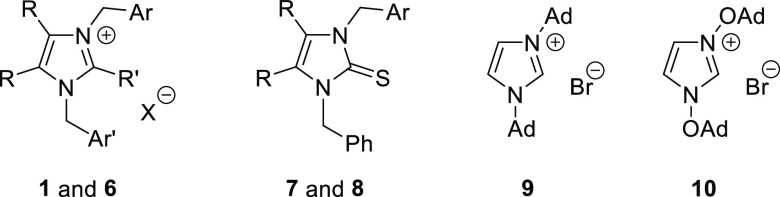
Cell Growth Inhibitory Activity of the Selected Imidazolium Salts **1**, **6**, **9**, and **10** (Ad = Adamantan-1-yl), Imidazole-2-thiones **7** and **8**, and the Reference Doxorubicin (DXR)

							IC_50_ [μM]^a^		
entry	cmpd	R	R′	Ar	Ar′	X	HL-60	MCF-7	HUVEC
1	**1a**	Me	H	Ph	Ph	Cl	32.3 ± 3.5	>100	>100
2	**1b**	Me	Me	Ph	Ph	Cl	3.8 ± 0.2	>100	>100
3	**1c**	Me	H	*m*-MeOC_6_H_4_	Ph	Cl	27.7 ± 2.5	75.0 ± 5.0	>100
4	**1d**	Me	Me	*m*-MeOC_6_H_4_	Ph	Cl	1.1 ± 0.1	>100	>100
5	**1a[PF**_**6**_**]**	Me	H	Ph	Ph	PF_6_	32.0 ± 2.0	>100	
6	**1c[PF**_**6**_**]**	Me	H	*m*-MeOC_6_H_4_	Ph	PF_6_	19.9 ± 0.2	69.9 ± 0.2	
7	**6a[PF**_**6**_**]**	Ph	H	Ph	Ph	PF_6_	1.2 ± 0.1	8.2 ± 0.2	
8	**6c[PF**_**6**_**]**	Ph	H	*m*-MeOC_6_H_4_	Ph	PF_6_	1.2 ± 0.2	7.9 ± 0.3	8.8 ± 0.1
9	**6d[PF**_**6**_**]**	Ph	Me	*m*-MeOC_6_H_4_	Ph	PF_6_	1.2 ± 0.1	6.5 ± 0.2	
10	**7a**	Me	H	Ph			>100	>100	
11	**7c**	Me	H	*m*-MeOC_6_H_4_			20.2 ± 1.9	>100	
12	**8a**	Ph	H	Ph			8.1 ± 0.3	>100	
13	**8c**	Ph	H	*m*-MeOC_6_H_4_			55 ± 5	>100	
14	**9**						1.8 ± 0.1	51.5 ± 1.5	
15	**10**						0.3 ± 0.1	4.5 ± 0.1	9.1 ± 0.3
16	**DXR**						0.12 ± 0.01	0.9 ± 0.1	1.4 ± 0.1

a Compound concentration required to inhibit metabolic activity by 50%. The cells were incubated with the analogues for 48 h. Values are expressed as mean ± SEM from the concentration–response curves of at least three experiments using a nonlinear estimation (quasi-Newton algorithm) method.

Lepidilines B and D were found to be significantly cytotoxic against HL-60 cells with IC_50_ values in the low micromolar range of 3.8 and 1.1 μM, respectively, which were an order of magnitude lower than those obtained for lepidilines A and C (i.e., 32.3 and 27.7 μM, respectively). None of the lepidilines showed similarly high cytotoxicity on the MCF-7 cell line, and lepidiline D turned out to be over 100-fold more cytotoxic for leukemia than for breast cancer cells. In the experiments performed on normal (HUVEC) cells the IC_50_ values for all four lepidilines A–D were over 100 μM, which indicates a large safety margin for these compounds.

Replacement of the chloride counterion in lepidilines A and C by hexafluorophosphate (i.e., compounds **1a[PF**_**6**_**]** and **1c[PF**_**6**_**]**, respectively) did not result in increased activity (Table 1, entries 5 and 6).

It is worth stressing that this is the first report in which the cytotoxicity of all four representatives of the lepidiline family has been checked against selected cell lines, which made it possible to compare their activity under identical conditions. In a previous report by Zheng only lepidilines A and B were examined against eight human cancer cell lines.^3^ The mentioned work indicated lepidiline A was inactive against all those cell lines with IC_50_ values above 10 μM (the values reported in the paper are expressed in μg/mL). In contrast, lepidiline B showed cytotoxic activity against several cell lines, especially high against pancreatic adenocarcinoma PACA2 (IC_50_ = 1.38 μg/mL, i.e., 4.2 μM) and against breast carcinoma MDA-231 (IC_50_ = 1.66 μg/mL, i.e., 5.1 μM). Unfortunately, it is difficult to compare those results with our data obtained on different cell lines. However, the common observation from both studies is that lepidiline B is more cytotoxic than lepidiline A.

Analysis of the structure–activity relationship of 4,5-diphenyl analogues of lepidilines A, C, and D (Table 1, entries 7–9) revealed that the replacement of methyl by phenyl at C(4) and C(5) of the imidazole ring caused a large increase of cytotoxicity against the MCF-7 cell line and in the case of **6c[PF**_**6**_**]** also against HUVECs, making these analogues much less selective as compared with natural lepidilines.

Introduction of a sulfur atom at C(2) in a series of imidazole-2-thiones **7** and **8** derived from lepidilines A and C and their analogues bearing Ph substituents at C(4) and C(5) atoms of the imidazole ring was not advantageous for activity, especially against MCF-7 cells (Table 1, entries 10–13). This decreased cytotoxicity may result from lower bioavailability of imidazole-2-thiones, which in contrast to lepidilines are not charged molecules. It is well known that the neutral organic compounds interact with membranes only through hydrophobic bonds, whereas charged substances can additionally benefit from electrostatic interactions.^14^

In contrast to imidazole-2-thiones, two highly oleophilic imidazolium salts **9** and **10** bearing at N(1) and N(3) atoms either adamantan-1-yl (the “Arduengo salt”)^15^ or adamantan-1-yloxy groups,^16^ respectively, were found to be significantly cytotoxic for HL-60 cells. Notably, imidazolium salt **10** was the most active among all tested compounds (IC_50_ = 0.3 and 4.5 μM on HL-60 and MCF-7 cell lines, respectively) and also displayed some selectivity.

As a positive control in the MTT assay a well-known anticancer drug, DXR, widely used to treat breast cancer and acute lymphocytic leukemia among other cancer types,^13^ has been used. The IC_50_ values for DXR against HL-60 and MCF-7 cells were below 1 μM (0.12 and 0.9 μM against HL-60 and MCF-7 cells, respectively), and the value obtained for normal HUVEC cells was 1.4 μM under the same experimental conditions. Thus, DXR was 10-fold more cytotoxic than lepidiline D, but the HUVEC/HL-60 IC_50_ ratio for DXR was about 10 and that for lepidiline D over 100.

In summary, the present study showed that the title lepidilines A–D can be conveniently prepared using imidazole *N*-oxides as key intermediates, which are readily available via cyclocondensation of diacetyl monoxime with *N*-benzyl aldimines. Initial deoxygenation with Raney-nickel followed by microwave-assisted benzylation leads to both symmetric and nonsymmetric imidazolium alkaloids in excellent yield and purity. X-ray analysis of the imidazolium salt derived from synthetic lepidiline C was presented for the first time and confirmed the postulated structure of the naturally occurring material. In addition, sulfurization of nucleophilic carbenes derived from lepidilines A and C enables convenient preparation of corresponding, hitherto unknown, non-enolizable imidazole-2-thiones.

Our results contribute to the development of methods useful for synthesis of naturally occurring, biologically active imidazole derivatives relevant for medicinal chemistry and related applications.^17,18^ Progress in this attractive field was summarized in a very recent, comprehensive review.^19^ Further applications of imidazolium salts of “lepidiline type” in coordination, organometallic, and materials chemistry are also possible.^18,19^

The biological results presented here revealed significant cytotoxicity of lepidilines B and D in the tested series of naturally occurring alkaloids and, most importantly, their remarkable selectivity against leukemia HL-60 versus normal HUVEC cells. These compounds as well as their 4,5-diphenyl imidazolium derivatives and the presented adamantyloxy analogue of the “Arduengo salt”^15^ can be considered not only as readily available precursors of nucleophilic carbenes^2^ but also as useful probes in the search for new leads in antileukemic drug discovery.

## Experimental Section

### General Experimental Procedures

Melting points were determined in capillaries with a MEL-TEMP apparatus (Aldrich) and are uncorrected. The IR spectra were measured neat with an Agilent Cary 630 FTIR spectrometer. NMR spectra were measured on a Bruker AVIII instrument (^1^H at 600 MHz, ^13^C at 151 MHz). Chemical shifts are reported relative to residual nondeuterated solvent signals (for CDCl_3_: ^1^H NMR: δ 7.26, ^13^C NMR: δ 77.16; for acetone-*d*_6_: ^1^H NMR: δ 2.09, ^13^C NMR: δ 30.60).^20^ MS (ESI) was performed with a Varian 500-MS LC ion trap; high-resolution MS (ESI-TOF) measurements were performed with a Synapt G2-Si mass spectrometer (Waters). Nonionic products were purified by standard column chromatography (CC) on silica gel (230–400 mesh) or by preparative thin-layer chromatography (PTLC); organic salts were purified by recrystallization from *i*-Pr_2_O or hexanes/CH_2_Cl_2_ mixtures. Mechanochemical reactions were performed by using a Retsch Mixer Mill MM400. Elemental analyses were obtained with a Vario EL III (Elementar Analysensysteme GmbH) instrument. Commercially available solvents and starting materials were used as received. Starting materials, i.e., diacetyl monoxime (**3a**),^21^ benzil monoxime (**3b**),^22^ formaldimine **4a**(23) (in the form of a trimer, i.e., 1,3,5-tribenzylhexahydro-1,3,5-triazine), and acetimine **4c**(24) (monomeric) were prepared following the general literature procedures.

### Synthesis of Imidazolium Chlorides and Bromides of Type **1** and **6**

To a deoxygenated solution of imidazole **5** (1.0 mmol) in MeCN (10 mL) was added benzyl halide (1.5 mmol), and the resulting mixture was MW-irradiated at 110 °C until the starting imidazole was fully consumed (TLC monitoring, usually up to 60 min). The solvent was removed under reduced pressure, and the crude product was washed with several portions of dry Et_2_O. Solid products were recrystallized from a CH_2_Cl_2_/hexanes mixture.

#### 1,3-Dibenzyl-4,5-dimethylimidazolium chloride (**1a**, lepidiline A):

303 mg (97%); colorless crystals; mp 246–248 °C; IR (neat) ν 2878, 2184, 1633, 1558, 1532, 1454, 1361, 1219 cm^–1^; ^1^H NMR (600 MHz, CDCl_3_) δ 11.10* (s, 1H), 7.32–7.26 (m, 10H), 5.49 (s, 4H), 2.03 (s, 6H); *partial H/D exchange observed.; ^13^C NMR (151 MHz, CDCl_3_) δ 137.4*, 133.3 (2C), 129.4 (4C), 128.9 (2C), 127.9 (4C), 127.2 (2C), 51.1 (2C), 8.9 (2C); *broadened due to partial H/D exchange; anal. calcd for C_19_H_21_ClN_2_ (312.1) C 72.95, H 6.77, N 8.95; found C 72.94, H 6.79, N 9.02.

#### 1,3-Dibenzyl-2,4,5-trimethylimidazolium chloride (**1b**, lepidiline B):

261 mg (80%); colorless crystals; mp 228–229 °C; IR (neat) ν 3474, 3414, 1521, 1480, 1454, 1428, 1364 cm^–1^; ^1^H NMR (600 MHz, CDCl_3_) δ 7.34–7.26 (m, 6H), 7.07–7.04 (m, 4H), 5.53 (s, 4H), 2.74 (s, 3H), 2.19 (s, 6H); ^13^C NMR (151 MHz, CDCl_3_) δ 144.2, 133.3 (2C), 129.5 (4C), 128.6 (2C), 126.4 (2C), 126.1 (4C), 49.2 (2C), 11.7, 9.1 (2C); anal. calcd for C_20_H_23_ClN_2_·H_2_O (344.2) C 69.65, H 7.31, N 8.12; found C 69.15, H 7.13, N 8.61.

#### 1-Benzyl-3-(3-methoxybenzyl)-4,5-dimethylimidazolium chloride (**1c**, lepidiline C):

303 mg (84%) from **5a** and 310 mg (86%) from **5c**; colorless solid; mp 94–96 °C; IR (neat) ν 3399, 1588, 1551, 1491, 1454, 1290, 1260, 1155, 1051 cm^–1^; ^1^H NMR (600 MHz, CDCl_3_) δ 10.80 (s, 1H), 7.31–7.24 (m, 5H), 7.22–7.19 (m, 1H), 6.85–6.83 (m, 1H), 6.81–6.78 (m, 2H), 5.48 (s, 2H), 5.44 (s, 2H), 3.74 (s, 3H), 2.03 (s, 3H), 2.02 (s, 3H); ^13^C NMR (151 MHz, CDCl_3_) δ 160.2, 137.2, 134.8, 133.3, 130.3, 129.3 (2C), 128.8, 127.8 (2C), 127.2, 127.1, 119.8, 114.5, 113.2, 55.6, 51.0, 50.9, 8.8 (2C); anal. calcd for C_20_H_23_ClN_2_O·H_2_O (360.1) C 66.56, H 6.98, N 7.76; found C 65.83, H 6.79, N 7.31.

#### 1-Benzyl-3-(3-methoxybenzyl)-4,5-dimethylimidazolium bromide (**1c[Br]**):

brown oil, 228 mg (59%) from **5a**; IR (neat) ν 2952, 1558, 1491, 1454, 1260, 1211, 1156, 1051 cm^–1^; ^1^H NMR (600 MHz, CDCl_3_) δ 10.76 (s, 1H), 7.34–7.28 (m, 5H), 7.24–7.21 (m, 1H) 6.89 (m_c_, 1H), 6.84–6.81 (m, 2H), 5.49 (s, 2H), 5.46 (s, 2H), 3.77 (s, 3H), 2.07 (s, 3H), 2.06 (s, 3H); ^13^C NMR (151 MHz, CDCl_3_) δ 160.3, 136.6, 134.6, 133.1, 130.4, 129.4 (2C), 129.0, 127.9 (2C), 127.4, 127.3, 119.9, 114.8, 113.3, 55.7, 51.1, 51.0, 8.9.

#### 1-Benzyl-3-(3-methoxybenzyl)-2,4,5-trimethylimidazolium chloride (**1d**, lepidiline D):

285 mg (80%) from **5e**; colorless crystals; mp 214–216 °C; IR (neat) ν 1603, 1461, 1256, 1167, 1036 cm^–1^; ^1^H NMR (600 MHz, CDCl_3_) δ 7.34–7.21 (m, 4H), 7.07–7.04 (m, 2H), 6.81–6.78 (m, 1H), 6.59–6.54 (m, 2H), 5.52 (s, 2H), 5.51 (s, 2H), 3.74 (s, 3H), 2.75 (s, 3H), 2.19 (s, 3H), 2.18 (s, 3H); ^13^C NMR (151 MHz, CDCl_3_) δ 160.32, 144.3, 134.9, 133.2, 130.6, 129.5 (2C), 128.7, 126.4, 126.3, 126.1 (2C), 118.0, 113.7, 112.1, 55.5, 49.2, 49.1, 11.7, 9.13, 9.11; anal. calcd for C_21_H_25_ClN_2_O (356.2) C 70.67, H 7.06, N 7.85; found C 70.69, H 7.12, N 7.90.

#### 1,3-Dibenzyl-4,5-diphenylimidazolium bromide (**6a[Br]**):

466 mg (87%); pale yellow solid; mp 220–222 °C; IR (neat) ν 3027, 1558, 1491, 1450, 1349, 1211, 1178, 1025 cm^–1^; ^1^H NMR (600 MHz, CDCl_3_) δ 11.18 (s, 1H), 7.40–7.36 (m, 2H), 7.32–7.28 (m, 4H), 7.24–7.19 (m, 6H), 7.11–7.06 (m, 8H), 5.49 (s, 4H); ^13^C NMR (151 MHz, CDCl_3_) δ 137.5, 133.3 (2C), 132.2 (2C), 130.8 (4C), 130.5 (2C), 129.13 (4C), 129.12 (4C), 129.0 (2C), 128.6 (4C), 124.7 (2C), 51.5 (2C); anal. calcd for C_30_H_27_BrN_2_·0.5CH_2_Cl_2_ (536.1) C 67.63, H 5.00, N 5.35; found C 67.60, H 5.13, N 5.47.

#### 1-Benzyl-3-(3-methoxybenzyl)-4,5-diphenylimidazolium chloride (**6c**):

452 mg (97%); colorless solid; mp 192–194 °C; IR (neat) ν 1603, 1551, 1491, 1443, 1267, 1182, 1036 cm^–1^; ^1^H NMR (600 MHz, CDCl_3_) δ 11.24 (s, 1H), 7.42–7.38 (m, 2H), 7.33–7.29 (m, 4H), 7.25–7.20 (m, 3H), 7.14–7.06 (m, 7H), 6.79–6.77 (m, 1H), 6.73 (m_c_, 1H), 6.63–6.60 (m, 1H), 5.49 (s, 2H) 5.45 (s, 2H), 3.73 (s, 3H); ^13^C NMR (151 MHz, CDCl_3_) δ 160.1, 137.7, 134.8, 133.4, 132.3, 132.2, 130.9 (2C), 130.8 (2C), 130.5 (2C), 130.2, 129.2 (4C), 129.1 (2C), 129.0, 128.6 (2C), 124.8, 124.7, 120.6, 115.5, 113.4, 55.7, 51.5, 51.4.

#### 1-Benzyl-3-(3-methoxybenzyl)-4,5-diphenylimidazolium bromide (**6c[Br]**):

496 mg (94%); off-white solid; mp 149–151 °C; IR (neat) ν 1603, 1551, 1491, 1454, 1264, 1182, 1036 cm^–1^; ^1^H NMR (600 MHz, CDCl_3_) δ 11.03 (s, 1H), 7.41–7.36 (m, 2H), 7.33–7.28 (m, 4H), 7.25–7.19 (m, 3H), 7.14–7.06 (m, 7H), 6.79–6.76 (m, 1H), 6.72 (m_c_, 1H), 6.63–6.59 (m, 1H), 5.48 (s, 2H), 5.45 (s, 2H), 3.72 (s, 3H); ^13^C NMR (151 MHz, CDCl_3_) δ 160.0, 137.4, 134.8, 133.4, 132.3, 132.2, 130.9 (2C), 130.8 (2C), 130.5 (2C), 130.1, 129.10 (4C), 129.08 (2C), 129.0, 128.5 (2C), 124.73, 124.71, 120.6, 115.3, 113.4, 55.7, 51.5, 51.4; anal. calcd for C_30_H_27_BrN_2_O·H_2_O (528.2) C 68.05, H 5.52, N 5.29; found C 68.11, H 5.42, N 5.39.

#### 1-Benzyl-3-(3-methoxybenzyl)-2-methyl-4,5-diphenylimidazolium chloride (**6d**)

The crude product was purified by preparative thin-layer chromatography (SiO_2_, CH_2_Cl_2_/MeOH, 92:8); spectroscopically pure sample of **6d** (178 mg, 37%) was isolated as a colorless oil and used for the next step without further purification. ^1^H NMR (600 MHz, CDCl_3_) δ 7.39–7.36 (m, 2H), 7.34–7.22 (m, 12H), 7.04–7.01 (m, 2H), 6.83–6.80 (m, 1H), 6.60–6.58 (m, 1H), 6.53 (m_c_, 1H), 5.54 (s, 2H), 5.52 (s, 2H), 3.74 (s, 3H), 2.82 (s, 3H); ^13^C NMR (151 MHz, CDCl_3_) δ 160.3, 136.0, 135.6, 133.9, 132.3, 132.2, 131.0 (4C), 130.6, 130.5 (2C), 129.5 (2C), 129.2 (4C), 128.7, 126.4 (2C), 125.17, 125.15, 118.3, 113.9, 112.2, 55.5, 49.74, 49.67, 12.8.

### Synthesis of Imidazolium Hexafluorophosphates **1[PF**_**6**_**]** and **6[PF**_**6**_**]**

To a solution of imidazolium chloride or bromide of type **1** or **6** (0.30 mmol) in EtOH (1.0 mL) was added dropwise a solution of NH_4_PF_6_ (54 mg, 0.33 mmol) in H_2_O (1.0 mL), and the mixture was stirred for 30 min. The crude oily or crystalline product was isolated, washed with dry Et_2_O (3 × 4 mL), and recrystallized from a CH_2_Cl_2_/*i*-Pr_2_O mixture (by slow evaporation of the solvents at room temperature).

#### 1,3-Dibenzyl-4,5-dimethylimidazolium hexafluorophosphate (**1a[PF**_**6**_**]**):

93 mg (72%) from **1a**; colorless solid; mp 142–144 °C; IR (neat) ν 1566, 1454, 1357, 1215, 824 cm^–1^; ^1^H NMR (600 MHz, acetone-*d*_6_) δ 9.13 (s, 1H), 7.50–7.43 (m, 10H), 5.60 (s, 4H), 2.31 (s, 6H); ^13^C NMR (151 MHz, acetone-*d*_6_) δ 136.6, 135.5 (2C), 130.9 (4C), 130.4 (2C), 129.8 (2C), 129.5 (4C) 52.1 (2C), 9.4 (2C); anal. calcd for C_19_H_21_F_6_N_2_P·0.5H_2_O (431.1) C 52.90, H 5.14, N 6.49; found C 52.94, H 5.03, N 6.87.

#### 1-Benzyl-3-(3-methoxybenzyl)-4,5-dimethylimidazolium hexafluorophosphate (**1c[PF**_**6**_**]**):

87 mg (64%) from **1c[Br]**; pale yellow crystals; mp 102–104 °C; IR (neat) ν 1603, 1566, 1457, 1264, 1185, 1036, 828 cm^–1^; ^1^H NMR (600 MHz, CDCl_3_) δ 8.62 (s, 1H), 7.38–7.32 (m, 3H), 7.29–7.26 (m, 1H), 7.24–7.22 (m, 2H), 6.89–6.86 (1H), 6.81–6.78 (m, 2H), 5.24 (s, 2H), 5.20 (s, 2H), 3.79 (s, 3H), 2.10 (s, 3H), 2.08 (s, 3H); ^13^C NMR (151 MHz, CDCl_3_) δ 160.5, 134.5, 134.1, 132.7, 130.6, 129.5 (2C), 129.2, 128.2, 128.0, 127.8 (2C), 120.0, 115.2, 113.0, 55.5, 51.2 (2C), 8.66, 8.65; anal. calcd for C_20_H_23_F_6_N_2_OP (452.1) C 53.10, H 5.12, N 6.19; found C 53.11, H 5.18, N 6.21.

#### 1,3-Dibenzyl-4,5-diphenylimidazolium hexafluorophosphate (**6a[PF**_**6**_**]**):

127 mg (76%) from **6a[Br]**; colorless solid; mp 129–131 °C; IR (neat) ν 1551, 1446, 1182, 1077, 1018, 828 cm^–1^; ^1^H NMR (600 MHz, CDCl_3_) δ 8.73 (s, 1H), 7.40–7.37 (m, 2H), 7.33–7.23 (m, 10H), 7.20–7.17 (m, 4H), 7.05–7.02 (m, 4H), 5.24 (s, 4H); ^13^C NMR (151 MHz, CDCl_3_) δ 137.4, 133.0 (2C), 132.8 (2C), 131.0 (4C), 130.5 (2C), 129.3 (4C), 129.2 (2C), 129.1 (4C), 128.6 (4C), 124.8 (2C), 51.9 (2C); anal. calcd for C_29_H_25_F_6_N_2_P·0.5H_2_O (555.2) C 62.70, H 4.72, N 5.04; found C 62.40, H 4.68, N 5.31.

#### 1-Benzyl-3-(3-methoxybenzyl)-4,5-diphenylimidazolium hexafluorophosphate (**6c[PF**_**6**_**]**):

152 mg (88%) from **6c[Br]**; colorless crystals; mp 78–81 °C; IR (neat) ν 1603, 1558, 1491, 1454, 1267, 1185, 1036, 828 cm^–1^; ^1^H NMR (600 MHz, CDCl_3_) δ 8.73 (s, 1H), 7.41–7.14 (m, 14H), 7.04–7.01 (m, 2H), 6.81–6.78 (m, 1H), 6.61–6.57 (m, 2H), 5.23 (s, 2H), 5.20 (s, 2H), 3.70 (s, 3H); ^13^C NMR (151 MHz, CDCl_3_) δ 160.2, 135.3, 134.1, 132.95, 132.91, 132.8, 131.01 (2C), 130.96 (2C), 130.5 (2C), 130.4, 129.3 (2C), 129.2, 129.14 (2C), 129.12 (2C), 128.5 (2C), 124.82, 124.79, 120.8, 115.6, 113.4, 55.5, 51.92, 51.90; anal. calcd for C_30_H_27_F_6_N_2_OP (576.2) C 62.50, H 4.72, N 4.86; found C 62.48, H 4.61, N 4.87.

#### 1-Benzyl-3-(3-methoxybenzyl)-2-methyl-4,5-diphenylimidazolium hexafluorophosphate (**6d[PF**_**6**_**]**):

116 mg (61%) from **6d**; colorless solid; mp 175–177 °C; IR (neat) ν 1614, 1584, 1495, 1439, 1349, 1275, 1148, 1047, 831 cm^–1^; ^1^H NMR (600 MHz, CDCl_3_) δ 7.38–7.34 (m, 4H), 7.32–7.27 (m, 10H), 6.99–6.97 (m, 2H), 6.85–6.83 (m, 1H), 6.57–6.55 (m, 1H), 6.47 (m_c_, 1H), 5.28 (s, 2H), 5.26 (s, 2H), 3.75 (s, 3H), 2.51 (s, 3H); ^13^C NMR (151 MHz, CDCl_3_) δ 160.4, 144.8, 135.3, 133.6, 132.5, 132.4, 131.17 (2C), 131.16 (2C), 130.8, 130.5 (2C), 129.6 (2C), 129.2 (4C), 128.8, 126.2 (2C), 125.2 (2C), 118.2, 114.0, 112.0, 55.5, 49.4, 49.3, 11.4; anal. calcd for C_31_H_29_F_6_N_2_OP·0.5CH_2_Cl_2_ (632.2) C 59.77, H 4.78, N 4.43; found C 59.72, H 4.74, N 4.64.

### Synthesis of Imidazole *N*-oxides **2**

To a solution of appropriate α-hydroxyiminoketone of type **3** (9.0 mmol) in glacial acetic acid (20 mL) was added a portion of formaldimine 4 (10 mmol), and the resulting mixture was stirred at room temperature overnight. Then, excess concentrated HCl was added (4 mL), the solvents were removed under reduced pressure, and the resulting product was dissolved in MeOH. After excess solid NaHCO_3_ was added the stirring was continued for 2 h until the evolution of CO_2_ ceased. The solvent was removed in vacuo, the residue was triturated with CH_2_Cl_2_, the precipitate salts were filtered off, the solvent was evaporated, and the residue was washed with a few portions of Et_2_O to give imidazole *N*-oxides **2**, which were used for the next step without further purification.

#### 1-Benzyl-4,5-dimethylimidazole *N*-oxide (**2a**; 1.18 g, 65%) and 1-benzyl-4,5-diphenylimidazole *N*-oxide (**2b**; 2.00 g, 68%):

colorless crystals; spectroscopic analysis (^1^H and ^13^C NMR) of both products were in a full agreement with the literature data.^8^

#### 1-(3-Methoxybenzyl)-4,5-dimethylimidazole 3-oxide (**2c**):

1.71 g (82%); colorless solid; mp 157–160 °C; IR (neat) ν 3120, 1584, 1439, 1375, 1334, 1286, 1244, 1148, 1051 cm^–1^; ^1^H NMR (600 MHz, CDCl_3_) δ 7.78 (s, 1H), 7.25–7.21 (m, 1H), 6.83–6.80 (m, 1H), 6.63–6.60 (m, 1H), 6.57 (m_c_, 1H), 4.90 (s, 2H), 3.73 (s, 3H), 2.15 (s, 3H), 2.03 (s, 3H); ^13^C NMR (151 MHz, CDCl_3_) δ 160.3, 136.1, 130.4, 127.4, 124.7, 121.3, 119.0, 113.7, 112.7, 55.4, 49.4, 8.9, 7.4; ESI-MS (*m*/*z*) 233.2 (100, [M + H]^+^), 217 (18); anal. calcd for C_13_H_16_N_2_O_2_ (232.1) C 67.22, H 6.94, N 12.06; found C 67.16, H 6.89, N 12.00.

#### 1-(3-Methoxybenzyl)-4,5-diphenylimidazole 3-oxide (**2d**):

2.34 g (73%); colorless solid; mp 195–197 °C; IR (neat) ν 1599, 1338, 1264, 1167, 1029 cm^–1^; ^1^H NMR (600 MHz, CDCl_3_) δ 7.96 (s, 1H), 7.57–7.55 (m, 2H), 7.43–7.35 (m, 3H), 7.27–7.19 (m, 6H), 6.85–6.83 (m, 1H), 6.66–6.63 (m, 1H), 6.55 (m_c_, 1H), 4.90 (s, 2H), 3.74 (s, 3H); ^13^C NMR (151 MHz, CDCl_3_) δ 160.2, 136.2, 130.9 (2C), 130.8, 130.4, 129.69, 129.65 (2C), 129.2 (2C), 128.20, 128.17 (2C), 127.6, 127.4, 127.0, 126.1, 119.7, 114.1, 113.3, 55.4, 49.9; ESI-MS (*m*/*z*) 357.2 (100, [M + H]^+^); anal. calcd for C_23_H_20_N_2_O_2_ (356.2) C 77.51, H 5.66, N 7.86; found C 77.36, H 5.57, N 7.94.

#### 1-Benzyl-2,4,5-trimethylimidazole 3-oxide (**2e**)

This compound was prepared by a modified procedure as follows: To a solution of diacetyl monooxime (**3a**, 203 mg, 2.0 mmol) in EtOH (4 mL) was added an excess of freshly prepared formaldimine **4c** in two portions (first portion: 532 mg, 4.0 mmol; the second portion of 133 mg, 1.0 mmol was added after 24 h), and the resulting mixture was stirred at room temperature for 48 h. The solvent was removed and the crude **2e** was used for next step without purification. Colorless oil, 350 mg (∼81%); ^1^H NMR (600 MHz, CDCl_3_) δ 7.33–7.28 (m, 3H), 6.95–6.92 (m, 2H), 5.00 (s, 2H), 2.39 (s, 3H), 2.21 (s, 3H), 2.06 (s, 3H); ^13^C NMR (151 MHz, CDCl_3_) δ 135.1, 129.3 (2C), 128.3, 127.3, 125.7 (2C), 125.6, 119.2, 47.4, 8.82, 8.44, 7.71.

### Synthesis of Formaldimine **4b**

A mixture of 3-methoxybenzylamine (5.0 g, 36.5 mmol) and aqueous formaldehyde (37%, 3.26 g, 40.0 mmol) in benzene (60 mL) was refluxed in a Dean–Stark apparatus for 1.5 h. The solvent was removed under reduced pressure to give a thick yellow oil (5.3 g, 97%) identified as the trimeric form of **4b**, which was used for the next step without further purification.

#### 1,3,5-Tri(3-methoxybenzyl)hexahydro-1,3,5-triazine (trimer of **4b**):

^1^H NMR (600 MHz, CDCl_3_) δ 7.21–7.18 (m, 3H), 6.94–6.91 (m, 6H), 6.79–6.77 (m, 3H), 3.80 (s, 9H), 3.67 (s, 6H), 3.43 (s_br_, 6H); ^13^C NMR (151 MHz, CDCl_3_) δ 157.9, 140.3, 129.3, 121.2, 114.2, 112.7, 73.9, 57.1, 55.3; anal. calcd for C_27_H_33_N_3_O_3_ (447.3) C 72.46, H 7.43, N 9.39; found C 72.48, H 7.38, N 9.45.

### Synthesis of Imidazoles **5**

To a solution of imidazole *N*-oxide **2** (2.0 mmol) in MeOH (5.0 mL) was added portionwise an excess of freshly prepared suspension of Raney-nickel in MeOH. The resulting mixture was stirred at room temperature until the starting *N*-oxide was fully consumed (monitored by TLC; typically ca. 1 h). The solids were filtered off and the solvent was removed in vacuo to give spectroscopically pure imidazole **5**.

#### 1-Benzyl-4,5-dimethylimidazole^8^ (**5a**; 242 mg, 71%) and 1-benzyl-4,5-diphenylimidazole^25^ (**5b**; 310 mg, 50%):

colorless solids; spectroscopic analysis (^1^H NMR) of both products were in full agreement with the literature data.

#### 1-(3-Methoxybenzyl)-4,5-dimethylimidazole (**5c**):

colorless semisolid; 263 mg (61%); IR (neat) ν 1584, 1490, 1446, 1260, 1238, 1152, 1051, 775 cm^–1^; ^1^H NMR (600 MHz, CDCl_3_) δ 7.38 (s, 1H), 7.24–7.20 (m, 1H), 6.81–6.78 (m, 1H), 6.62–6.60 (m, 1H), 6.55 (m_c_, 1H), 4.95 (s, 2H), 3.74 (s, 3H), 2.15 (s, 3H), 1.99 (s, 3H); ^13^C NMR (151 MHz, CDCl_3_) δ 160.1, 138.3, 135.5, 134.4, 130.0, 122.5, 118.9, 113.0, 112.5, 55.3, 48.6, 12.9, 8.5; ESI-MS (*m*/*z*) 217.3 (100, [M + H]^+^); anal. calcd for C_13_H_16_N_2_O·0.25H_2_O (220.6) C 70.72, H 7.53, N 12.69; found C 70.67, H 7.37, N 12.96.

#### 1-(3-Methoxybenzyl)-4,5-diphenylimidazole (**5d**):

367 mg (54%); colorless solid; mp 86–88 °C; IR (neat) ν 1603, 1491, 1435, 1256, 1159, 1036 cm^–1^; ^1^H NMR (600 MHz, CDCl_3_) δ 7.65 (s, 1H), 7.52–7.49 (m, 2H), 7.41–7.37 (m, 3H), 7.25–7.18 (m, 5H), 7.16–7.12 (m, 1H), 6.81–6.78 (m, 1H), 6.59–6.57 (m, 1H), 6.49 (m_c_, 1H), 4.94 (s, 2H), 3.72 (s, 3H); ^13^C NMR (151 MHz, CDCl_3_) δ 160.0, 138.4, 138.3, 137.3, 134.7, 131.1 (2C), 130.7, 130.0, 129.0 (2C), 128.9, 128.8, 128.2 (2C), 126.6 (2C), 126.4, 119.3, 113.4, 112.7, 55.3, 48.8; ESI-MS (*m*/*z*) 341.2 (100, [M + H]^+^); anal. calcd for C_23_H_20_N_2_O (340.2) C 81.15, H 5.92, N 8.23; found C 81.12, H 5.92, N 8.31.

#### 1-Benzyl-2,4,5-trimethylimidazole^26^ (**5e**):

yellow oil; 164 mg (41%); ^1^H NMR (600 MHz, CDCl_3_) δ 7.32–7.29 (m, 2H), 7.27–7.23 (m, 1H), 6.94–6.92 (m, 2H), 4.96 (s, 2H), 2.28 (s, 3H), 2.14 (s, 3H), 2.00 (s, 3H); ^13^C NMR (151 MHz, CDCl_3_) δ 142.9, 136.8, 131.7, 129.0 (2C), 127.6, 125.8 (2C), 47.0, 13.4, 12.7, 9.0.

#### 1-Benzyl-2-methyl-4,5-diphenylimidazole^10^ (**5f**)

This compound was prepared following a general literature protocol^10^ in a 4.0 mmol scale; the crude product **5f** was isolated by preparative thin-layer chromatography (SiO_2_, CH_2_Cl_2_/MeOH, 98:2): yellow oil, 143 mg (11%); ^1^H NMR (600 MHz, CDCl_3_) δ 7.54–7.52 (m, 2H), 7.40–7.21 (m, 10H), 7.17–7.14 (m, 1H), 6.97–6.95 (m, 2H), 4.98 (s, 2H), 2.42 (s, 3H).

### General Procedures for Synthesis of Imidazole-2-thiones **7** and **8**

Method A: Imidazolium chloride **1** or **6** (0.19 mmol) and elemental sulfur (0.46 mmol) in a pyridine/Et_3_N mixture (1:1, 3.0 mL) were stirred at room temperature overnight. The solvents were removed in vacuo, and the obtained residue was purified by PTLC (using CH_2_Cl_2_ as an eluent) to give products isolated as solid materials.

Method B: Imidazolium salt **1** or **6** (0.3 mmol), solid Cs_2_CO_3_ (0.45 mmol), elemental sulfur (S_8_, 0.75 mmol), and zircon grinding balls were placed in a mechanochemical reactor, and the mixture was ground for 60 min. The resulting crude mixture was treated with CH_2_Cl_2_ (10 mL), and the solids were filtered off and washed with three portions of CH_2_Cl_2_ (5 mL each). After the solvent was removed under reduced pressure the crude product was purified by PTLC using CH_2_Cl_2_ as an eluent.

#### 1,3-Dibenzyl-4,5-dimethylimidazole-2-thione (**7a**)

Method A, 20 mg (34%), Method B, 89 mg (97%): colorless solid; mp 183–185 °C; IR (neat) ν 1442, 1401, 1353, 1230, 1003 cm^–1^; ^1^H NMR (600 MHz, CDCl_3_) δ 7.33–7.25 (m, 10H), 5.44 (s, 4H), 1.94 (s, 6H); ^13^C NMR (151 MHz, CDCl_3_) δ 162.8, 136.6, 128.8 (4C), 127.6 (2C), 127.1 (4C), 121.5 (2C), 48.9 (2C), 9.4 (2C); HRMS (ESI) *m*/*z* [M + H]^+^ calcd for C_19_H_21_N_2_S 309.1425, found 309.1427.

#### 1-(3′-Methoxybenzyl)-3-benzyl-4,5-dimethylimidazole-2-thione (**7c**)

Method A, 34 mg (53%), Method B, 74 mg (73%): colorless hygroscopic semisolid; IR (neat) ν 1606, 1495, 1431, 1405, 1263, 1230, 1144, 1040, 1003 cm^–1^; ^1^H NMR (600 MHz, CDCl_3_) δ 7.32–7.22 (m, 6H), 6.87–6.79 (m, 3H), 5.44 (s, 2H), 5.41 (s, 2H), 3.78 (s, 3H), 1.96 (s, 3H), 1.94 (s, 3H); ^13^C NMR (151 MHz, CDCl_3_) δ 162.9, 160.0, 138.3, 136.7, 129.8, 128.8 (2C), 127.6, 127.0 (2C), 121.54, 121.45, 119.3, 113.1, 112.6, 55.3, 49.0, 48.9, 9.37, 9.36; HRMS (ESI) *m*/*z* [M + H]^+^ calcd for C_20_H_23_N_2_OS 339.1531, found 339.1527.

#### 1,3-Dibenzyl-4,5-diphenylimidazole-2-thione (**8a**)

Method A, 39 mg (48%), Method B, 95 mg (73%): colorless solid; mp 180–182 °C; IR (neat) ν 1495, 1446, 1402, 1349, 1234, 1080, 1021, 950 cm^–1^; ^1^H NMR (600 MHz, CDCl_3_) δ 7.28–2.21 (m, 8H), 7.19–7.16 (m, 4H), 7.09–7.06 (m, 4H), 6.99–6.96 (m, 4H), 5.43 (s, 4H); ^13^C NMR (151 MHz, CDCl_3_) δ 164.2, 136.9 (2C), 130.8 (4C), 128.9 (2C), 128.51 (4C), 128.49 (6C), 128.1 (2C), 127.53 (4C), 127.48 (2C), 49.4 (2C); HRMS (ESI) *m*/*z* [M + H]^+^ calcd for C_29_H_24_N_2_S 433.1738, found 433.1739.

#### 1-(3′-Methoxybenzyl)-3-benzyl-4,5-diphenylimidazole-2-thione (**8c**)

Method A, 64 mg (73%), Method B, 107 mg (77%): colorless solid; mp 52–55 °C; IR (neat) ν 1603, 1495, 1435, 1405, 1349, 1230, 1047 cm^–1^; ^1^H NMR (600 MHz, CDCl_3_) δ 7.28–7.12 (m, 10H), 7.10–7.07 (m, 2H), 7.01–6.96 (m, 4H), 6.78–6.75 (m, 1H), 6.69–6.66 (m, 2H), 5.43 (s, 2H), 5.41 (s, 2H), 3.71 (s, 3H); ^13^C NMR (151 MHz, CDCl_3_) δ 164.2, 159.7, 138.4, 136.9, 130.79 (2C), 130.76 (2C), 129.5, 128.9 (2C), 128.5 (4C), 128.44 (2C), 128.41 (2C), 128.07, 128.06, 127.45 (2C), 127.42, 119.8, 113.5, 112.6, 55.2, 49.4, 49.31; HRMS (ESI) *m*/*z* [M + H]^+^ calcd for C_30_H_27_N_2_OS 463.1844, found 463.1837.

### X-ray Crystallographic Data of **1c[PF**_**6**_**]**

Pale yellow crystals of compound **1c[PF**_**6**_**]** were obtained from a CH_2_Cl_2_/*i*-Pr_2_O mixture by slow evaporation of the solvents at room temperature. A suitable crystal was measured on an XtaLAB Synergy, Dualflex, Pilatus 300 K diffractometer. The crystal was mounted in inert oil on nylon loops and kept at 100.00(10) K during data collection. Measurements for compound **1c**[**PF**_**6**_] were performed using mirror-focused Cu Kα radiation, λ = 1.541 84 Å. Absorption corrections were implemented on the basis of multiscans. Using Olex2,^27^ the structure was solved with the XT^28^ structure solution program using intrinsic phasing and refined with the XL^29^ refinement package using least squares minimization. Hydrogen atoms were included using rigid methyl groups or a riding model starting from calculated positions. Complete data have been deposited with the Cambridge Crystallographic Data Centre under the number CCDC-2059690. Copies of the data can be obtained free of charge from www.ccdc.cam.ac.uk/structures/.

C_20_H_23_F_6_N_2_OP, M = 452.37: triclinic, space group *P*1̅ (no. 2), *a* = 9.8930(3) Å, *b* = 11.1547(4) Å, *c* = 11.1640(4) Å, α = 60.849(4)°, β = 84.409(3)°, γ = 71.453(3)°, *V* = 1017.60(7) Å^3^, *Z* = 2, *T* = 100.00(10) K, μ(Cu Kα) = 1.834 mm^–1^, *D*_c_ = 1.476 g cm^–3^, 23 177 reflections measured (9.092° ≤ 2θ ≤ 157.666°), 4125 unique (*R*_int_ = 0.0370, *R*_sigma_ = 0.0187), which were used in all calculations. The final *R*_1_ was 0.0319 (*I* > 2σ(*I*)) and *wR*_2_ was 0.0824 (all data).

### Cell Culture and Treatment

Human promyelocytic leukemia (HL-60) and human breast cancer adenocarcinoma (MCF-7) cell lines were obtained from the European Collection of Cell Cultures, while HUVECs were purchased from the American Type Culture Collection. HL-60 cells were cultured in RPMI 1640 plus GlutaMax I medium (Gibco/Life Technologies, Carlsbad, CA, USA). MCF-7 cells were maintained in minimum essential medium Eagle (Sigma-Aldrich, St. Louis, MO, USA) supplemented with 2 mM glutamine and Men nonessential amino acid solution (Sigma-Aldrich). Both media were supplemented with 10% heat-inactivated fetal bovine serum (Biological Industries, Beit-Haemek, Israel) and antibiotics (100 U/mL penicillin and 100 μg/mL streptomycin) (Sigma-Aldrich). HUVEC cells were grown in EGM-2 endothelial medium BulletKit (Lonza, Basel, Switzerland). Cells were maintained at 37 °C in a 5% CO_2_ atmosphere and were grown until 80% confluent.

The tested compounds were dissolved in sterile dimethyl sulfoxide (DMSO) and further diluted with culture medium. The final concentration of DMSO in cell cultures was less than 0.1% v/v. Controls without and with 0.1% DMSO were performed in each experiment. At the used concentration DMSO had no effect on the observed parameters.

### Cytotoxicity Determination (MTT Assay)

The MTT assay was performed according to the known procedure.^30^ The cells were incubated with the analogues for 48 h. The absorbance of the blue formazan product was measured at 560 nm using a FlexStation 3 multi-mode microplate reader (Molecular Devices, LLC, CA, USA) and compared with control (untreated cells). All experiments were performed in triplicate.

## Supporting Information Available

X-ray crystallographic data of **1c[PF**_**6**_**]** (CIF)
NMR (^1^H and ^13^C) of compounds **1**–**8** (PDF)
Raw NMR data (fid files) for compounds **1**–**8** (ZIP)

## Supplementary Material

np1c00797_si_001.cif
