# Supplementary material for: Synthesis and Cytotoxic Activity of Lepidilines A–D: Comparison with Some 4,5-Diphenyl Analogues and Related Imidazole-2-thiones
Source: J Nat Prod. 2021 Nov 22;84(12):3071–9. doi: 10.1021/acs.jnatprod.1c00797 (PMC8713287; doi:10.1021/acs.jnatprod.1c00797)
Supplement: Supplementary file 2 — np1c00797_si_002.pdf [file np1c00797_si_002.pdf]

Supporting Information

For

Synthesis and cytotoxic activity of lepidilines A-D; comparison with some 4,5-diphenyl analogues and related imidazole-2-thiones

Grzegorz Mlostoń,[a,*] Mateusz Kowalczyk,[a,b] Małgorzata Celeda,[a] Katarzyna Gach-Janczak,[c] Anna Janecka,[c] Marcin Jasiński[a,*]

[a] *Faculty of Chemistry, University of Lodz, Tamka 12, 91403 Łódź, Poland*
grzegorz.mloston@chemia.uni.lodz.pl; mjasinski@uni.lodz.pl

[b] *The Bio-Med-Chem Doctoral School of the University of Lodz and Lodz Institutes of the Polish Academy of Sciences, Faculty of Biology and Environmental Protection, University of Lodz, Banacha 12/16, 90237 Łódź, Poland*

[c] *Department of Biomolecular Chemistry, Medical University of Lodz, Mazowiecka 6/8, 92215 Łódź, Poland*

Copies of ^{1}H and ^{13}C NMR spectra **S2**

Imidazolium salts **1** and **6** S2

Imidazole *N*-oxides **2** S16

Trimer of formaldimine **4b** S19

Imidazoles **5** S20

Imidazole-2-thiones **7** and **8** S23

Crystallographic analysis of 1c[PF$_6$] **S27**

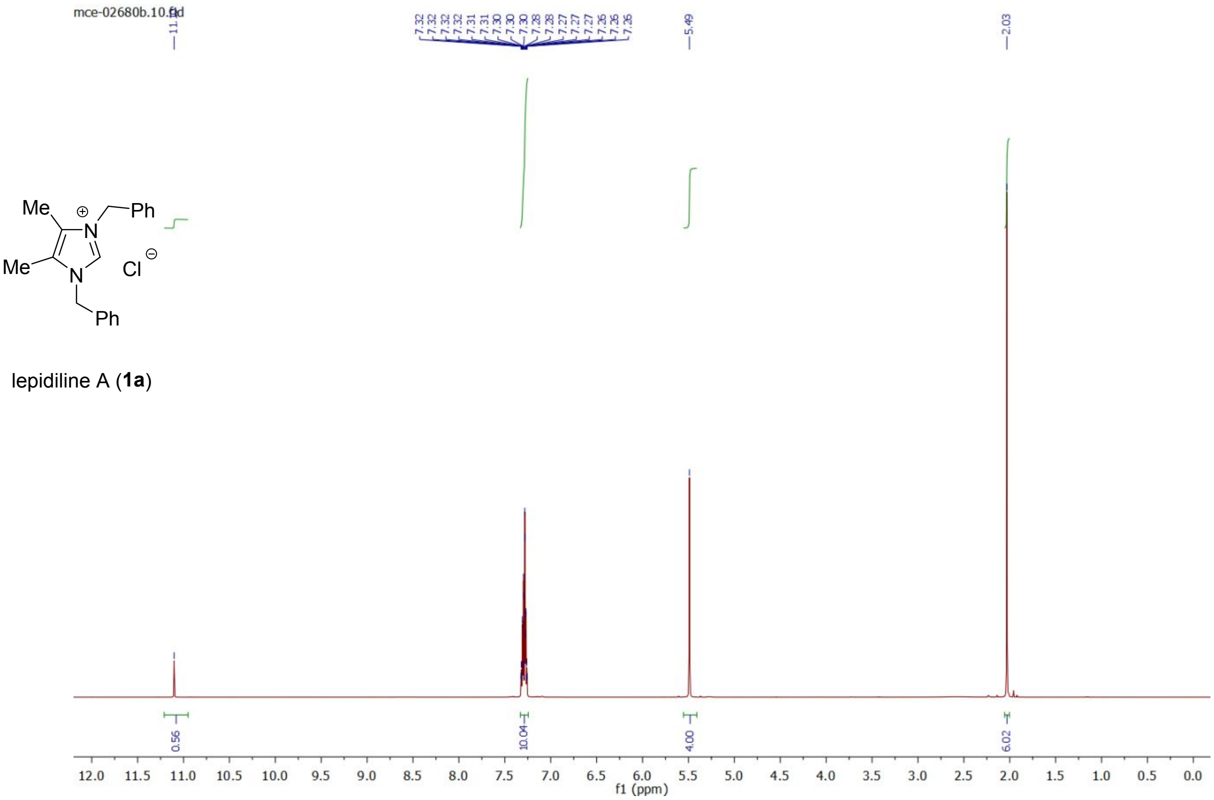

Fig. S1. The ^{1}H NMR (600 MHz, $CDCl_3$) spectrum for compound **1a**.

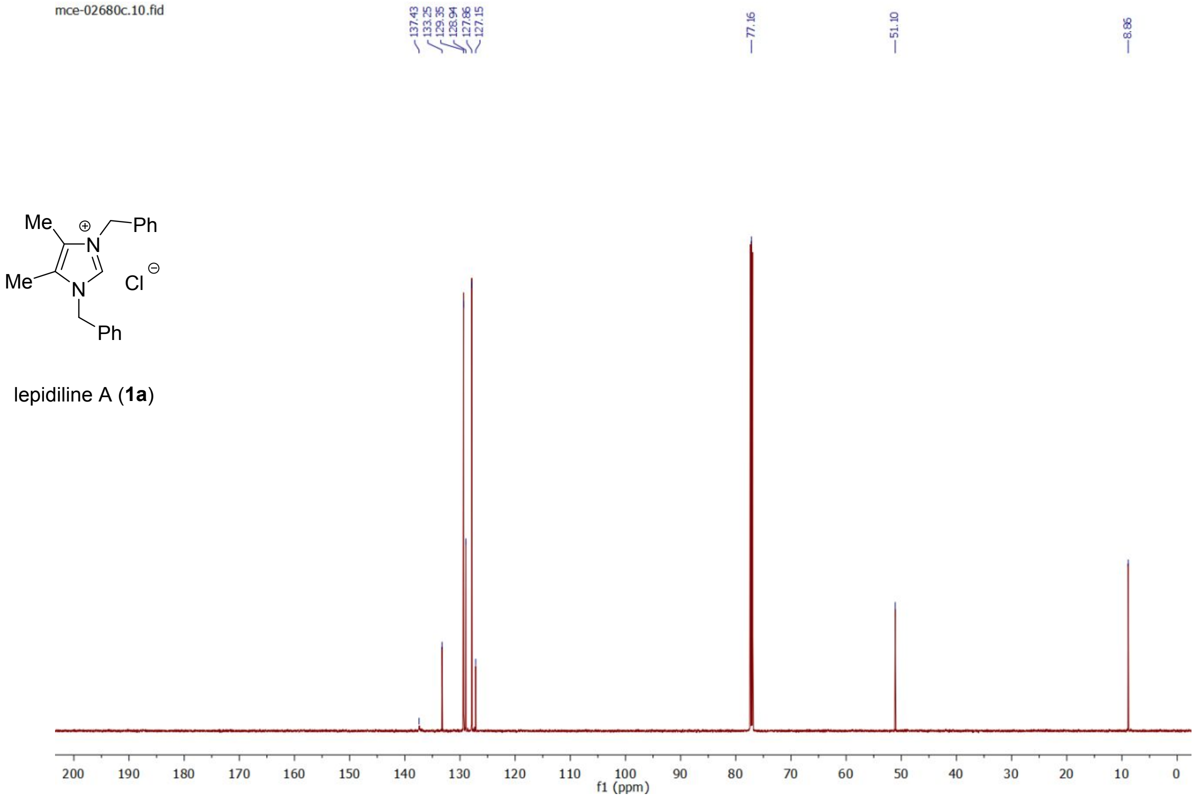

Fig. S2. The ^{13}C NMR (151 MHz, $CDCl_3$) spectrum for compound **1a**.

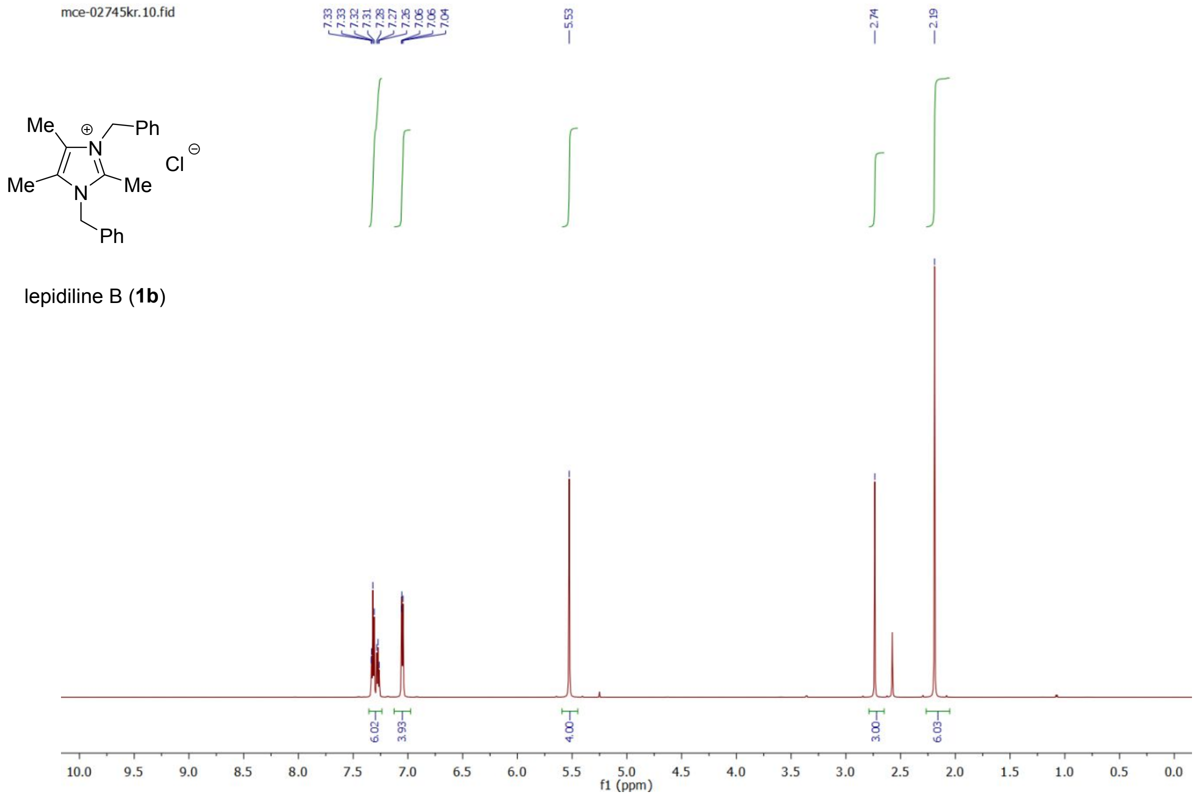

Fig. S3. The ^{1}H NMR (600 MHz, $CDCl_3$) spectrum for compound **1b**.

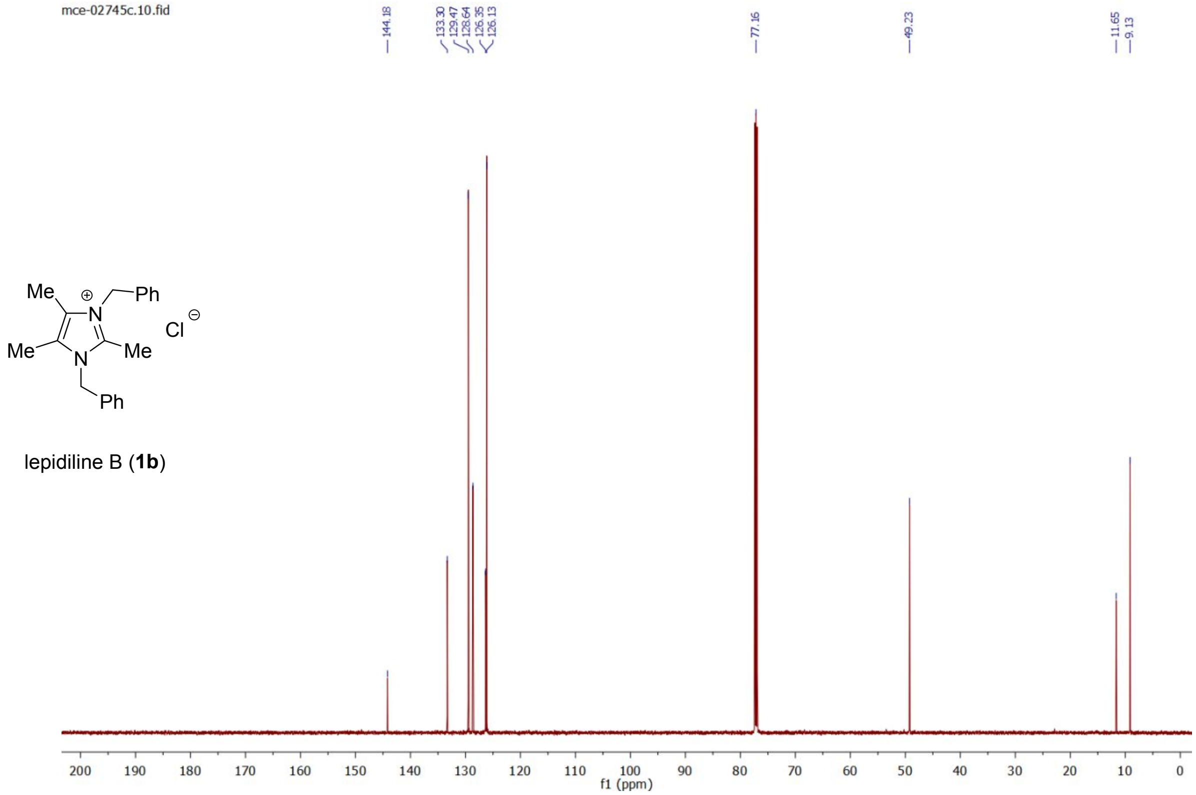

Fig. S4. The ^{13}C NMR (151 MHz, $CDCl_3$) spectrum for compound **1b**.

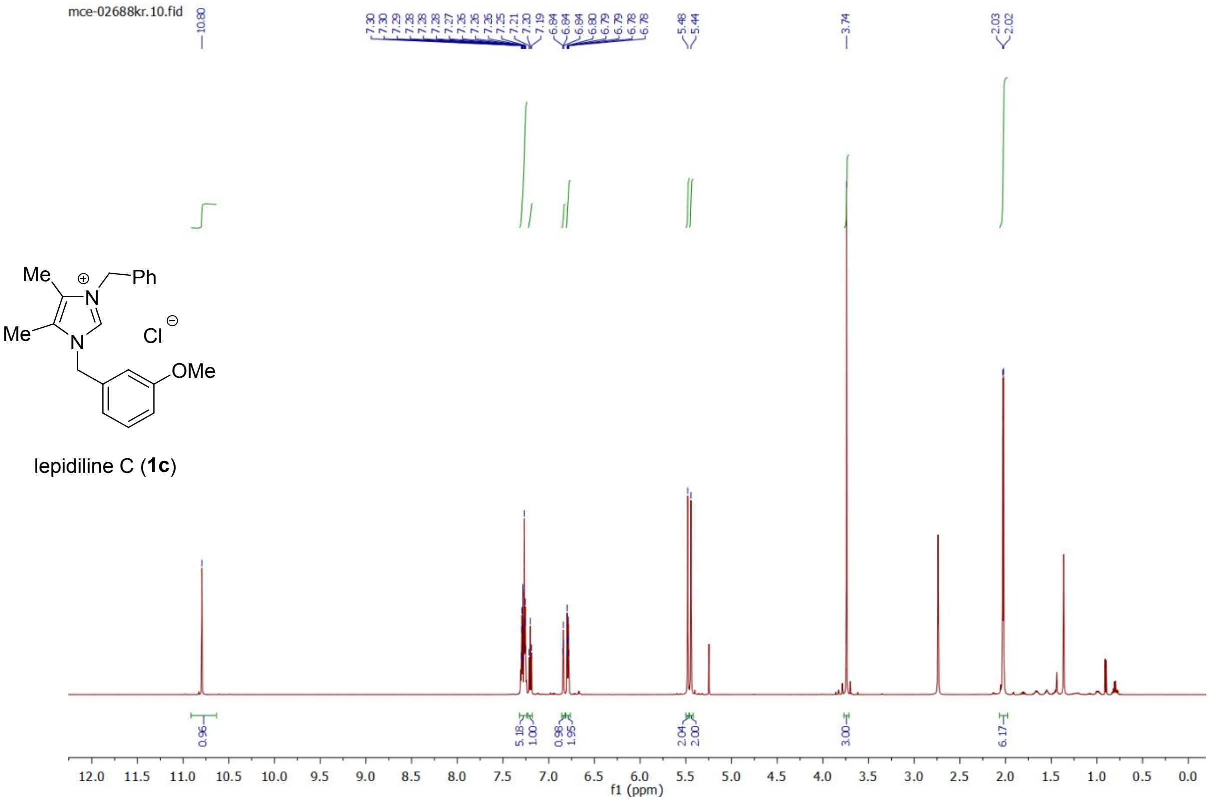

Fig. S5. The ^{1}H NMR (600 MHz, $CDCl_3$) spectrum for compound **1c**.

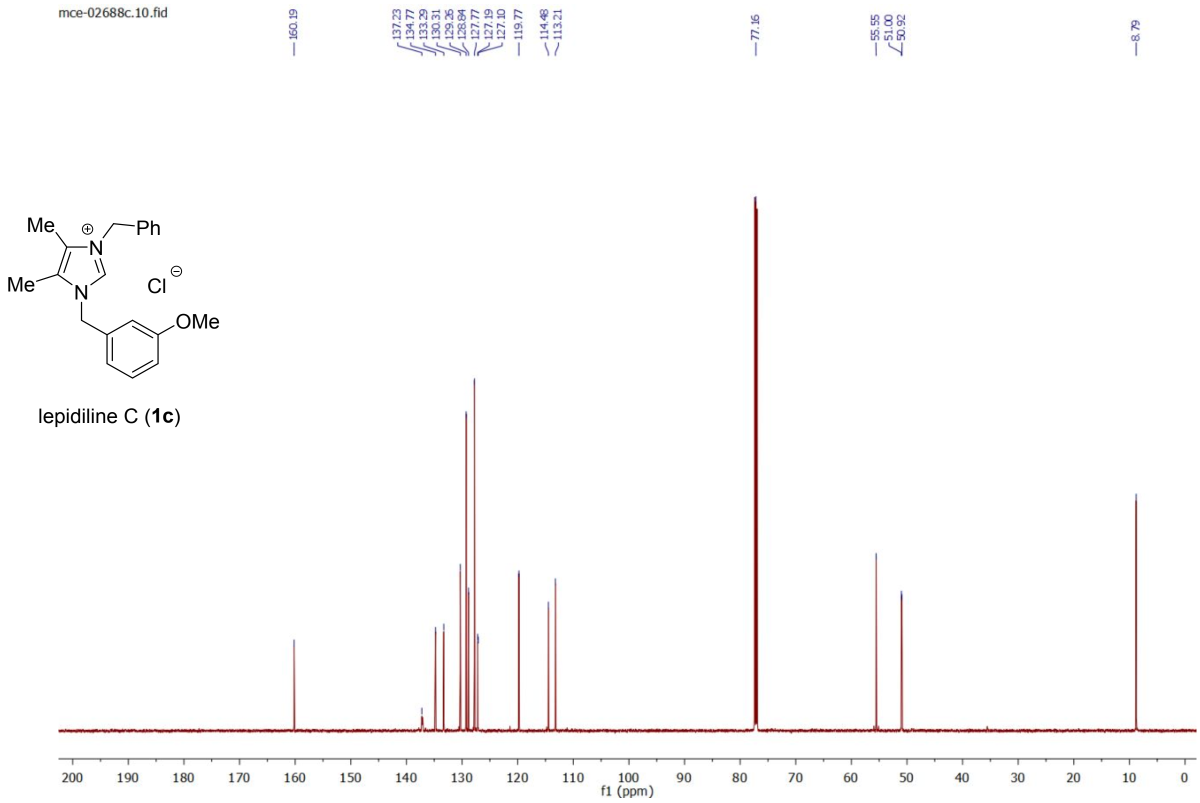

Fig. S6. The ^{13}C NMR (151 MHz, $CDCl_3$) spectrum for compound **1c**.

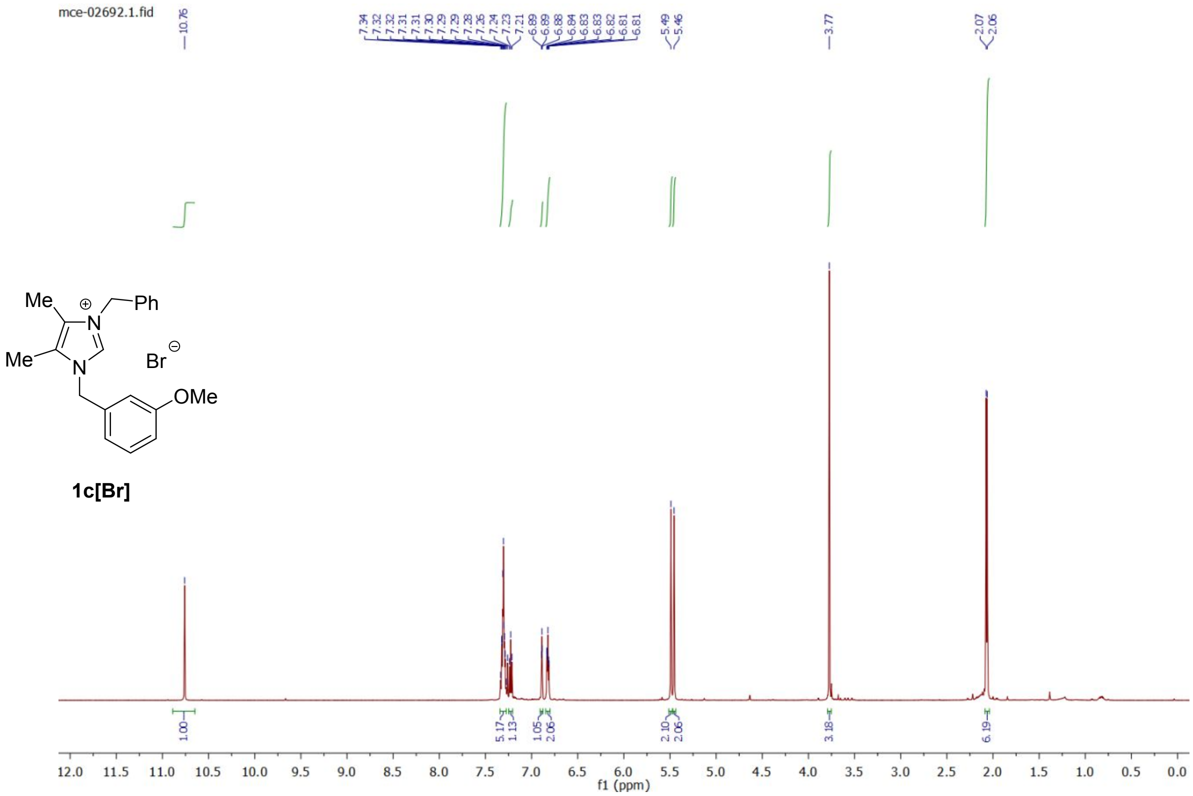

Fig. S7. The ^{1}H NMR (600 MHz, $CDCl_3$) spectrum for compound **1c[Br]**.

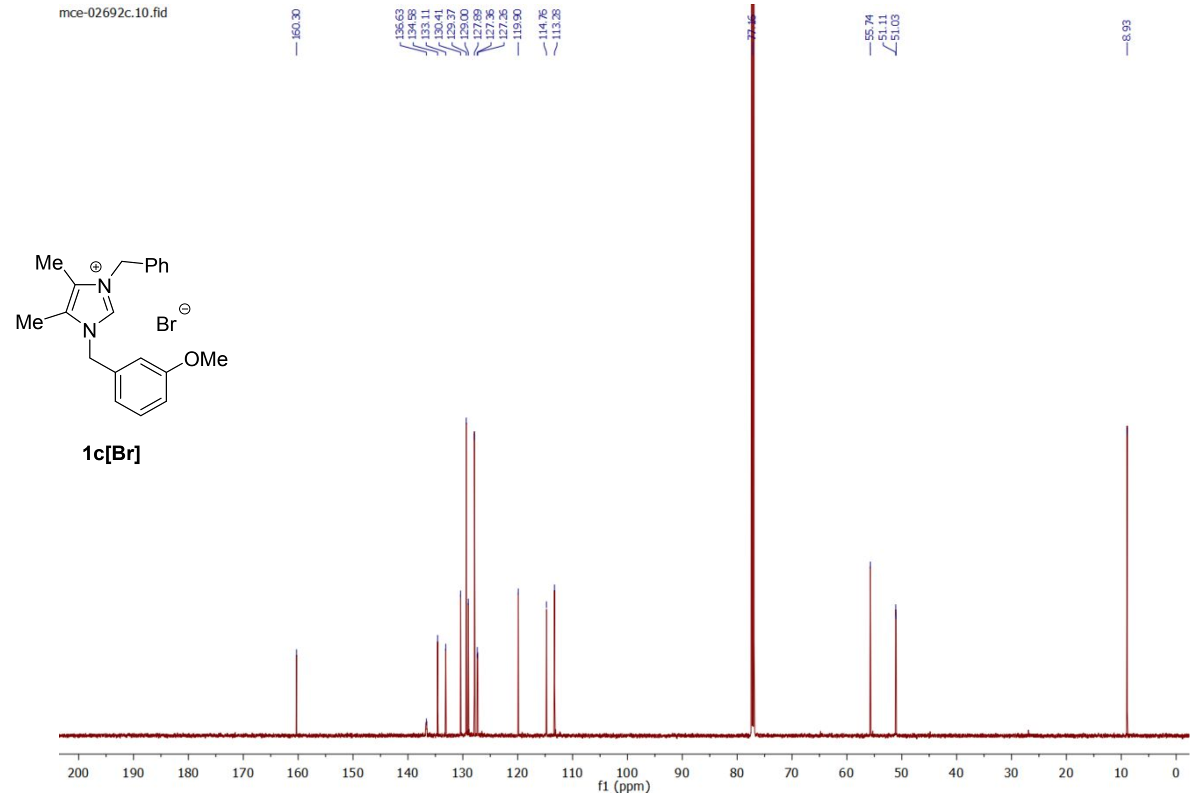

Fig. S8. The ^{13}C NMR (151 MHz, $CDCl_3$) spectrum for compound **1c[Br]**.

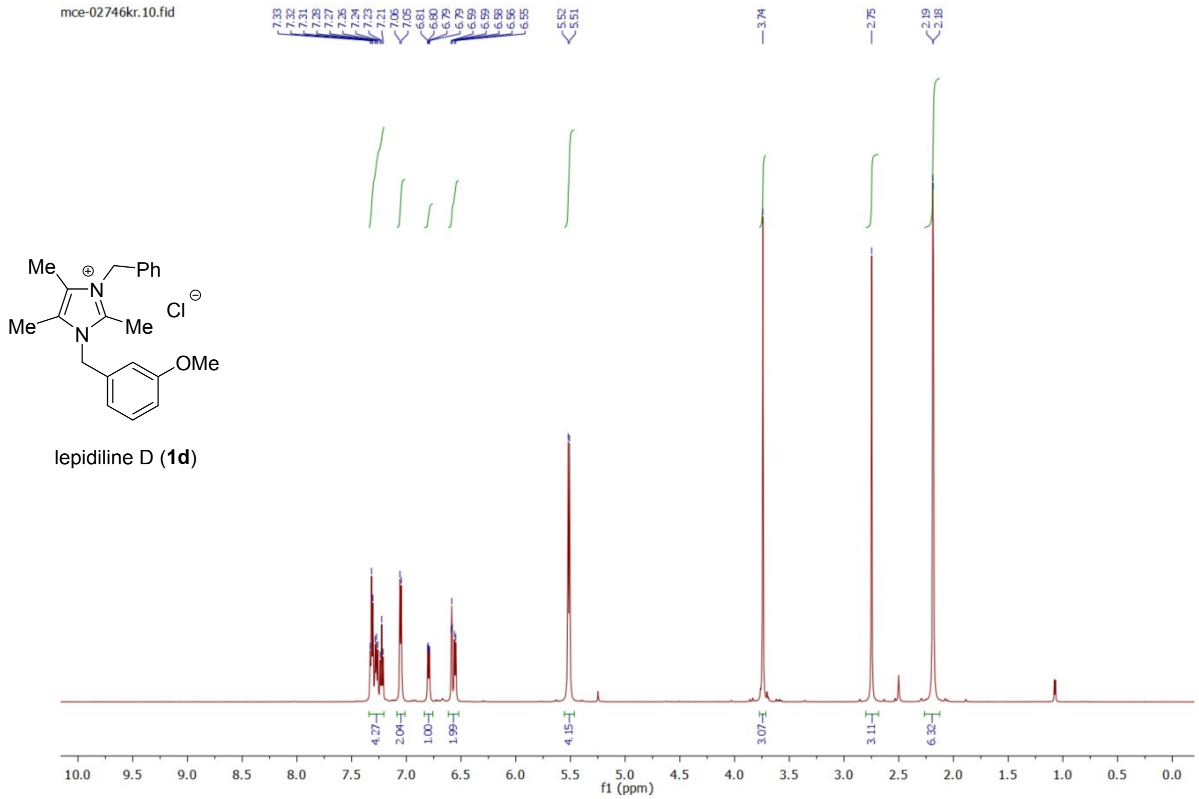

Fig. S9. The ^{1}H NMR (600 MHz, $CDCl_3$) spectrum for compound **1d**.

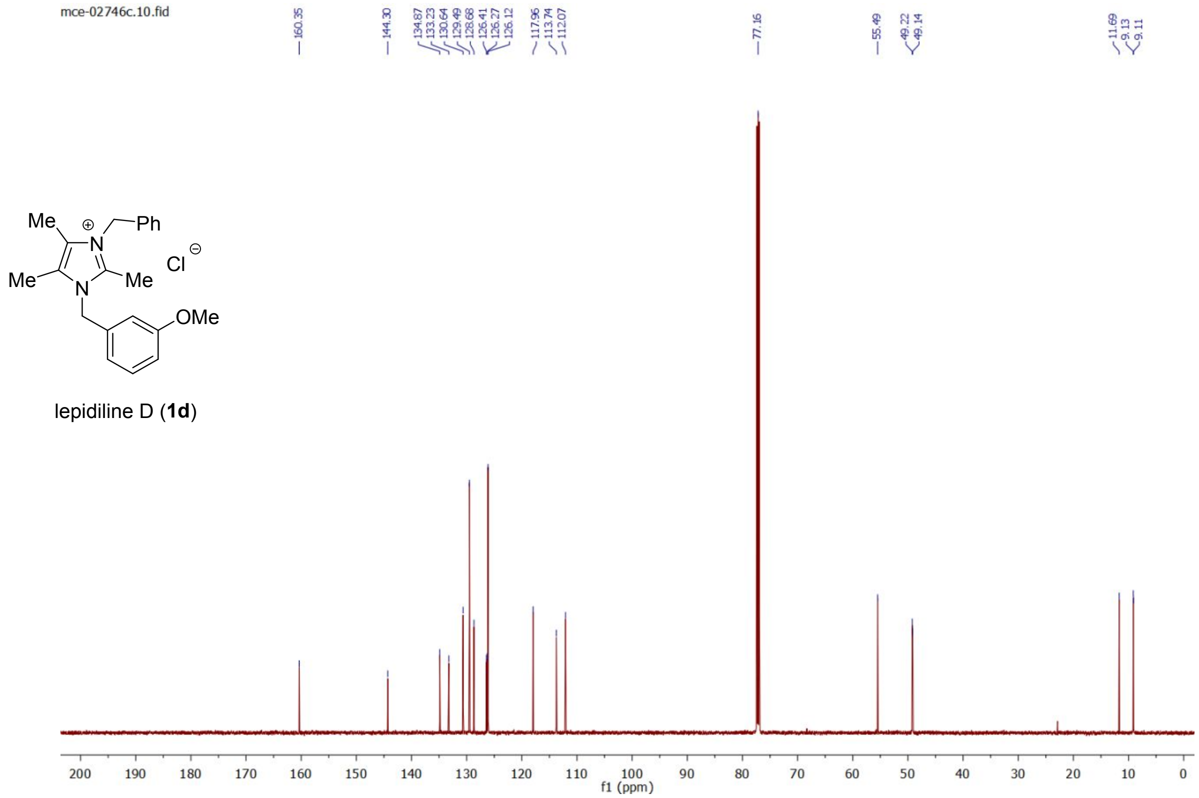

Fig. S10. The ^{13}C NMR (151 MHz, $CDCl_3$) spectrum for compound **1d**.

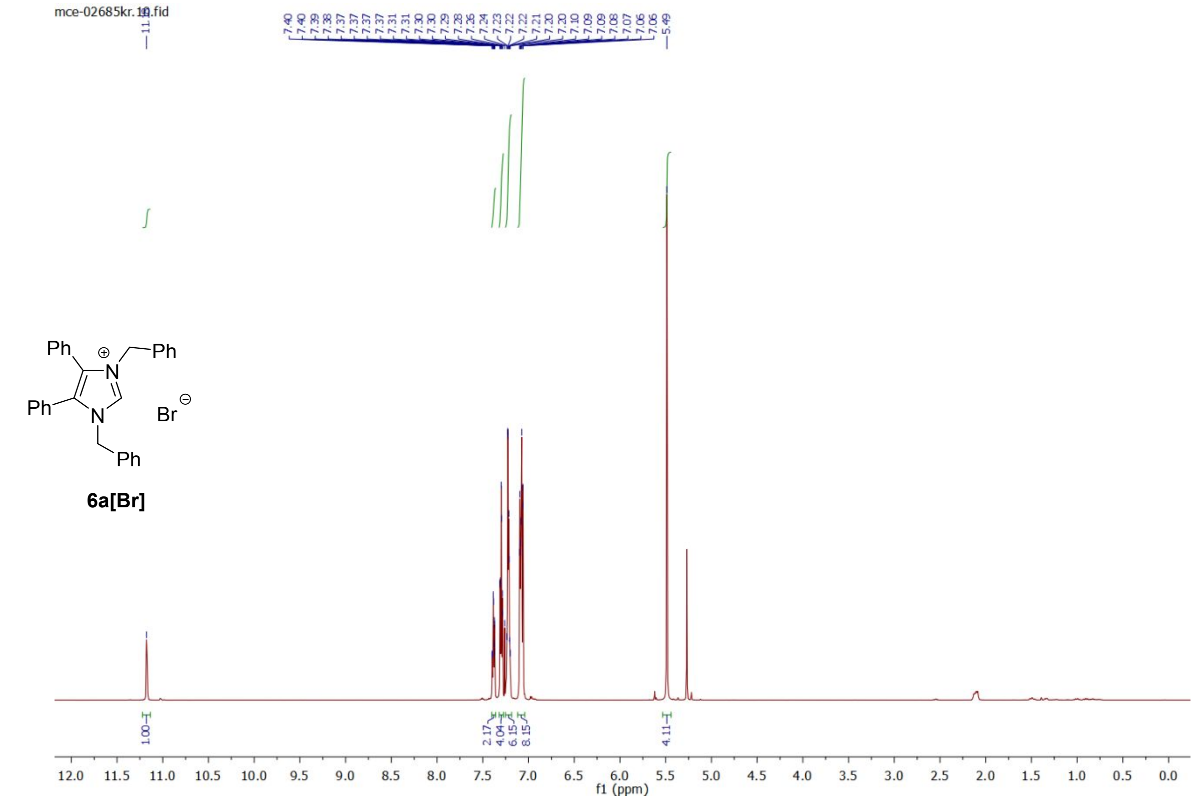

Fig. S11. The ^{1}H NMR (600 MHz, $CDCl_3$) spectrum for compound **6a[Br]**.

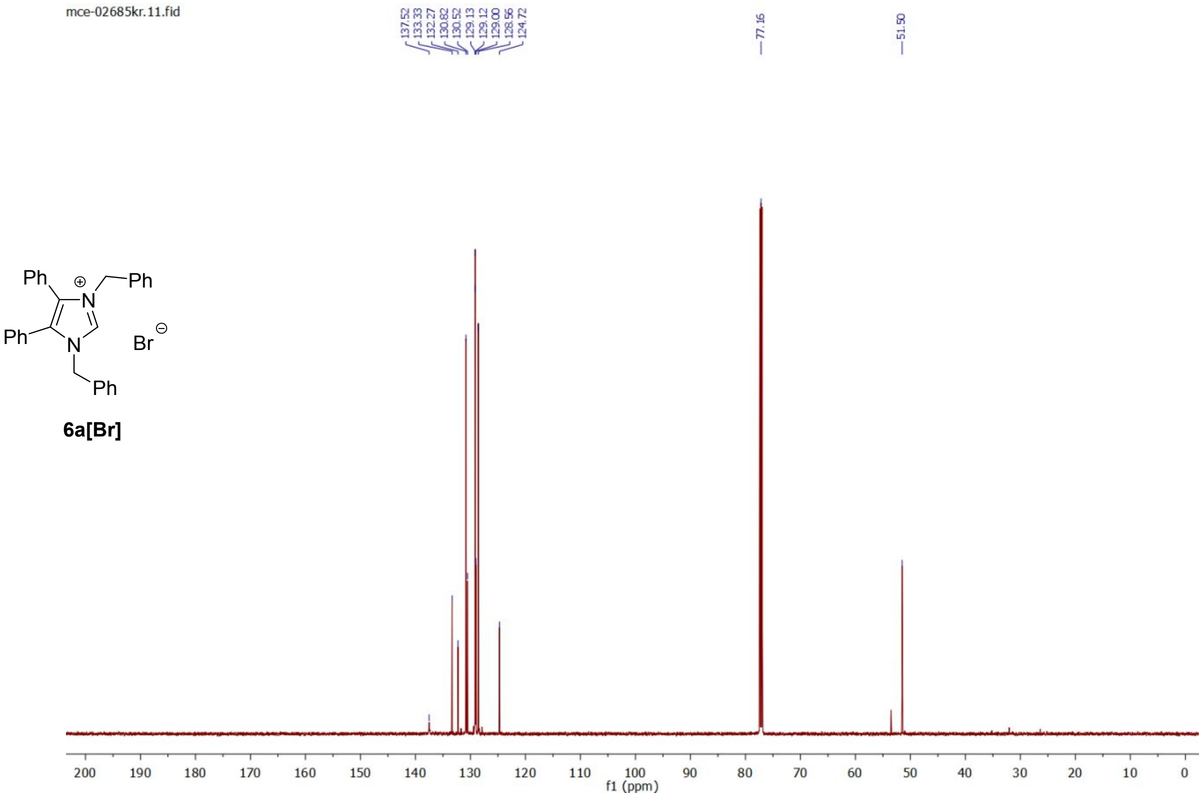

Fig. S12. The ^{13}C NMR (151 MHz, $CDCl_3$) spectrum for compound **6a[Br]**.

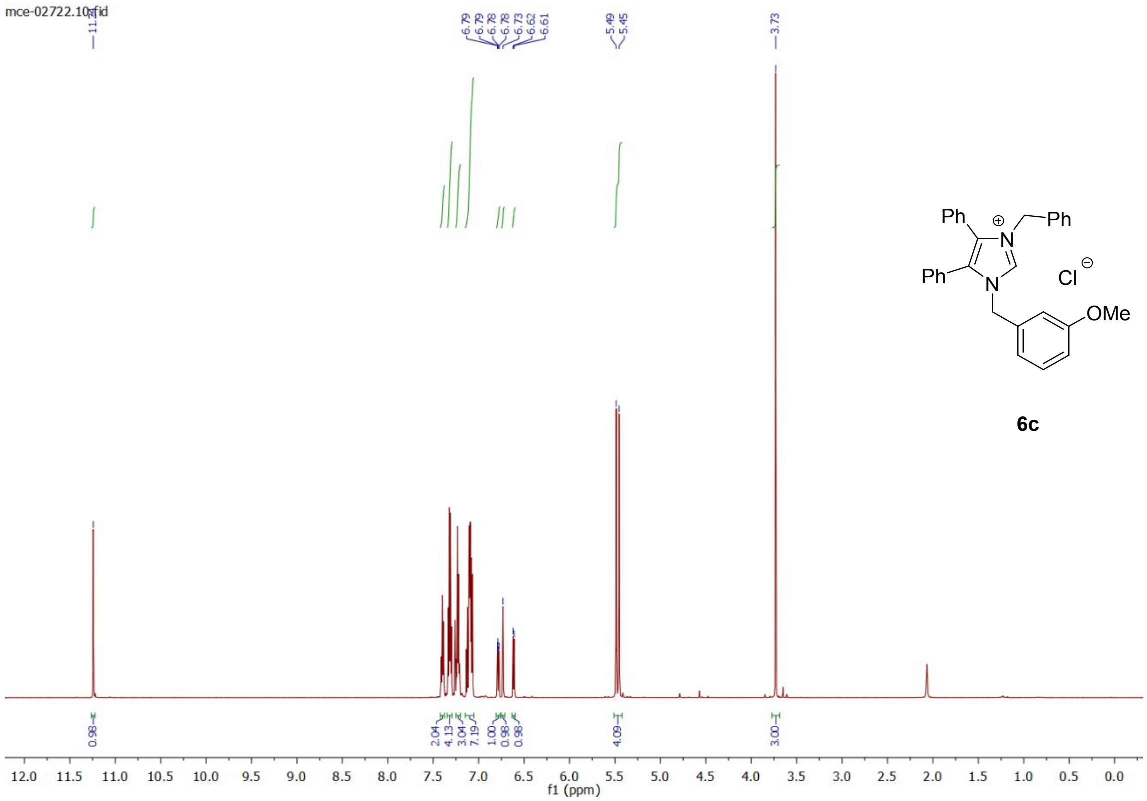

Fig. S13. The ^{1}H NMR (600 MHz, $CDCl_3$) spectrum for compound **6c**.

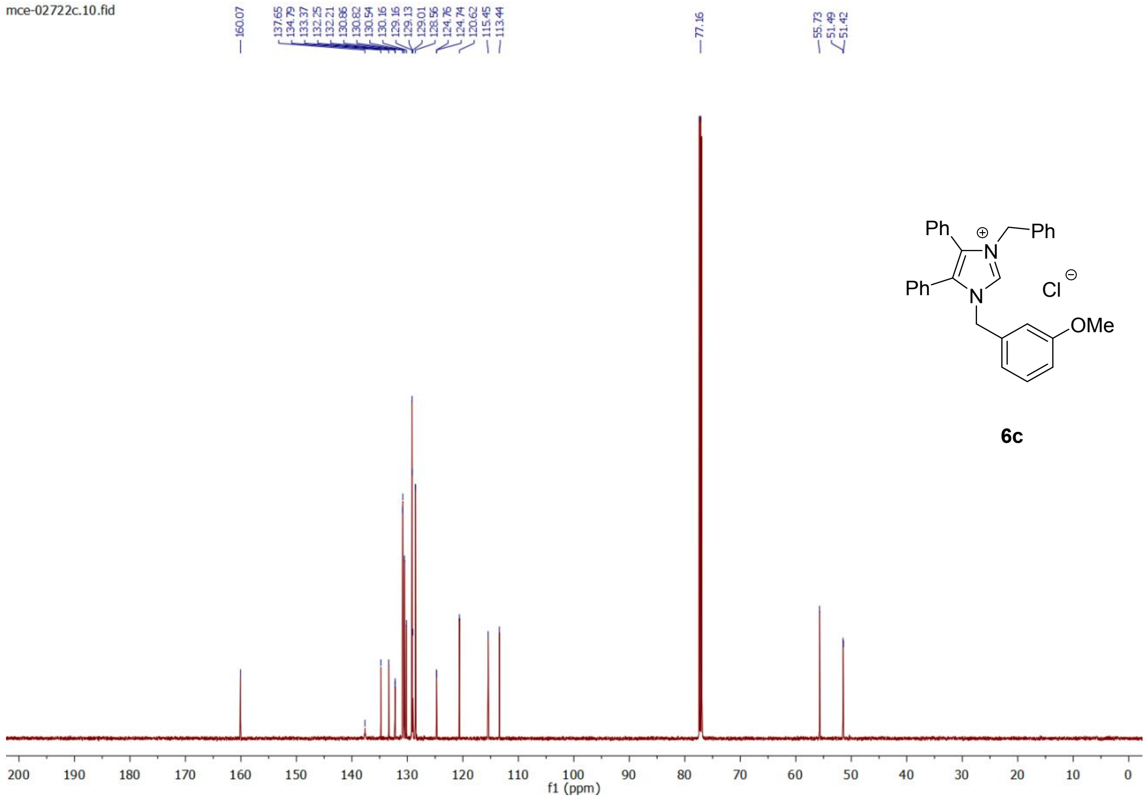

Fig. S14. The ^{13}C NMR (151 MHz, $CDCl_3$) spectrum for compound **6c**.

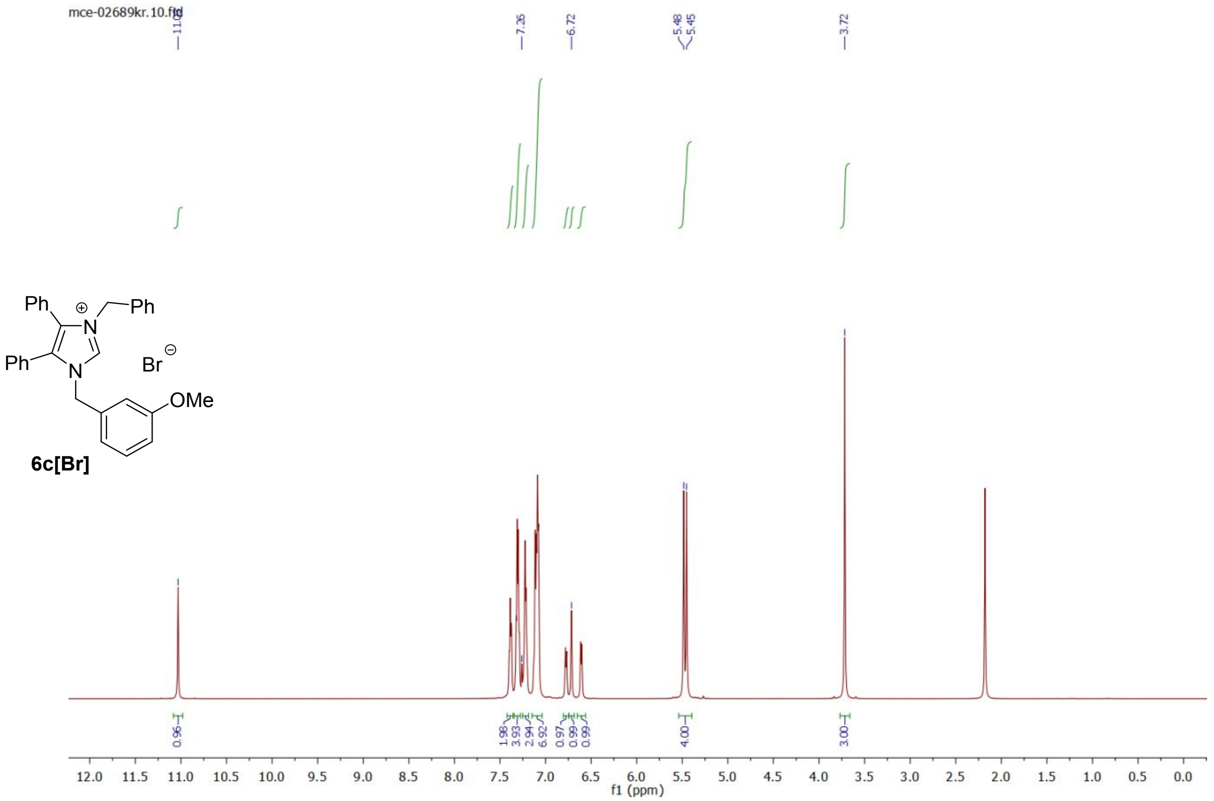

Fig. S15. The ^{1}H NMR (600 MHz, $CDCl_3$) spectrum for compound **6c[Br]**.

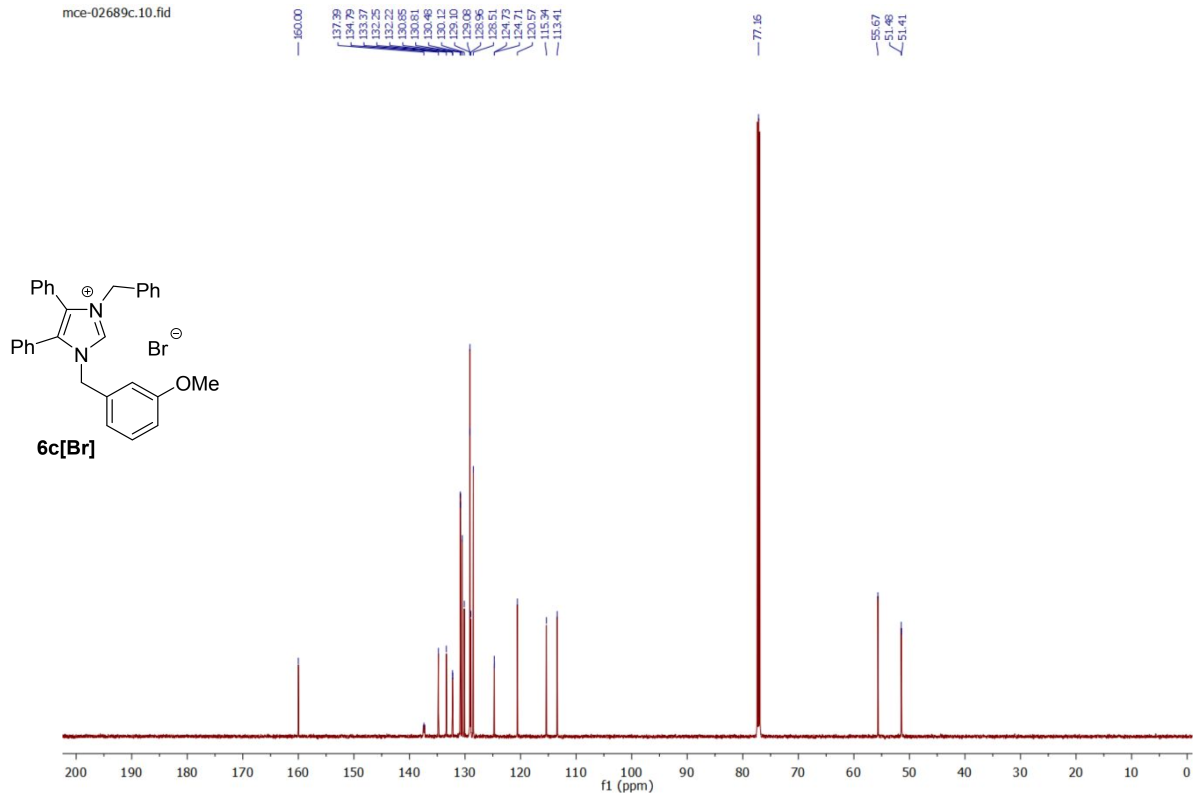

Fig. S16. The ^{13}C NMR (151 MHz, $CDCl_3$) spectrum for compound **6c[Br]**.

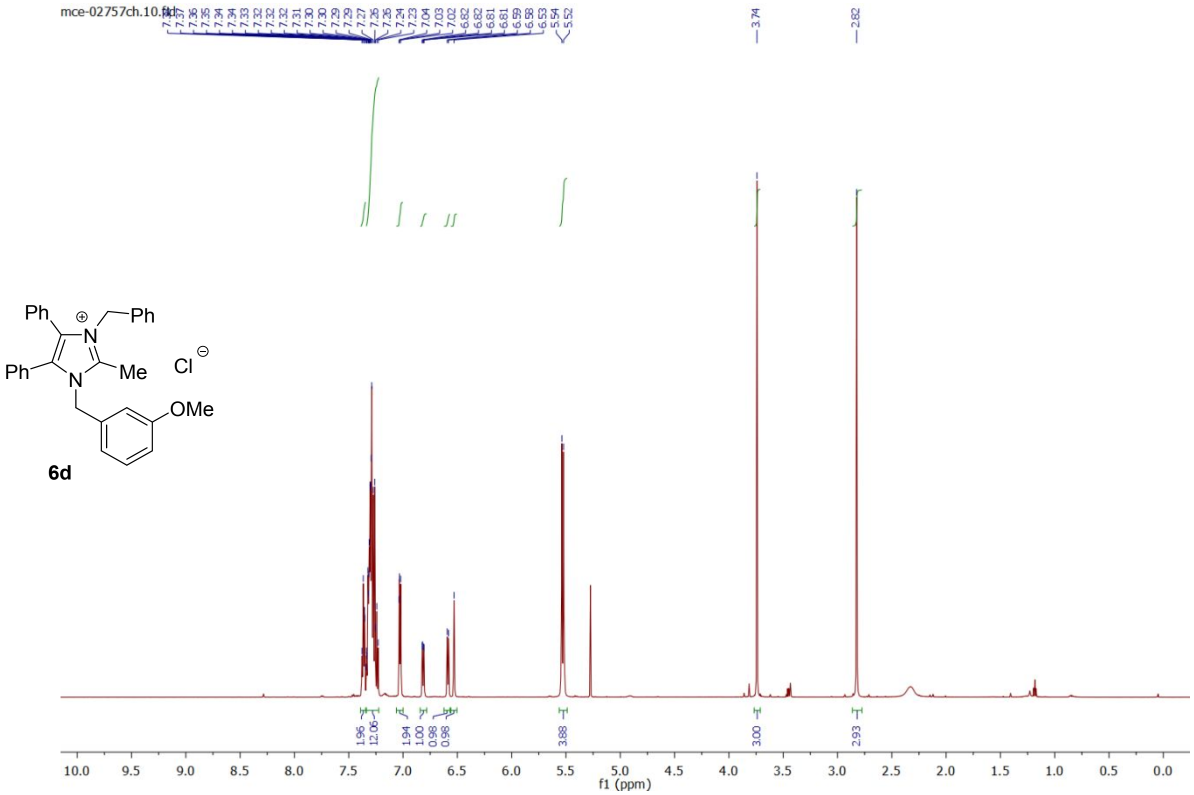

Fig. S17. The ^{1}H NMR (600 MHz, $CDCl_3$) spectrum for compound **6d**.

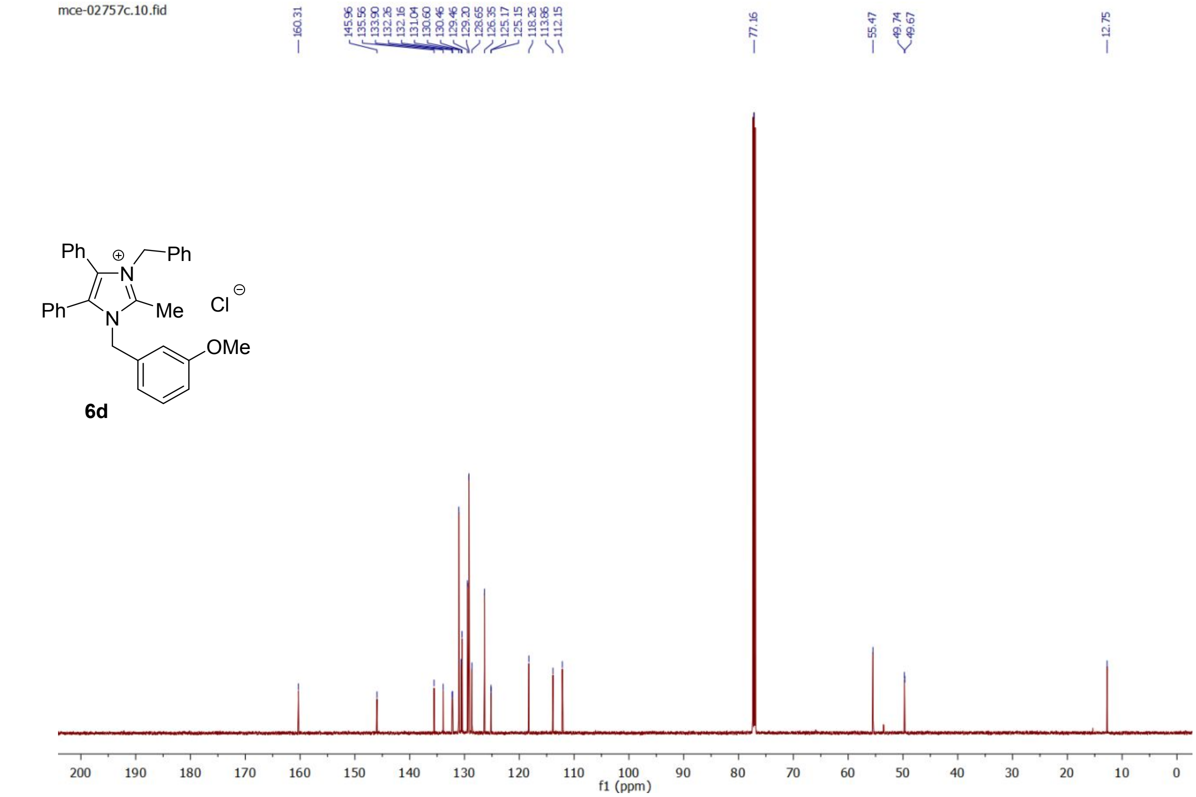

Fig. S18. The ^{13}C NMR (151 MHz, $CDCl_3$) spectrum for compound **6d**.

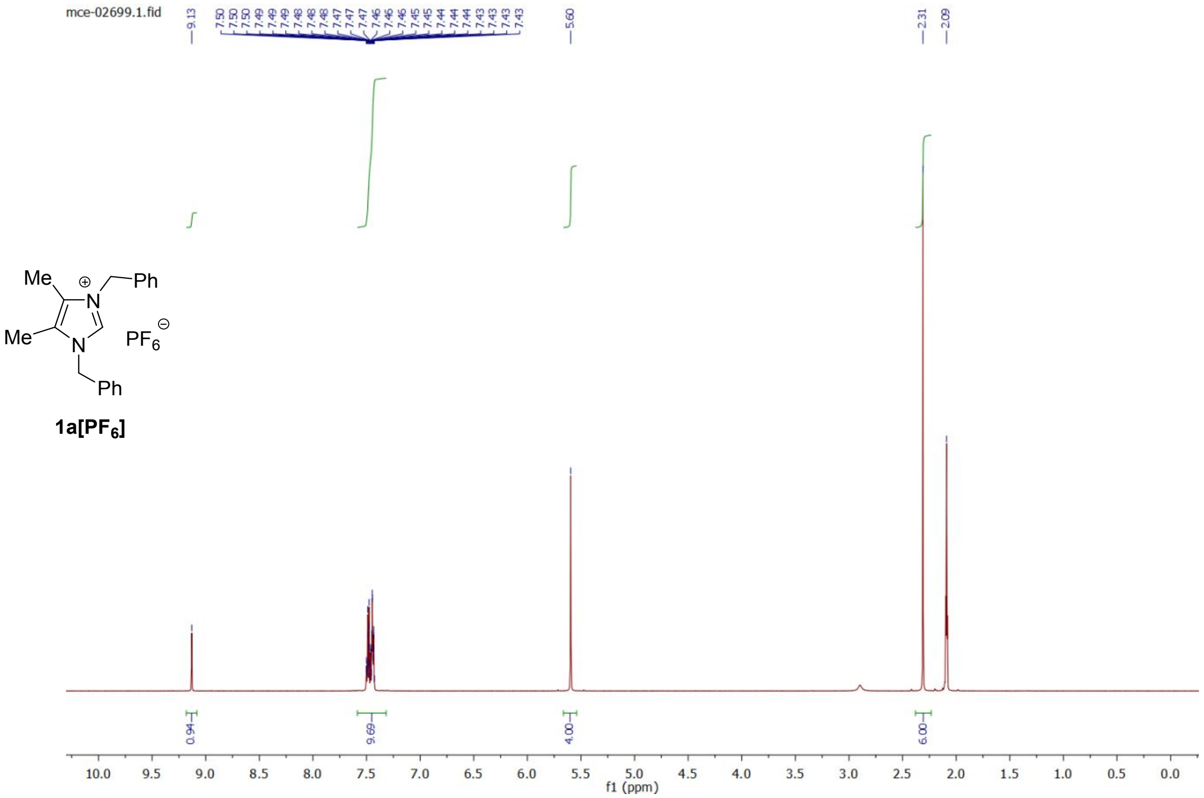

Fig. S19. The ^{1}H NMR (600 MHz, acetone-d_6) spectrum for compound **1a[PF$_6$]**.

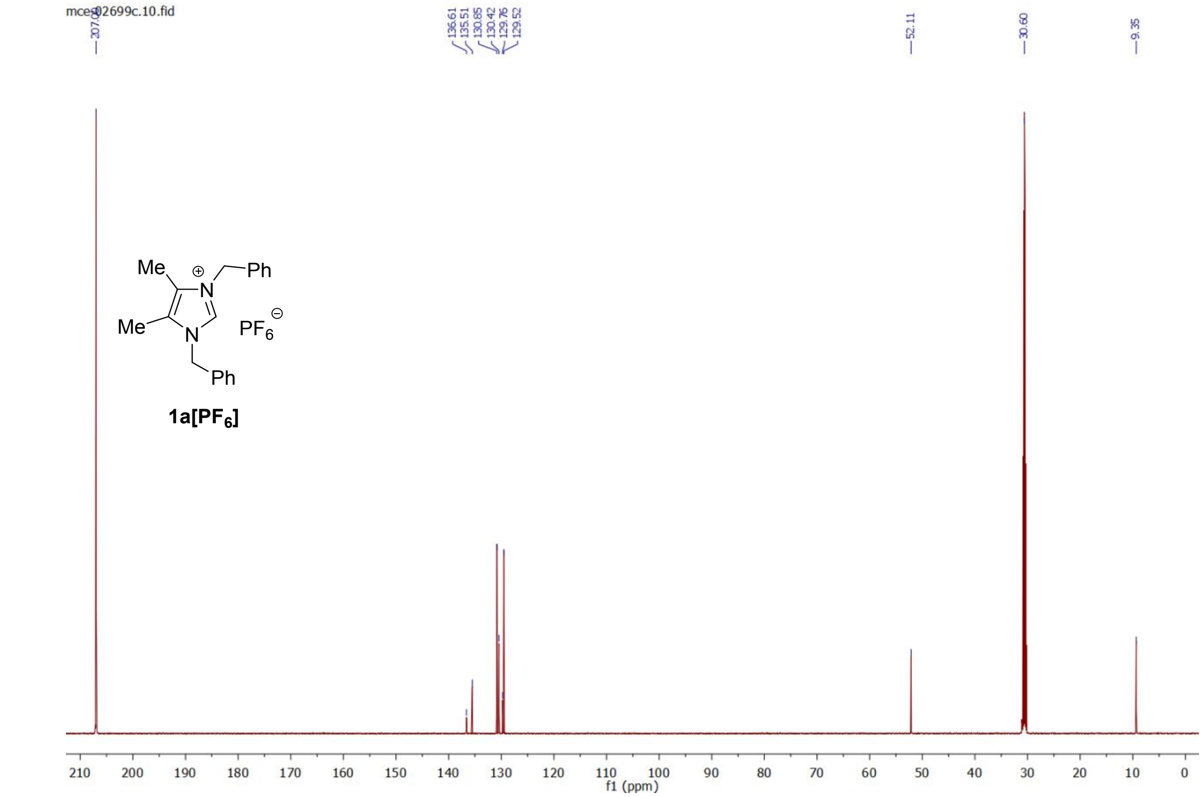

Fig. S20. The ^{13}C NMR (151 MHz, acetone-d_6) spectrum for compound **1a[PF$_6$]**.

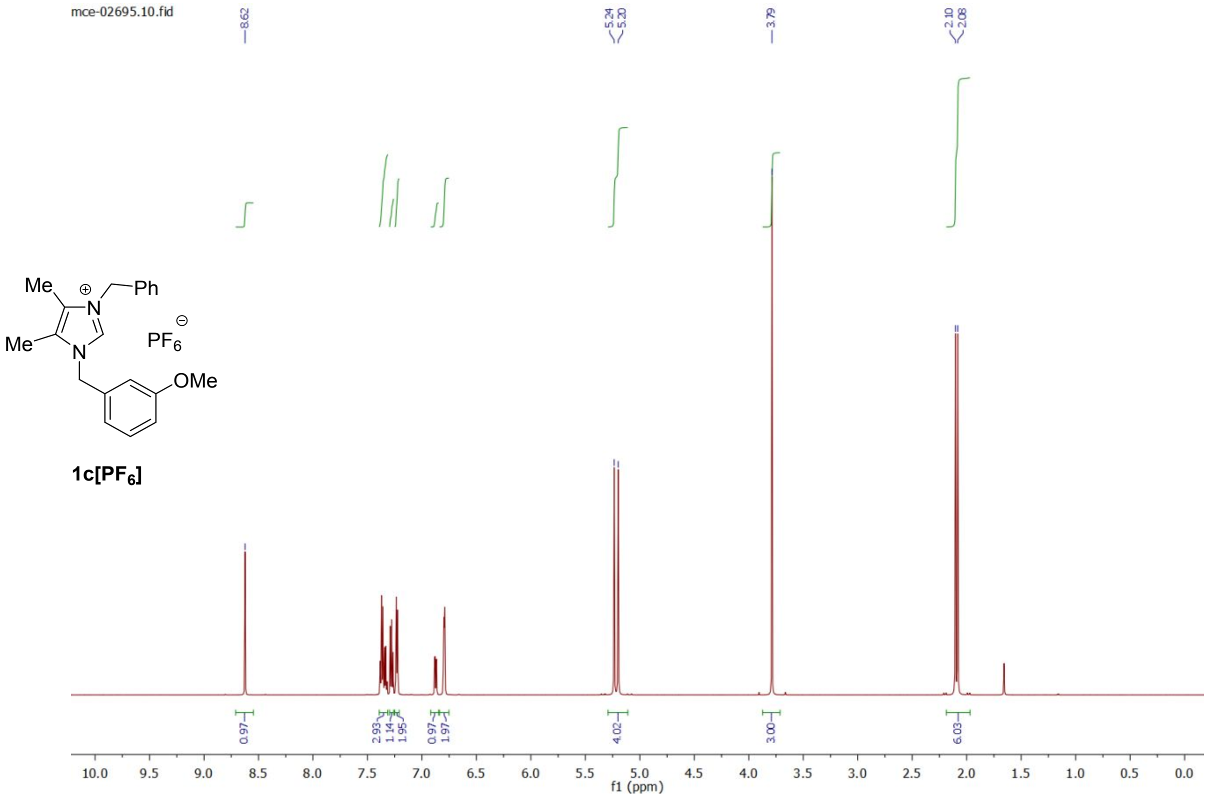

Fig. S21. The ^{1}H NMR (600 MHz, $CDCl_3$) spectrum for compound **1c[PF$_6$]**.

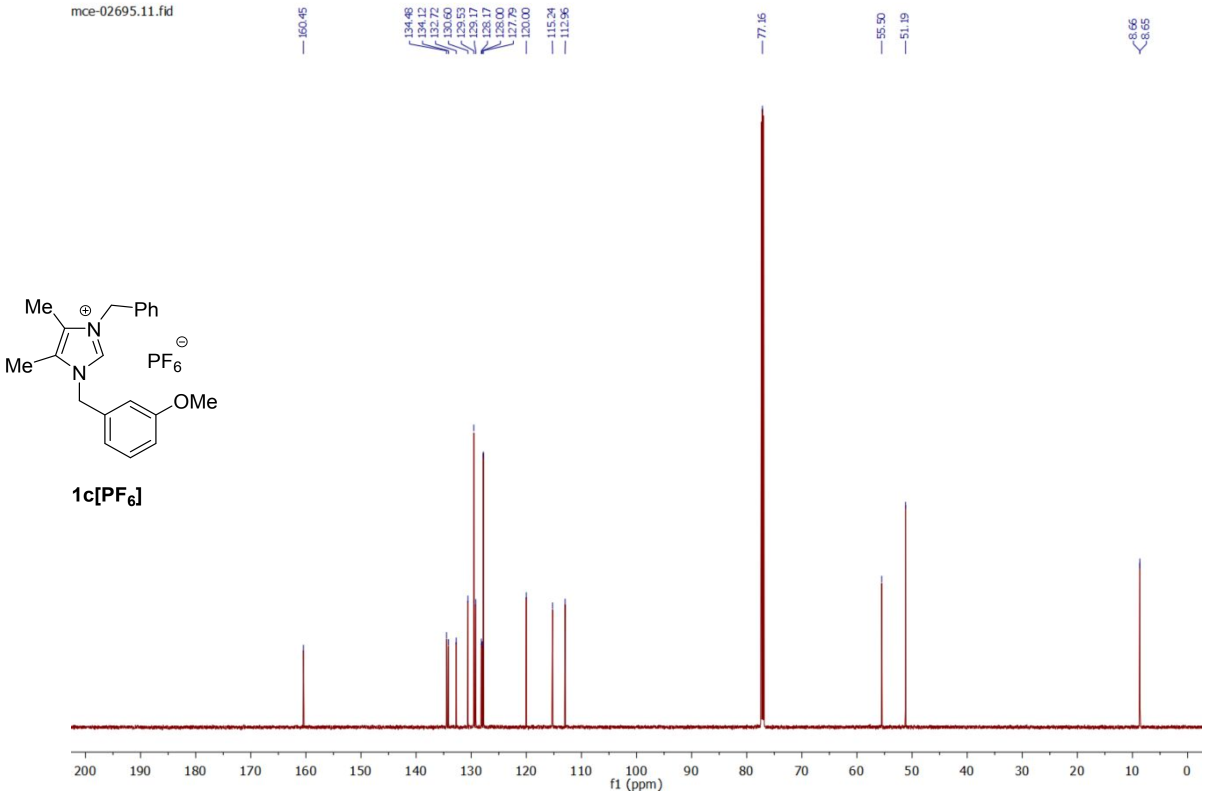

Fig. S22. The ^{13}C NMR (151 MHz, $CDCl_3$) spectrum for compound **1c[PF$_6$]**.

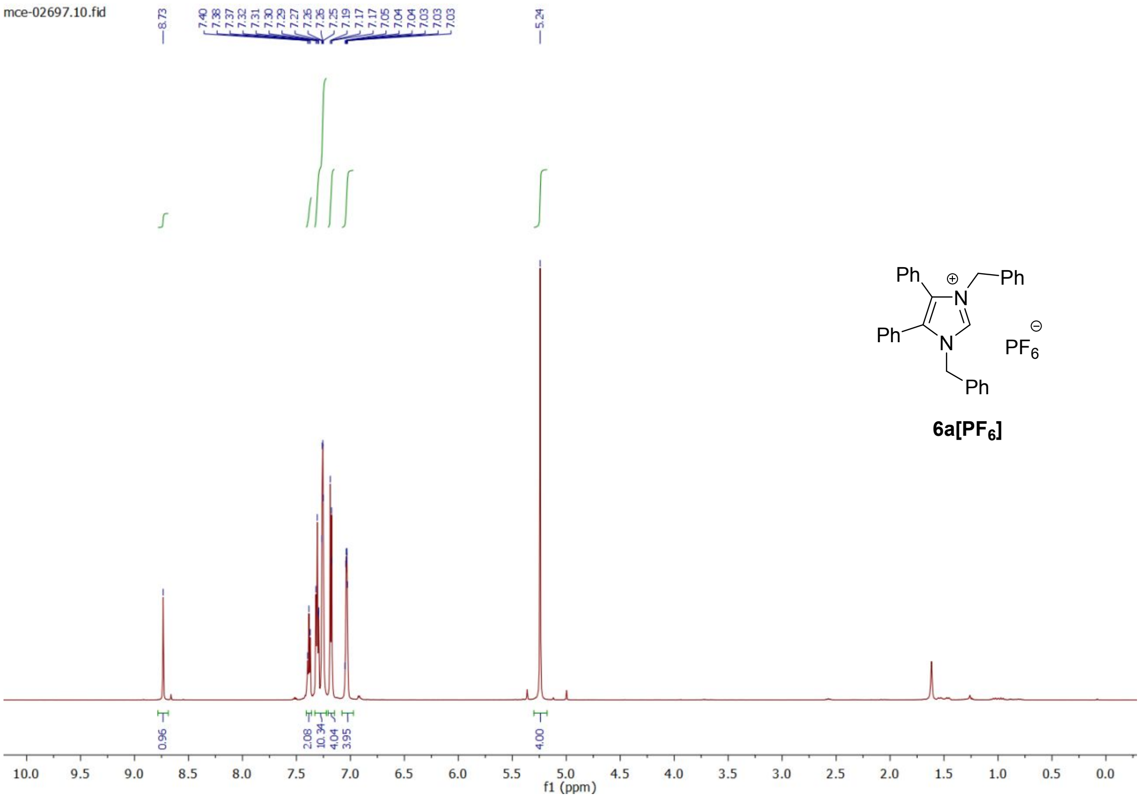

Fig. S23. The ^{1}H NMR (600 MHz, $CDCl_3$) spectrum for compound **6a[PF$_6$]**.

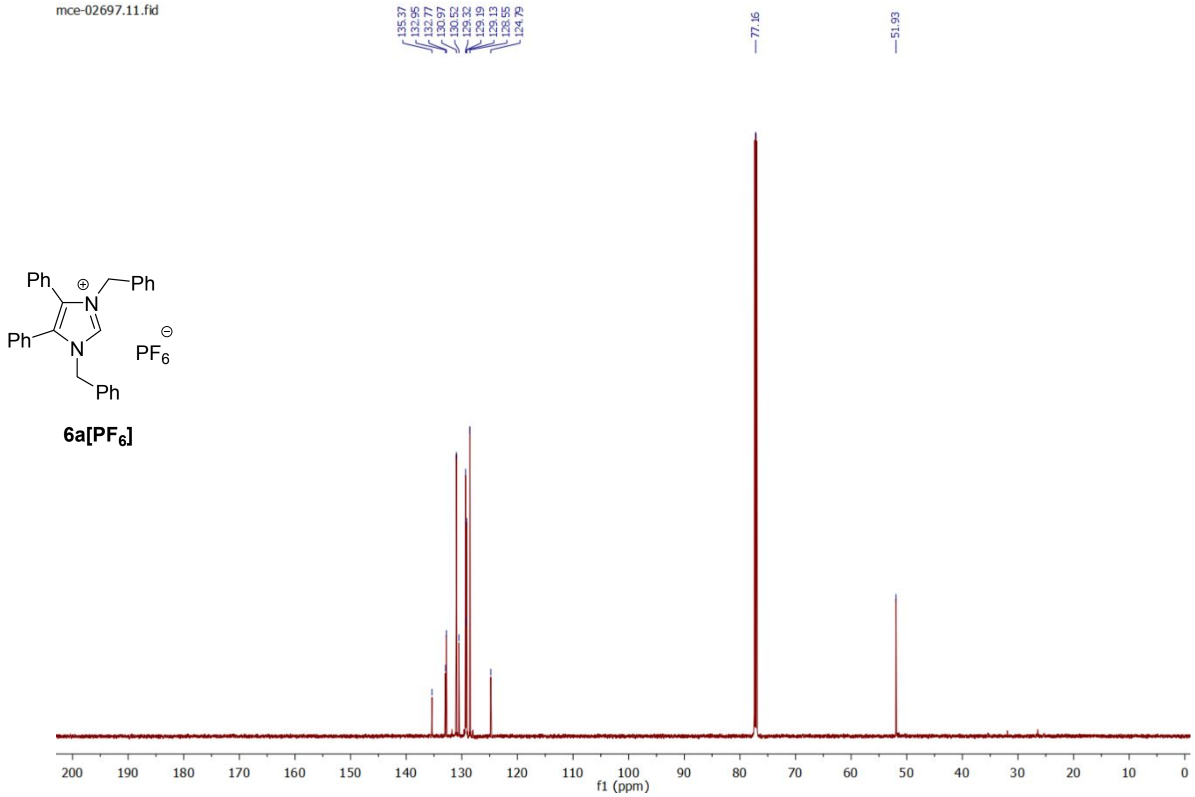

Fig. S24. The ^{13}C NMR (151 MHz, $CDCl_3$) spectrum for compound **6a[PF$_6$]**.

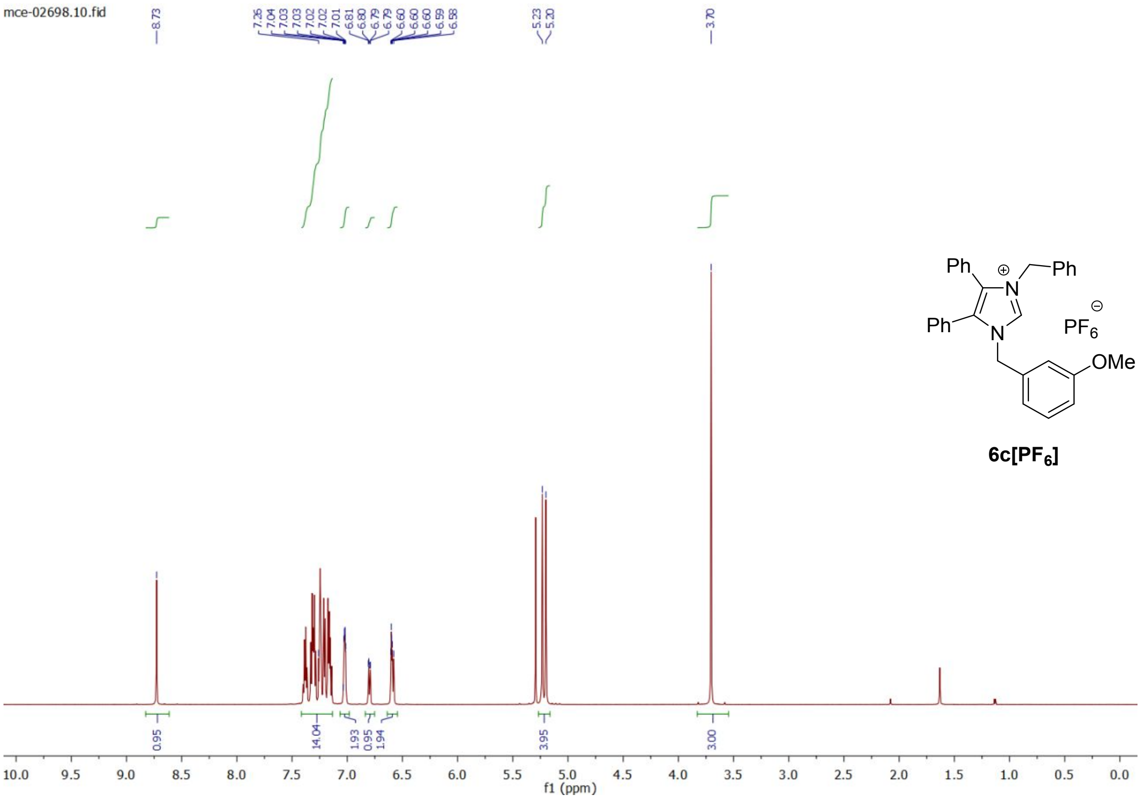

Fig. S25. The ^{1}H NMR (600 MHz, $CDCl_3$) spectrum for compound **6c[PF$_6$]**.

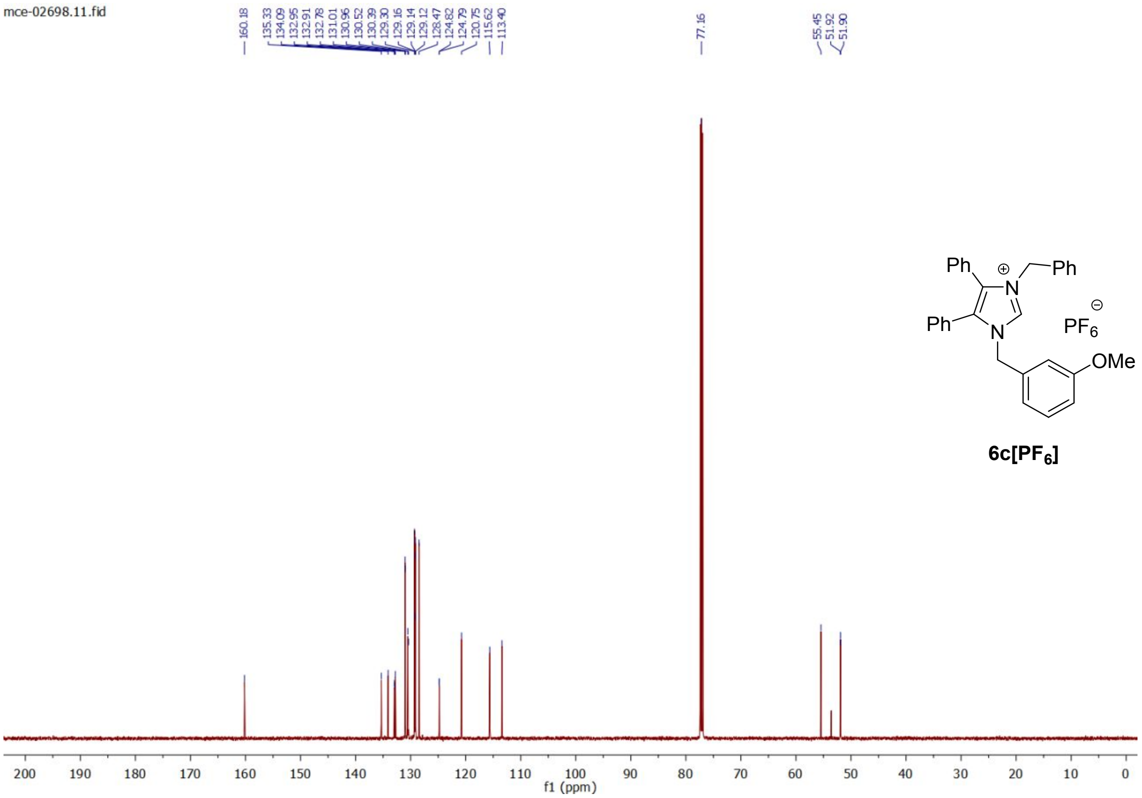

Fig. S26. The ^{13}C NMR (151 MHz, $CDCl_3$) spectrum for compound **6c[PF$_6$]**.

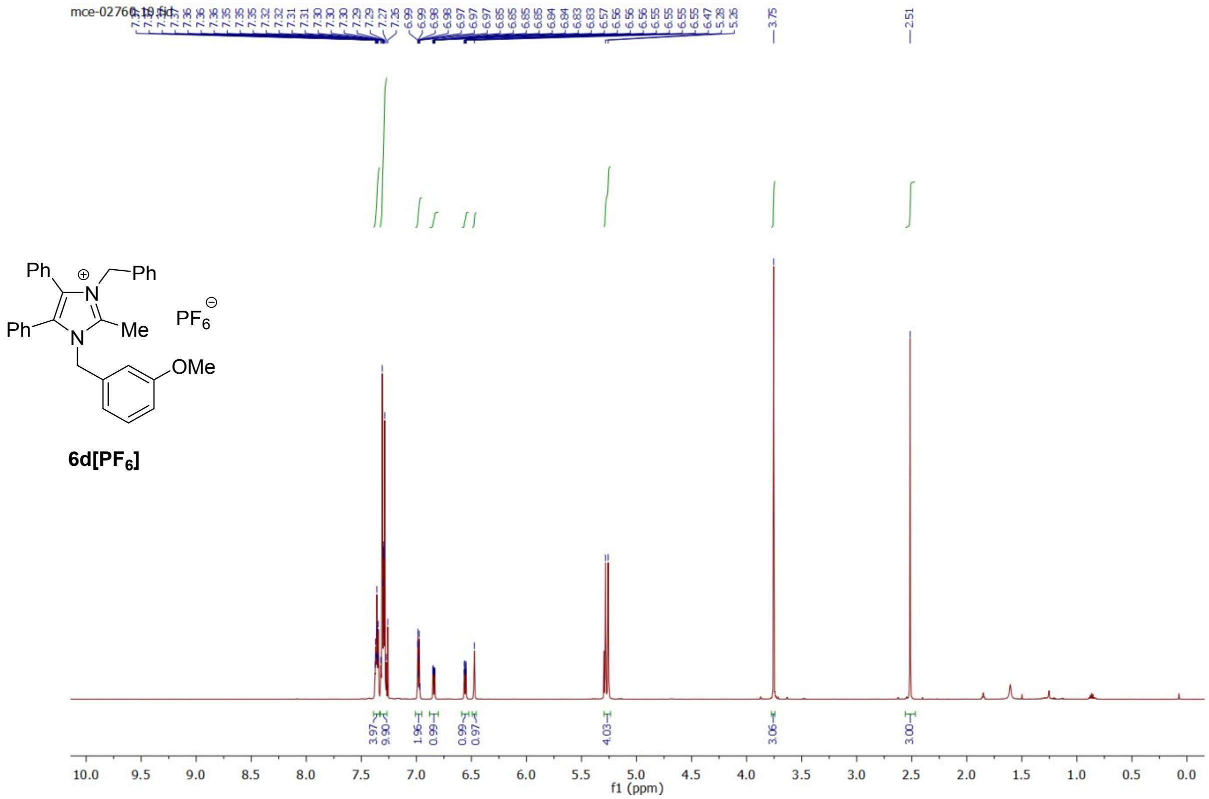

Fig. S27. The ^{1}H NMR (600 MHz, $CDCl_3$) spectrum for compound **6d[PF$_6$]**.

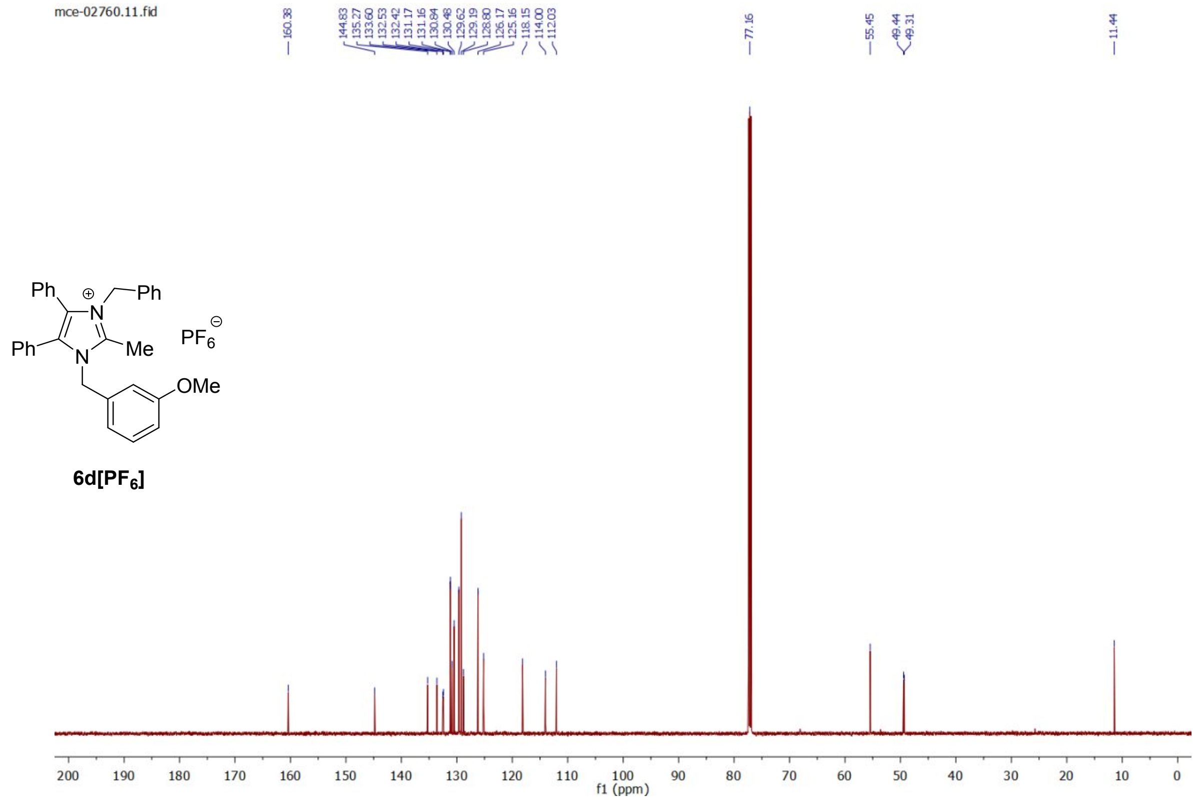

Fig. S28. The ^{13}C NMR (151 MHz, $CDCl_3$) spectrum for compound **6d[PF$_6$]**.

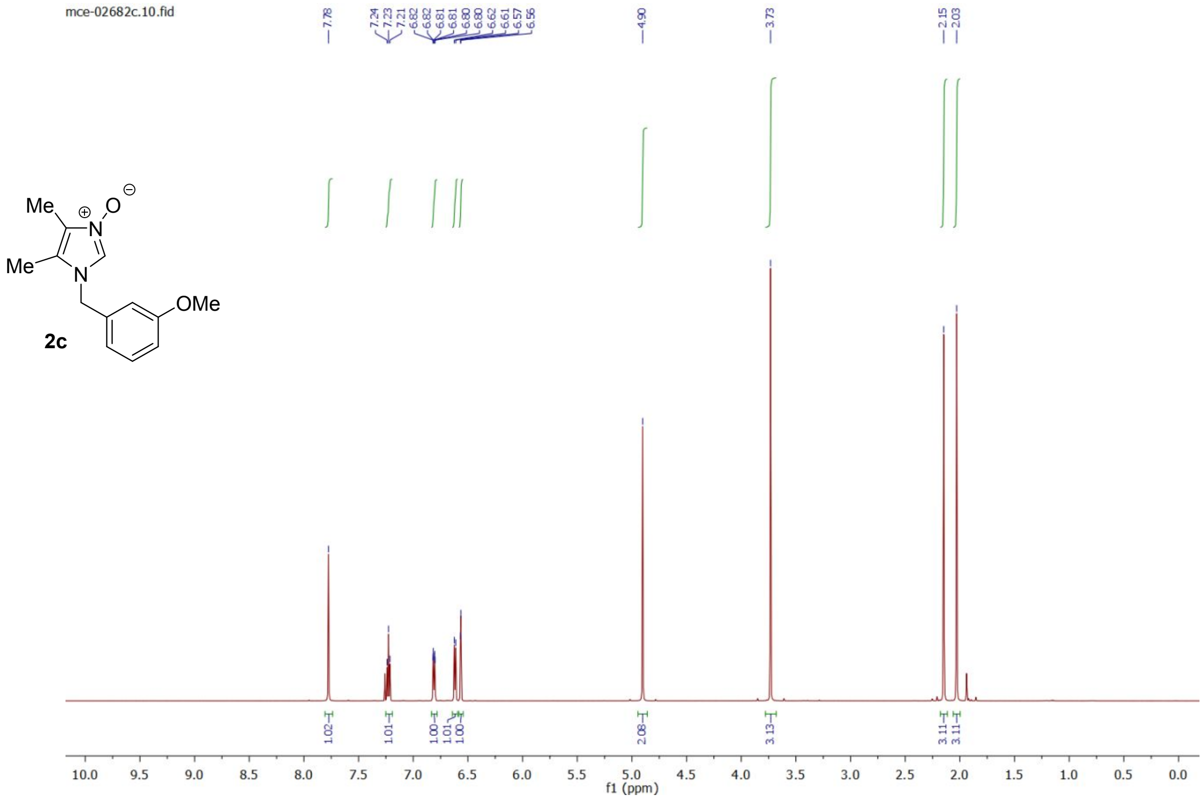

Fig. S29. The ^{1}H NMR (600 MHz, $CDCl_3$) spectrum for compound **2c**.

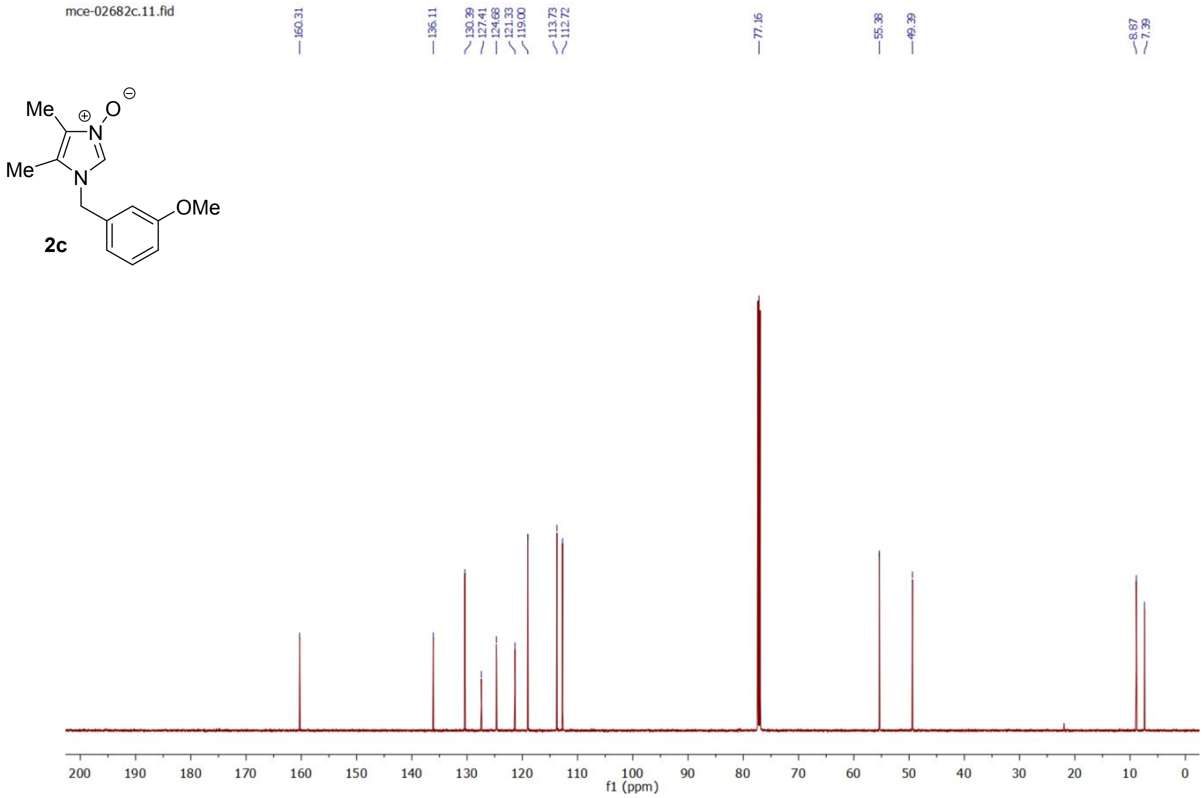

Fig. S30. The ^{13}C NMR (151 MHz, $CDCl_3$) spectrum for compound **2c**.

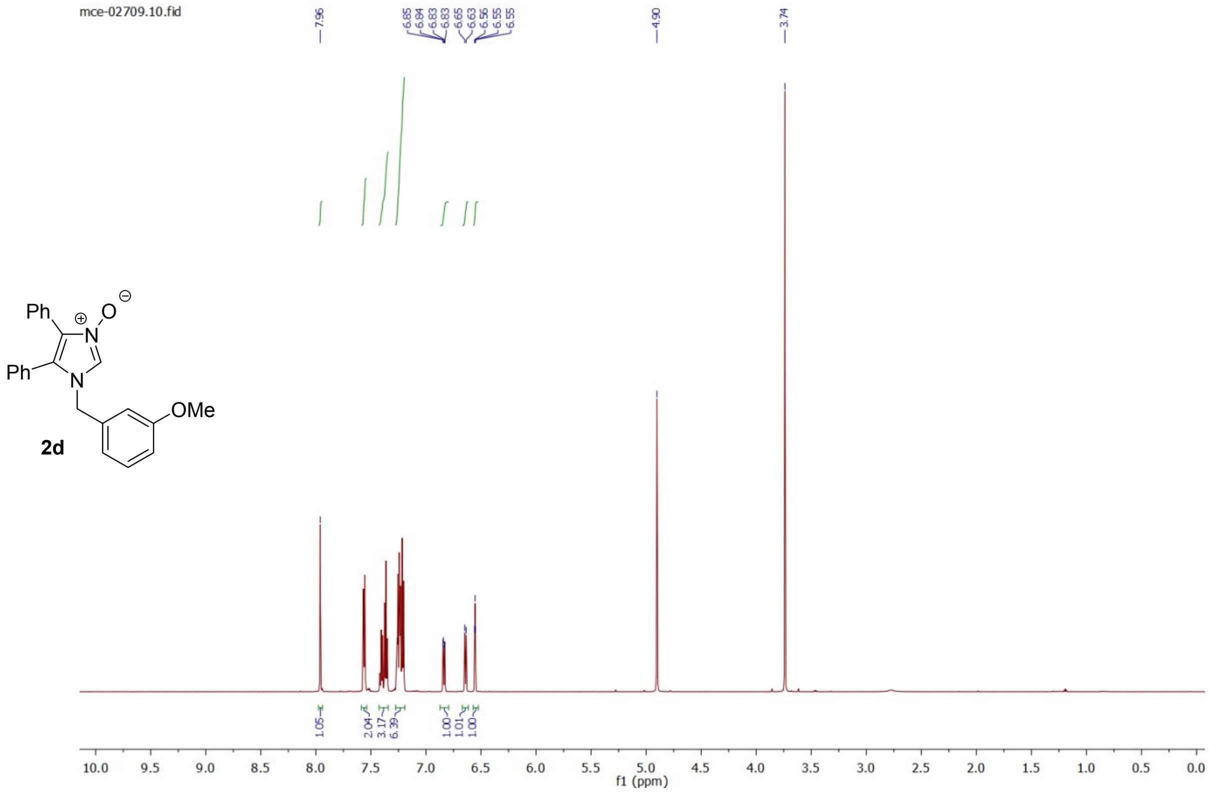

Fig. S31. The ^{1}H NMR (600 MHz, $CDCl_3$) spectrum for compound **2d**.

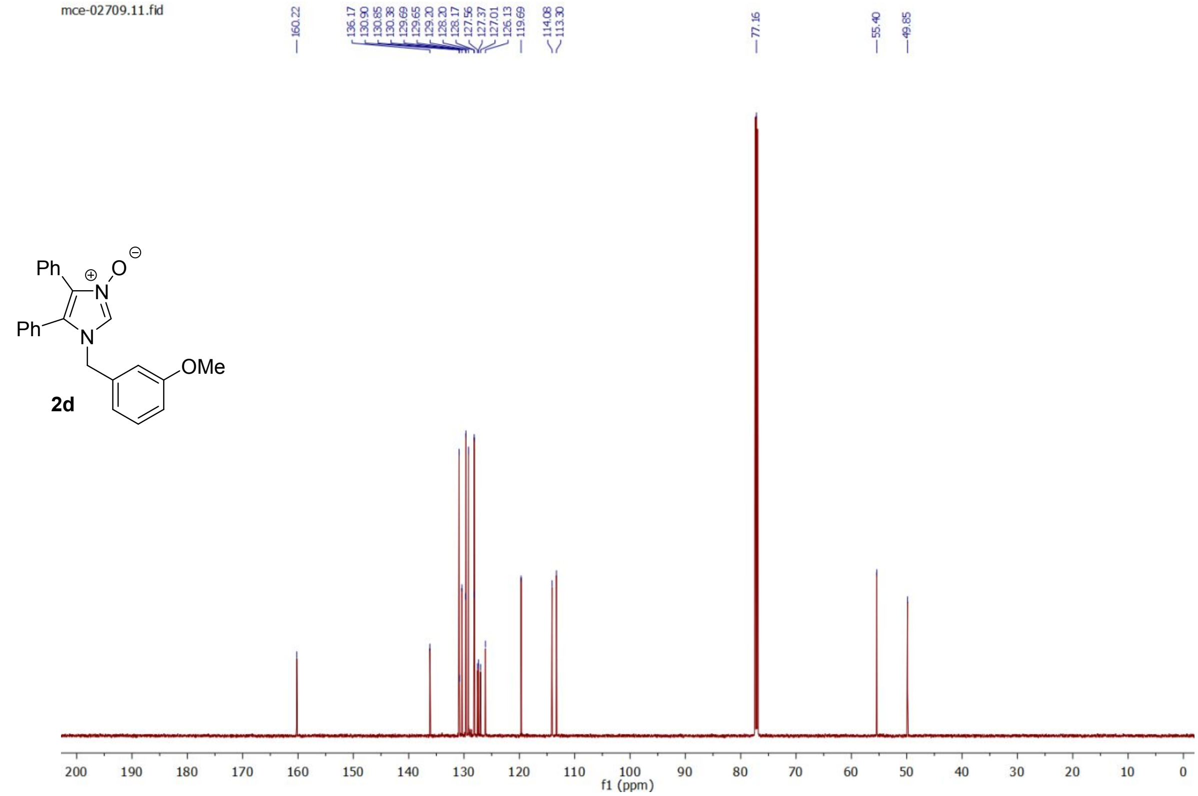

Fig. S32. The ^{13}C NMR (151 MHz, $CDCl_3$) spectrum for compound **2d**.

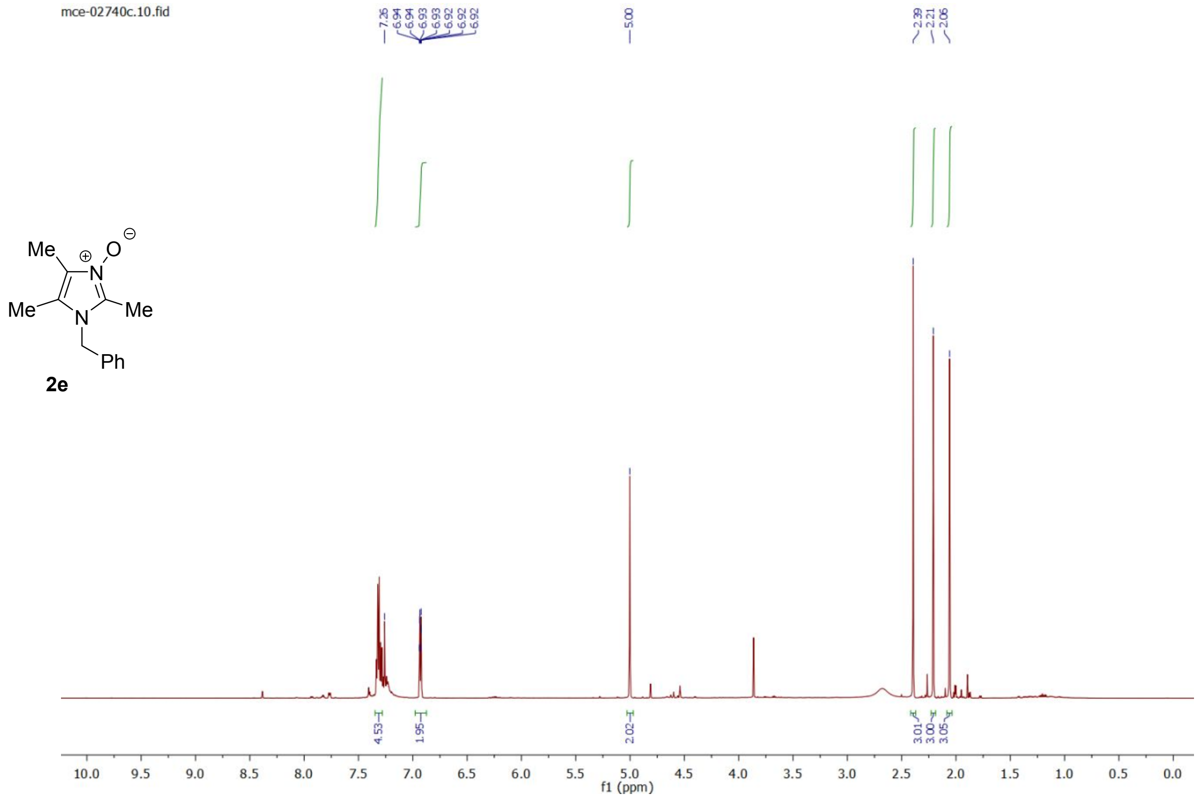

Fig. S33. The ^{1}H NMR (600 MHz, $CDCl_3$) spectrum for compound **2e**.

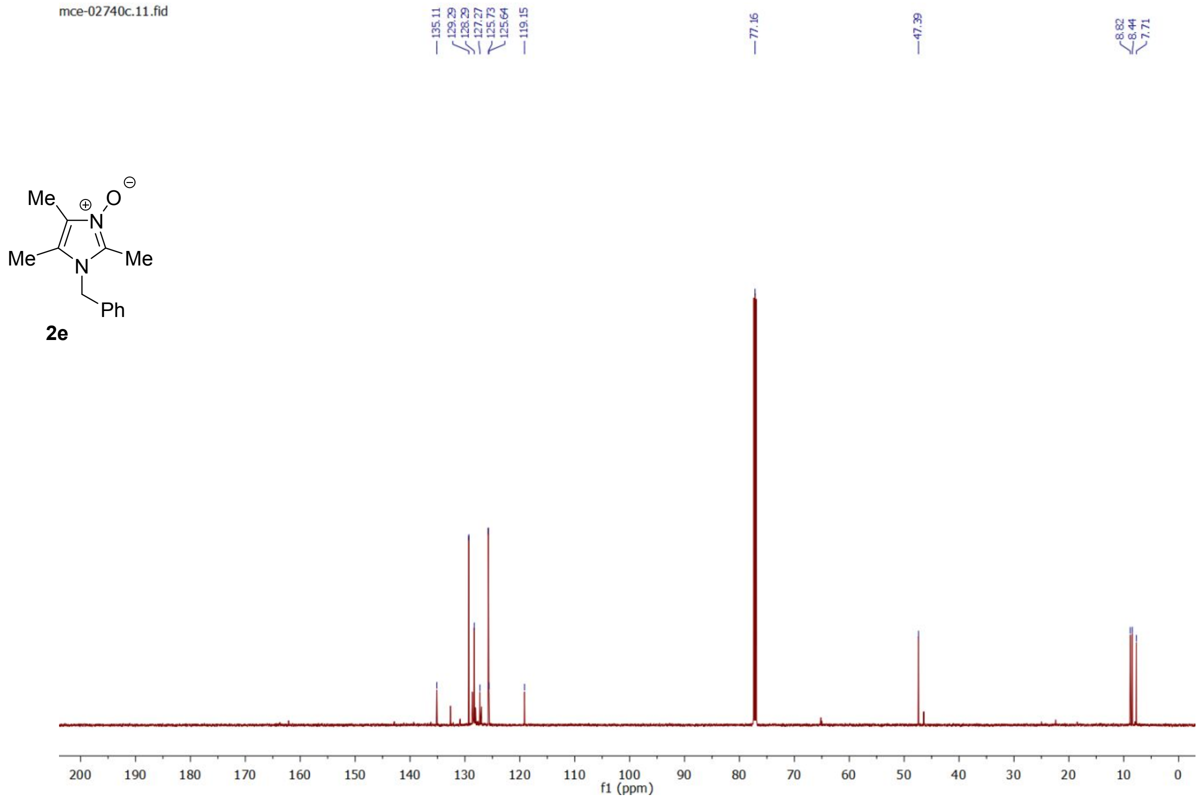

Fig. S34. The ^{13}C NMR (151 MHz, $CDCl_3$) spectrum for compound **2e**.

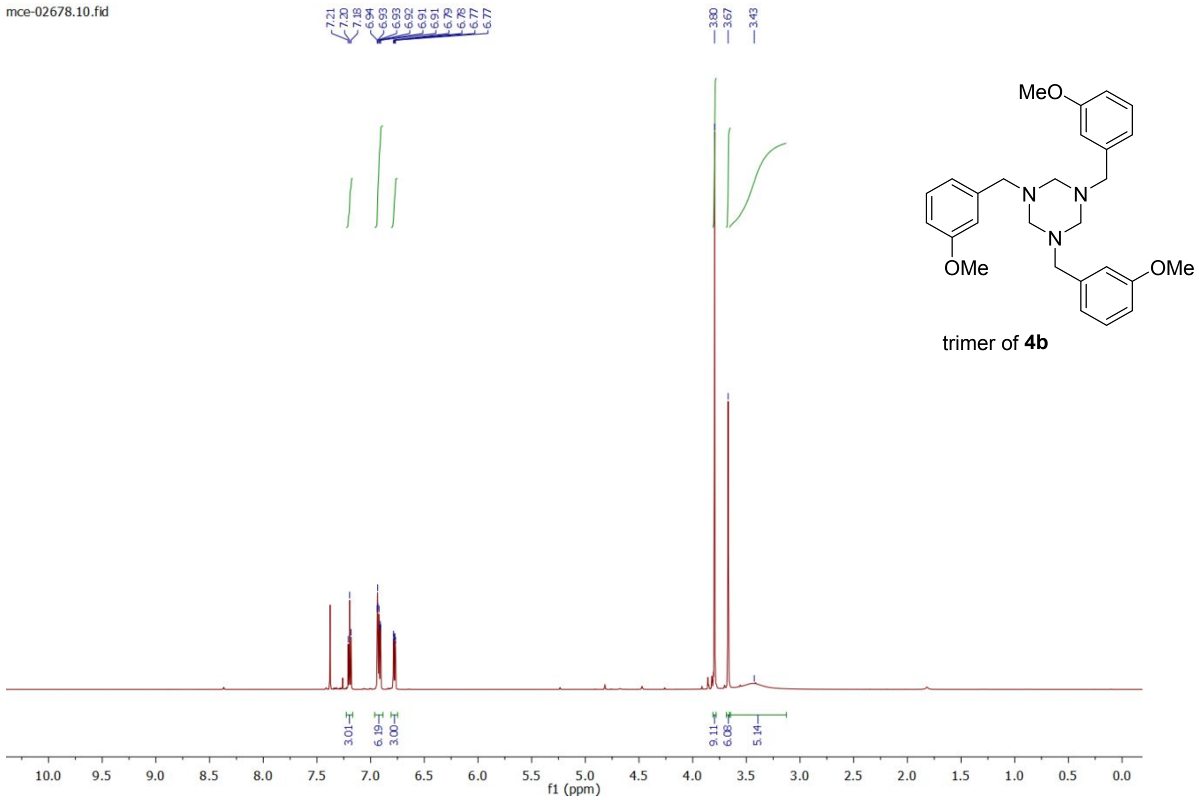

Fig. S35. The ^{1}H NMR (600 MHz, $CDCl_3$) spectrum for compound **4b**.

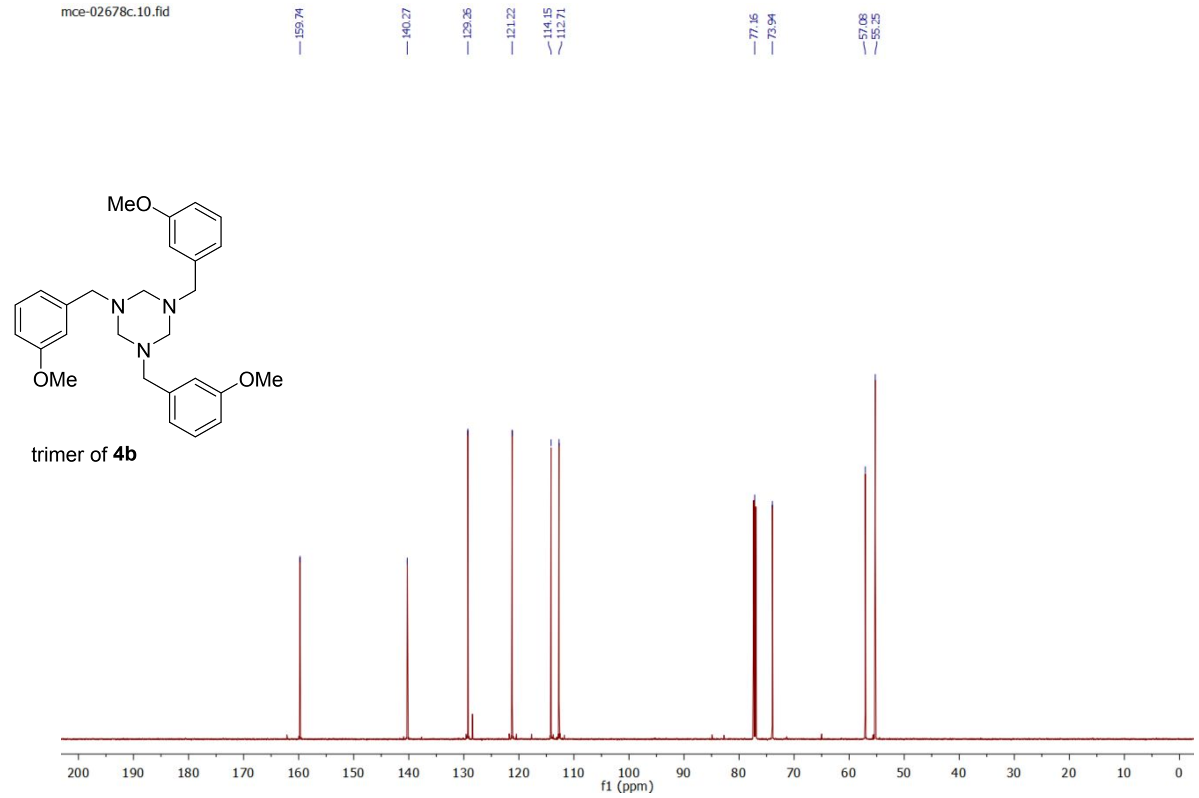

Fig. S36. The ^{13}C NMR (151 MHz, $CDCl_3$) spectrum for compound **4b**.

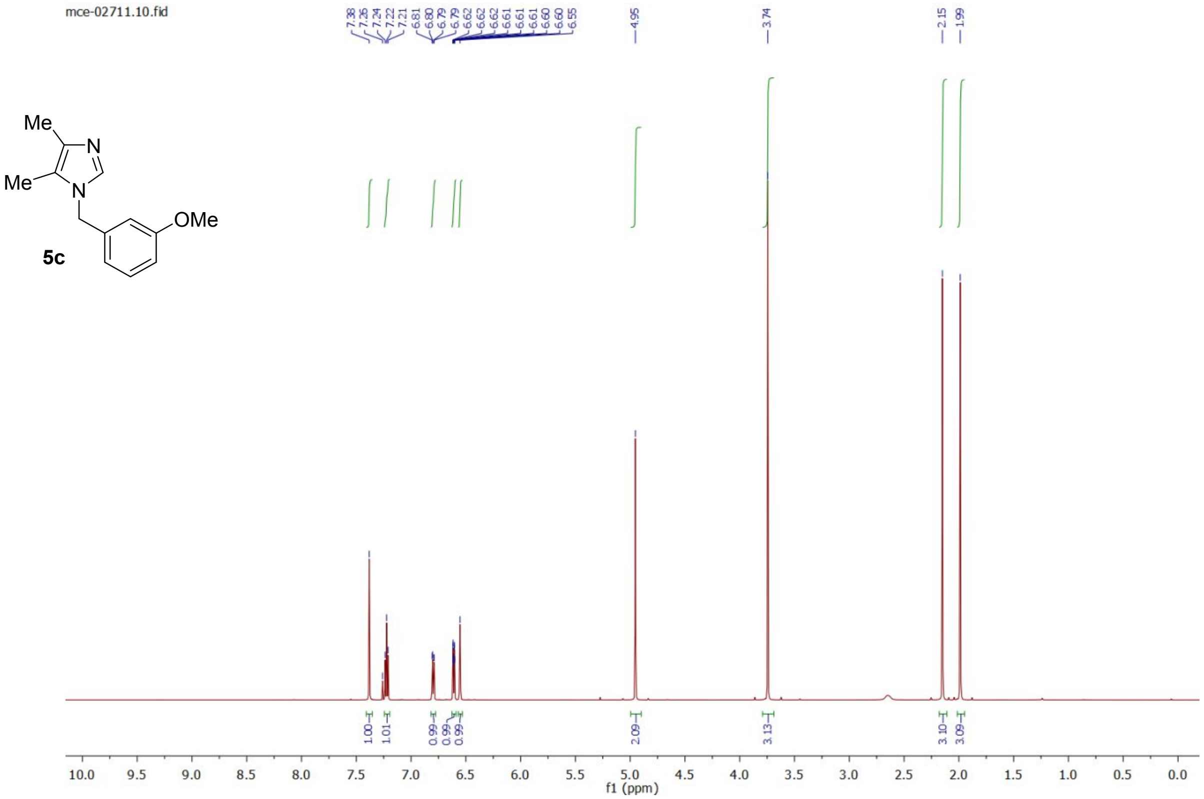

Fig. S37. The ^{1}H NMR (600 MHz, $CDCl_3$) spectrum for compound **5c**.

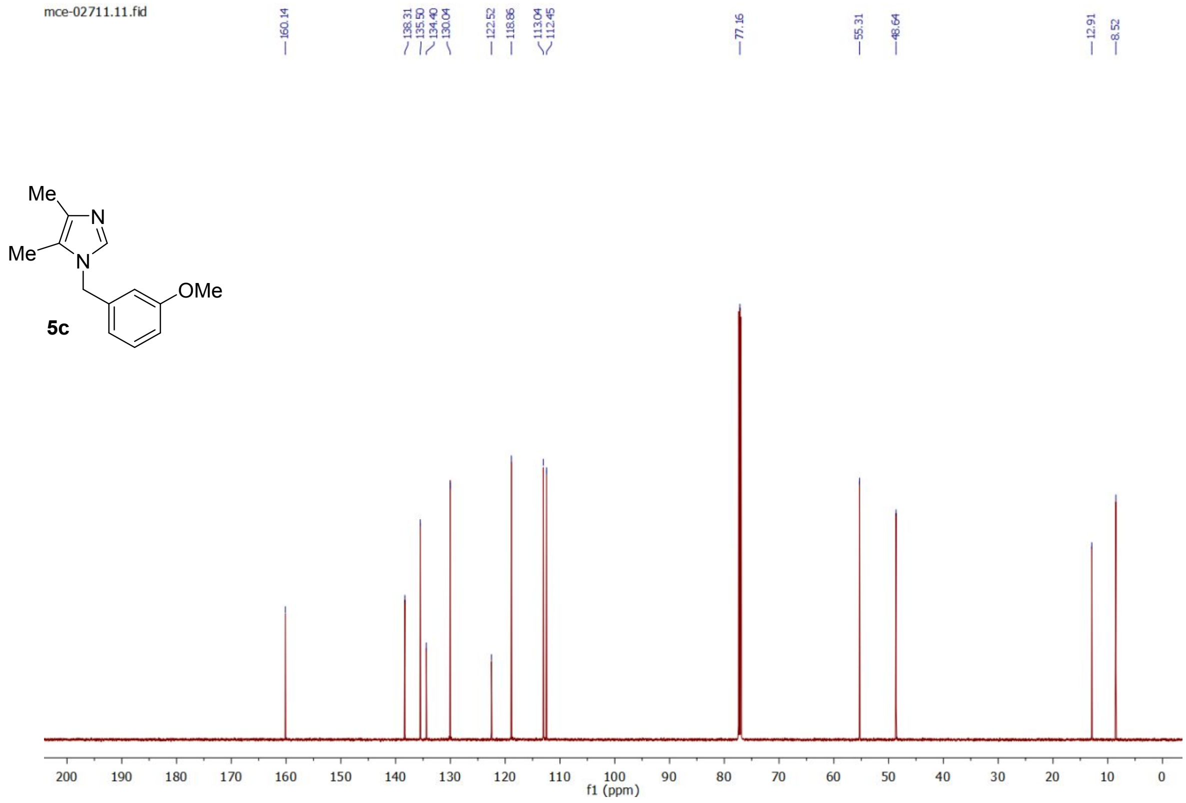

Fig. S38. The ^{13}C NMR (151 MHz, $CDCl_3$) spectrum for compound **5c**.

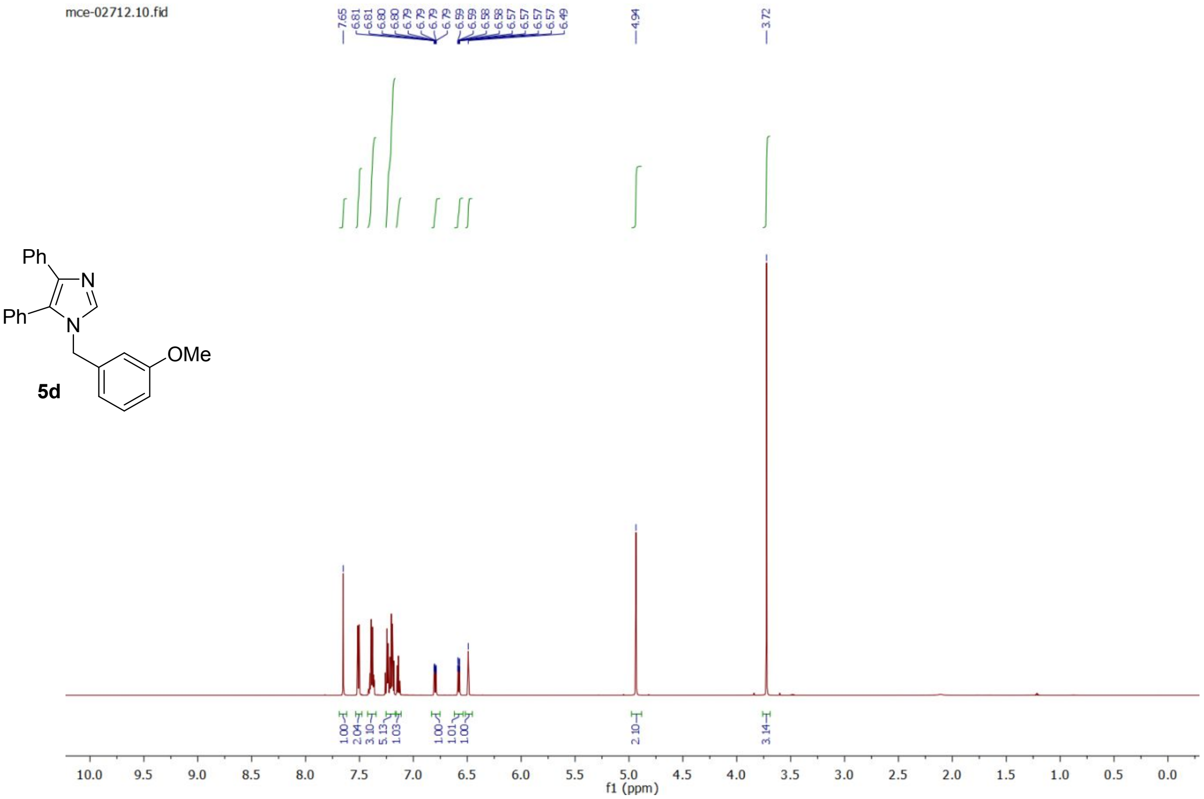

Fig. S39. The ^{1}H NMR (600 MHz, $CDCl_3$) spectrum for compound **5d**.

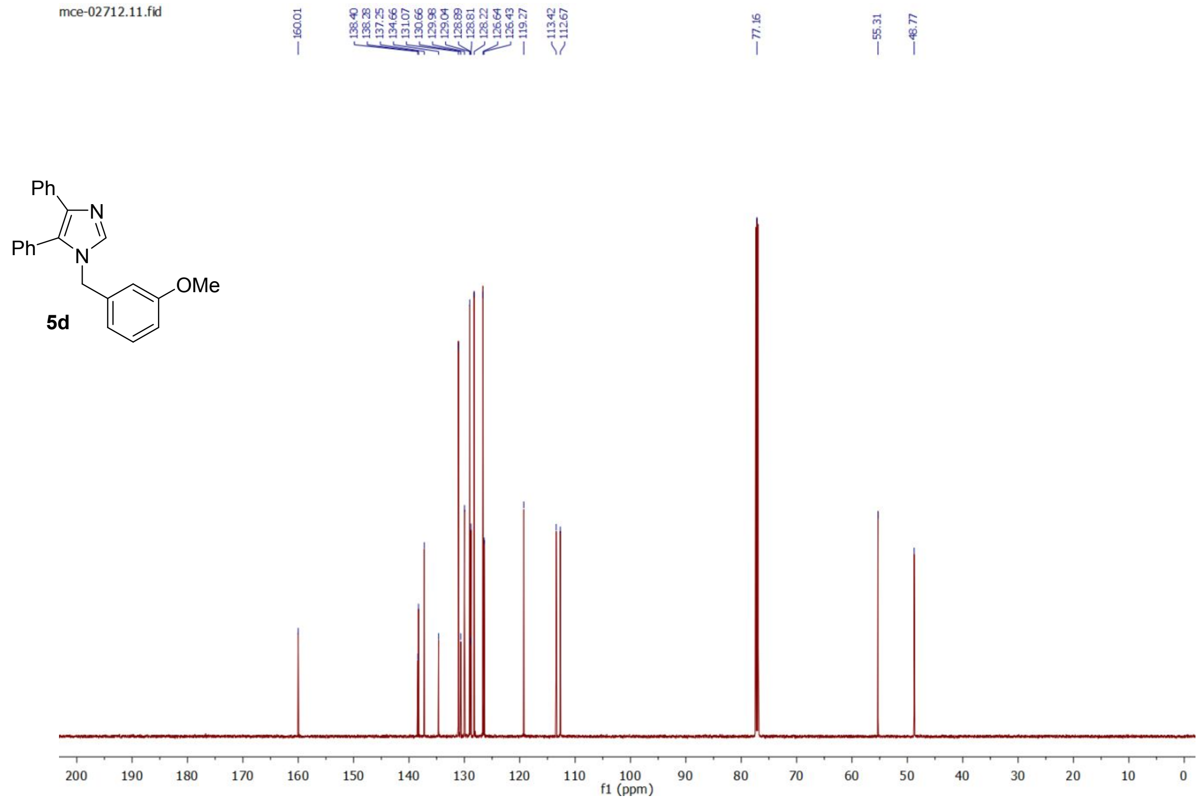

Fig. S40. The ^{13}C NMR (151 MHz, $CDCl_3$) spectrum for compound **5d**.

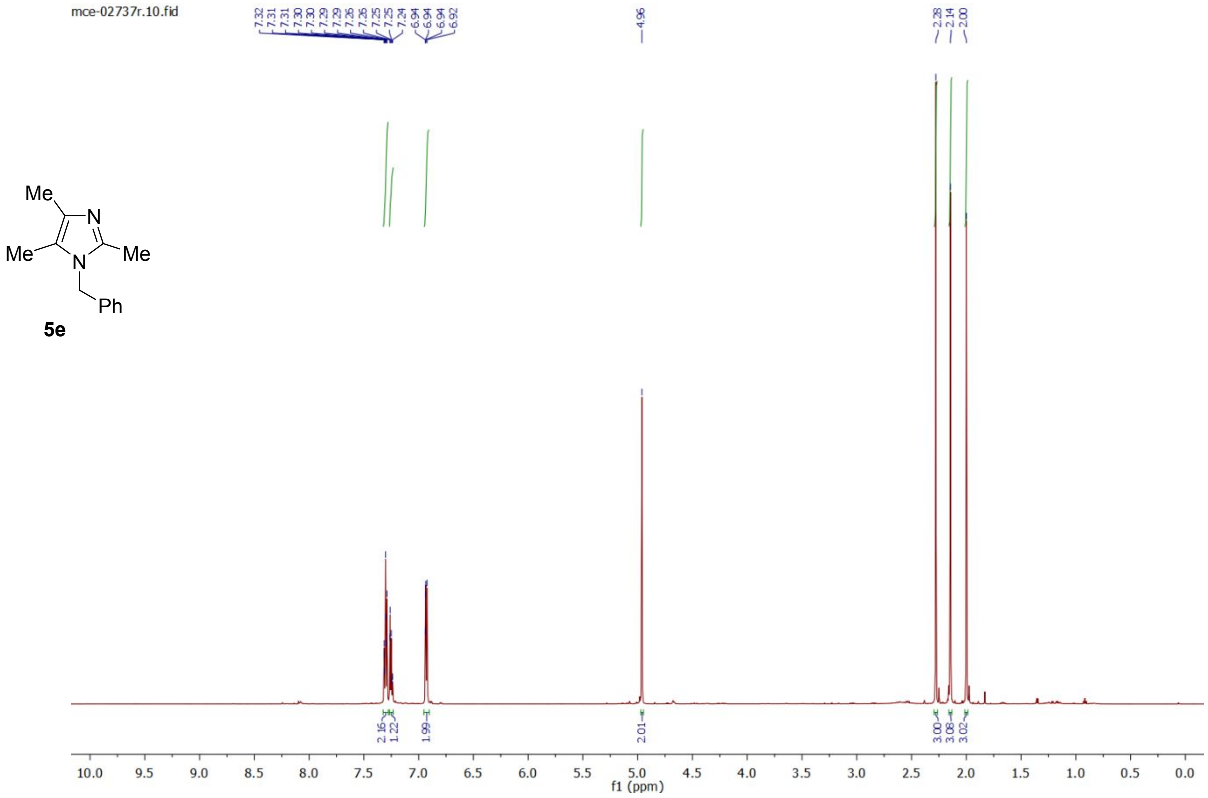

Fig. S41. The ^{1}H NMR (600 MHz, $CDCl_3$) spectrum for compound **5e**.

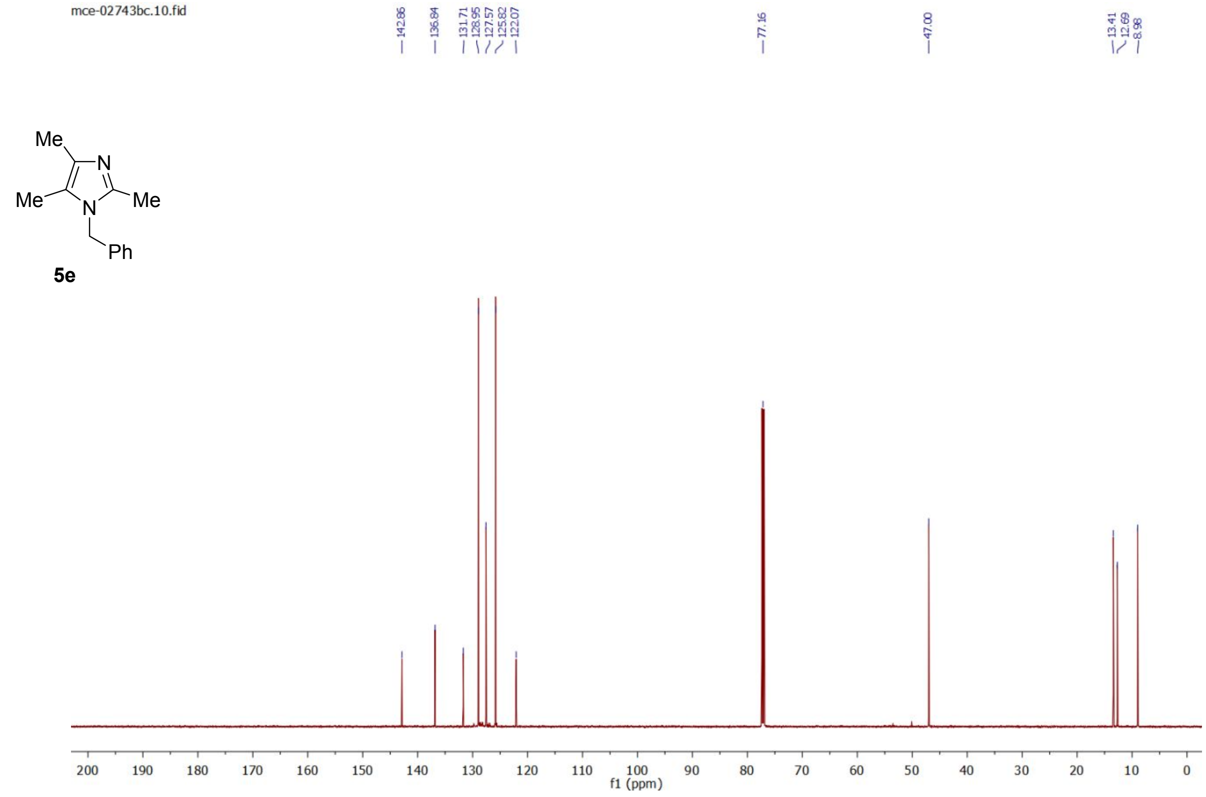

Fig. S42. The ^{13}C NMR (151 MHz, $CDCl_3$) spectrum for compound **5e**.

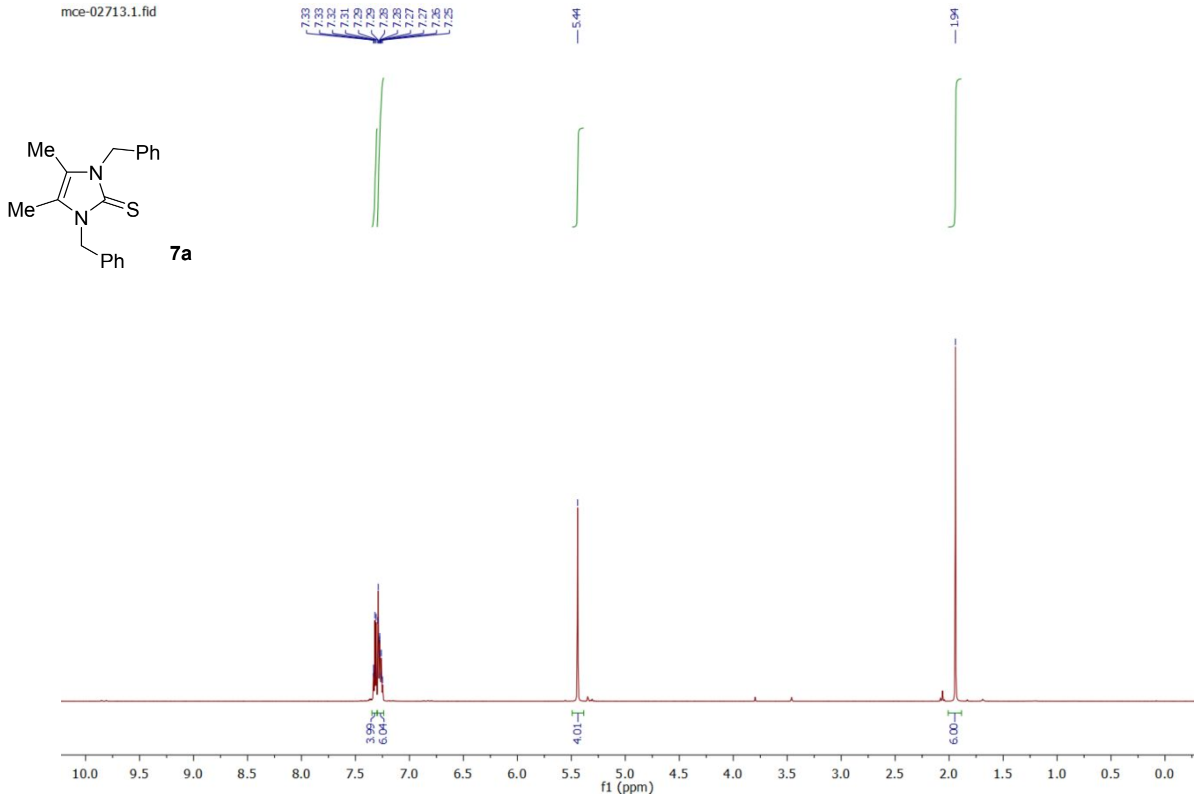

Fig. S43. The ^{1}H NMR (600 MHz, $CDCl_3$) spectrum for compound **7a**.

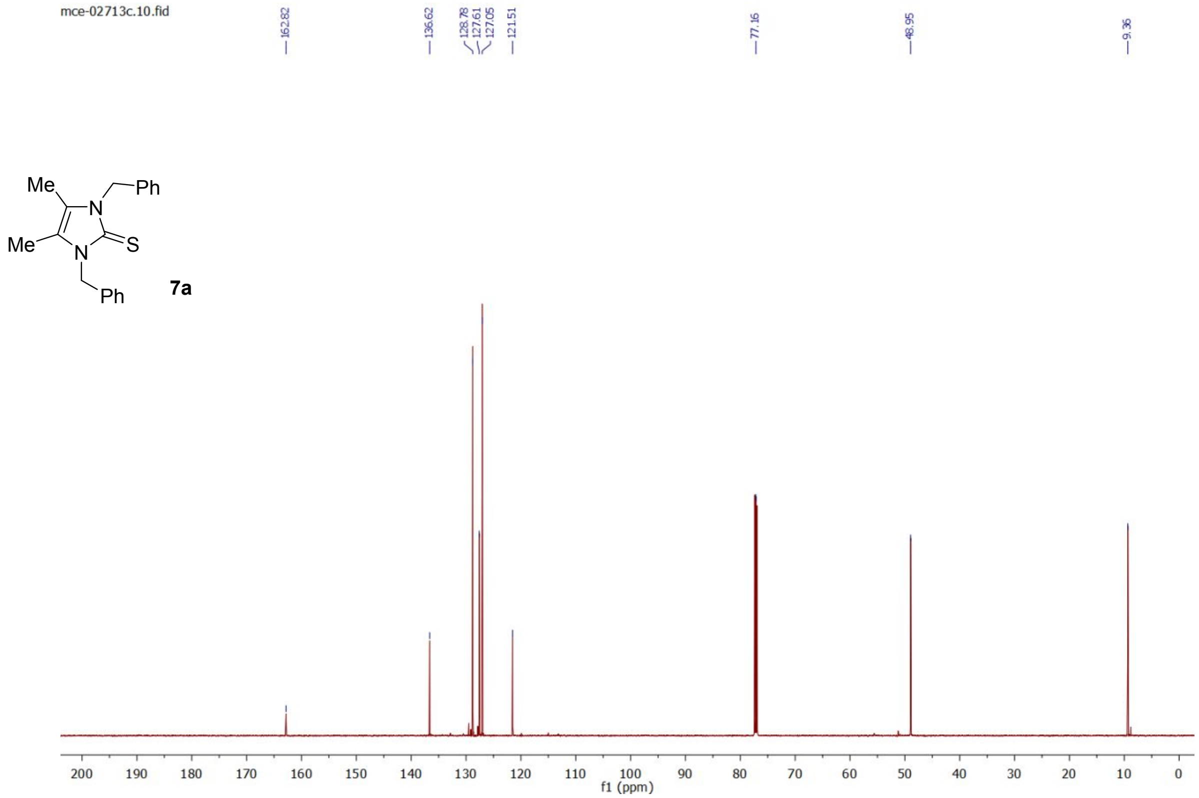

Fig. S44. The ^{13}C NMR (151 MHz, $CDCl_3$) spectrum for compound **7a**.

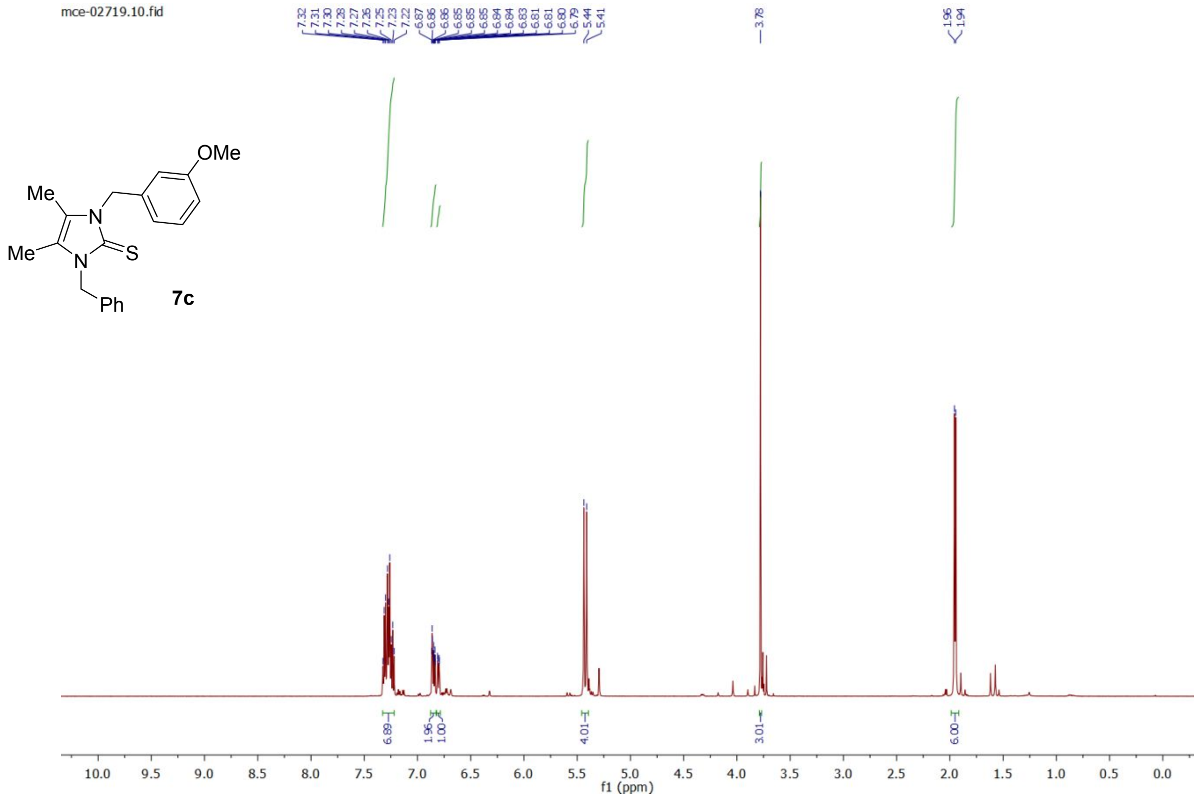

Fig. S45. The ^{1}H NMR (600 MHz, $CDCl_3$) spectrum for compound **7c**.

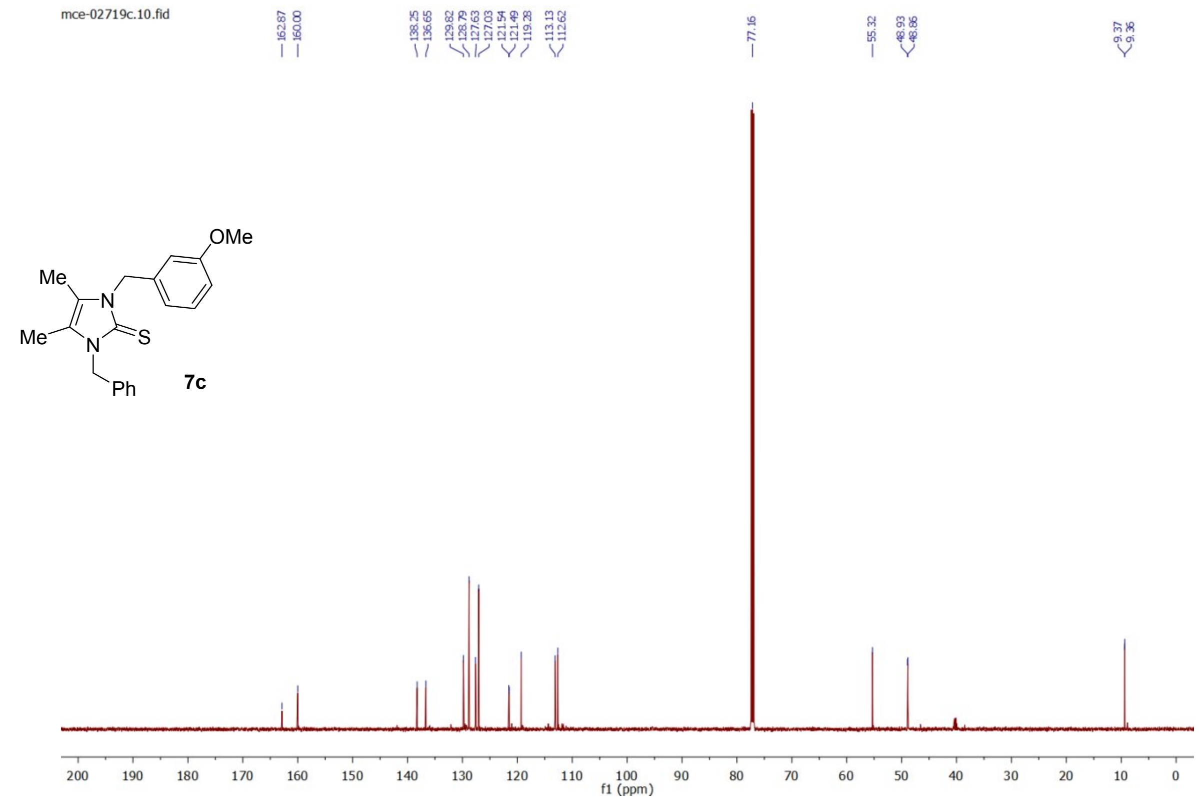

Fig. S46. The ^{13}C NMR (151 MHz, $CDCl_3$) spectrum for compound **7c**.

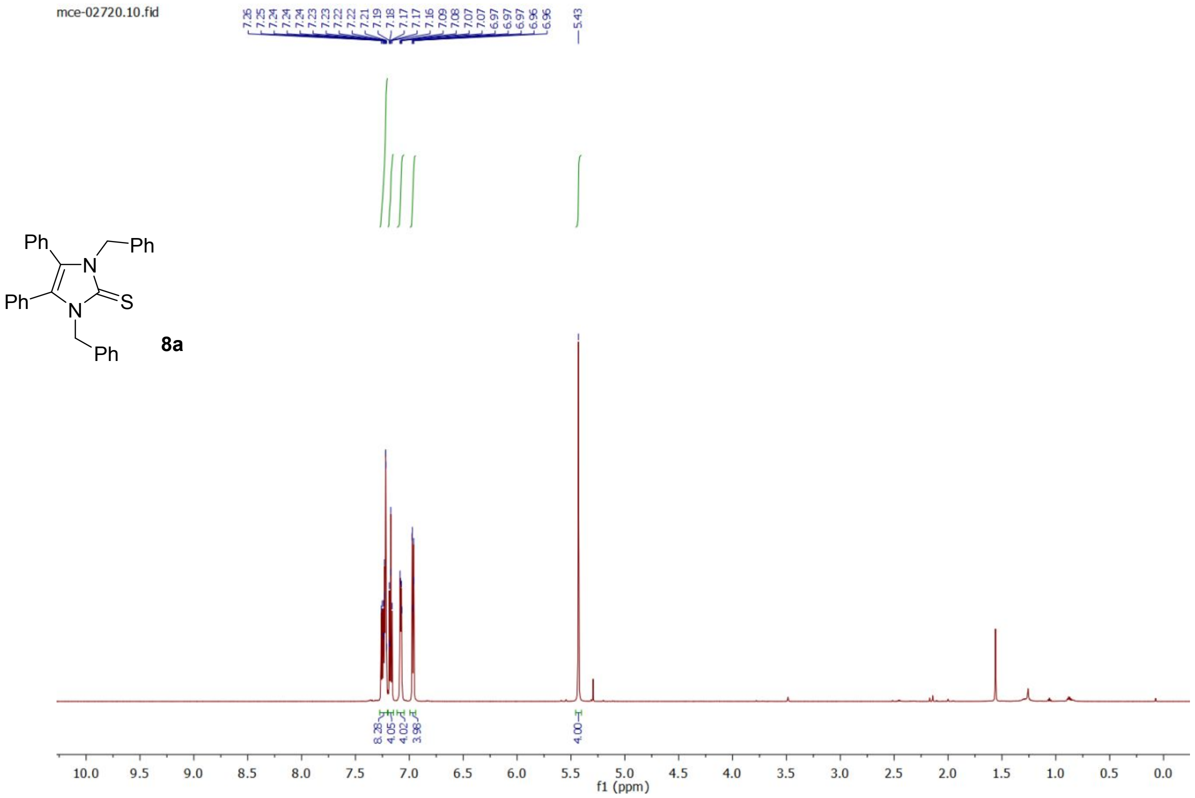

Fig. S47. The ^{1}H NMR (600 MHz, $CDCl_3$) spectrum for compound **8a**.

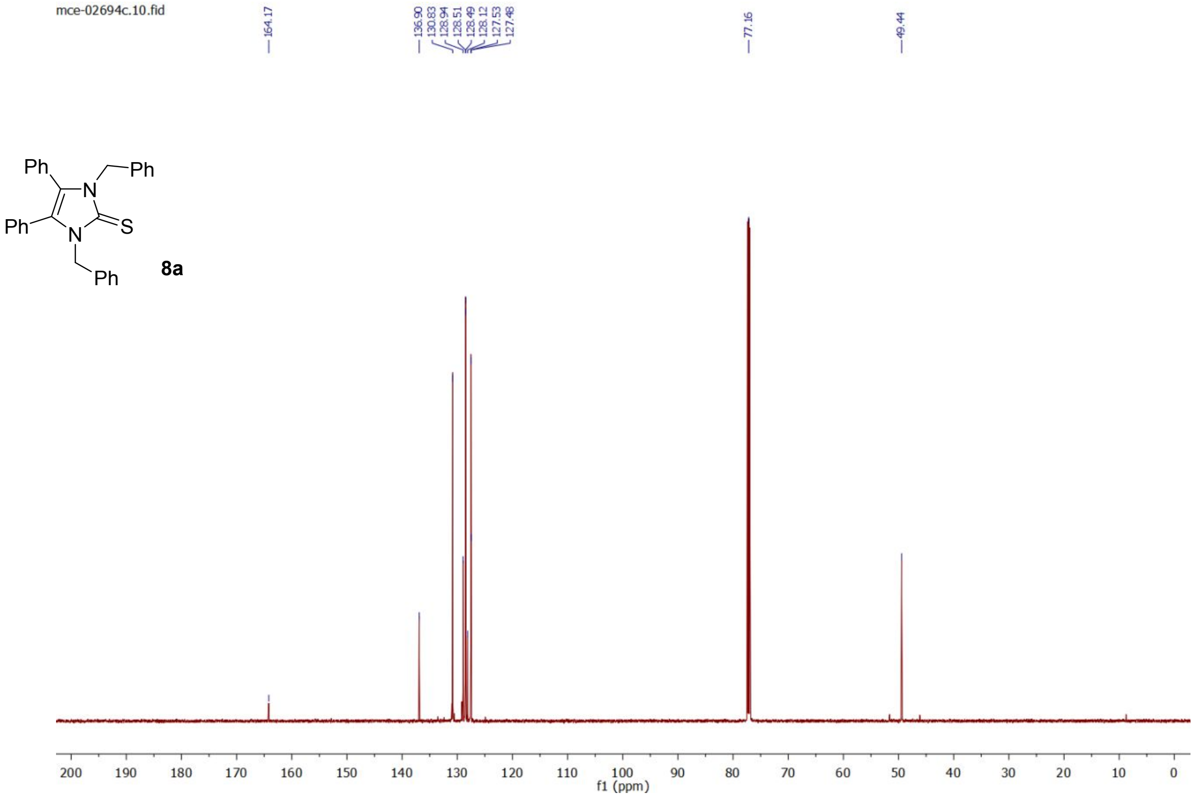

Fig. S48. The ^{13}C NMR (151 MHz, $CDCl_3$) spectrum for compound **8a**.

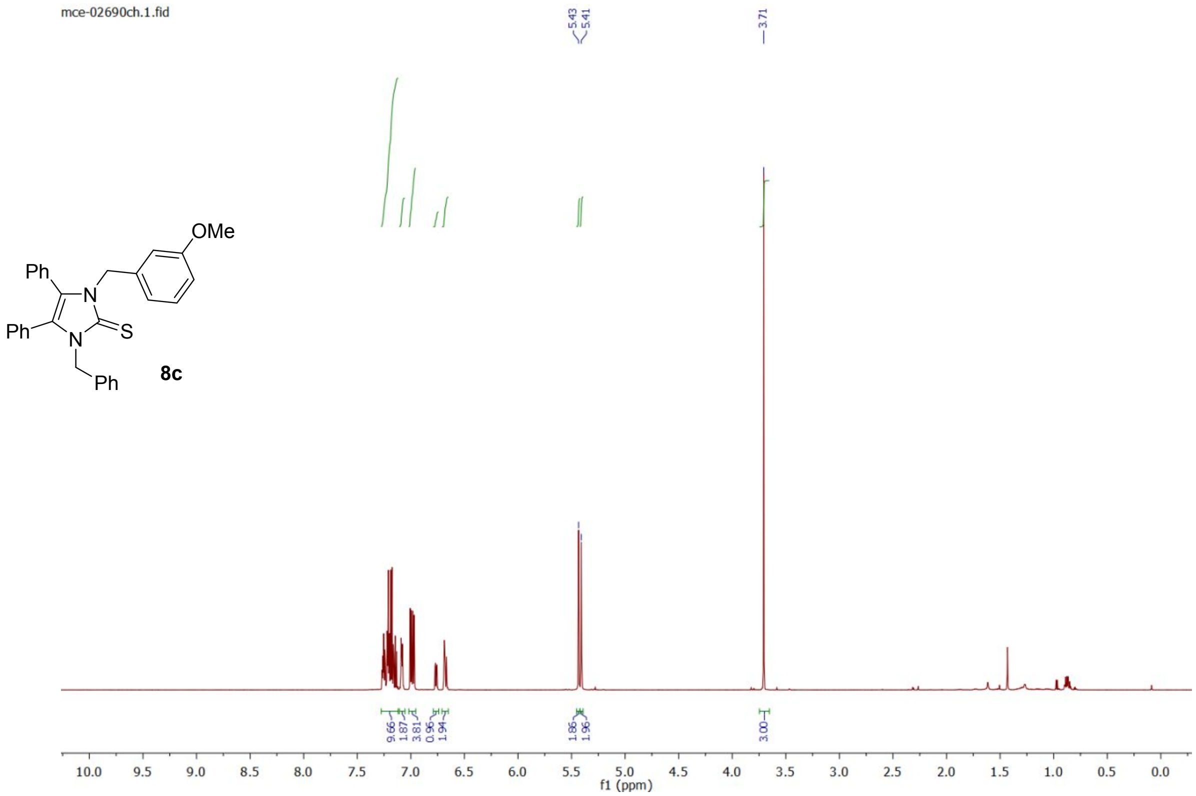

Fig. S49. The ^{1}H NMR (600 MHz, $CDCl_3$) spectrum for compound **8c**.

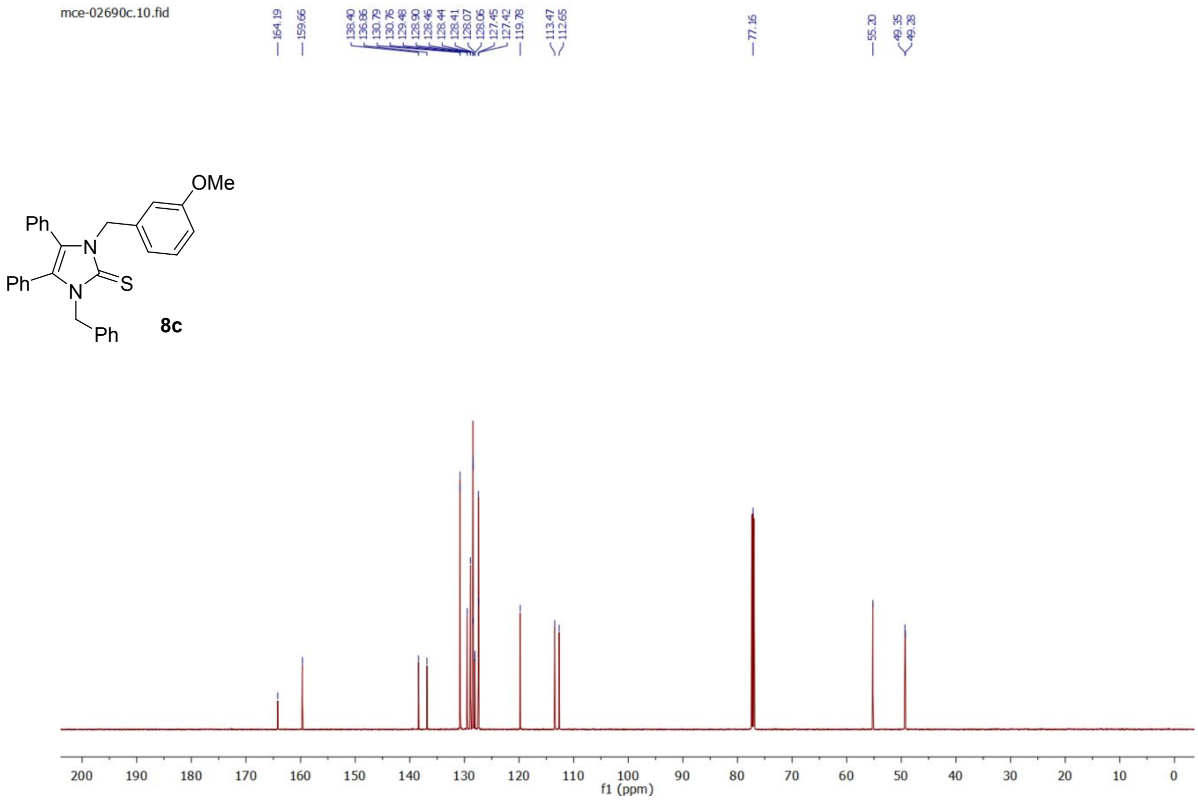

Fig. S50. The ^{13}C NMR (151 MHz, $CDCl_3$) spectrum for compound **8c**.

Crystallographic analysis of 1c[PF_6]

The crystals of compound **1c[PF_6]** were of good quality. A suitable crystal was selected and measured on a XtaLAB Synergy, Dualflex, Pilatus 300K diffractometer. The crystal was mounted in inert oil on nylon loops and kept at 100.00(10) K during data collection. Measurements for compound **1c**[PF_6] were performed using mirror-focused Cu-Kα radiation. Absorption corrections were implemented on the basis of multi-scans. Using Olex2 [1], the structure was solved with the XT [2] structure solution program using Intrinsic Phasing and refined with the XL [3] refinement package using Least Squares minimization. Hydrogen atoms were included using rigid methyl groups or a riding model starting from calculated positions. Additionally, complete data have been deposited with the Cambridge Crystallographic Data Centre under the numbers CCDC-2059690. Copies of the data can be obtained free of charge from www.ccdc.cam.ac.uk/structures/.

Table S1. Crystal data and structure refinement for **1c[PF_6]**.

Identification code	mg1315_1
Empirical formula	$C_{20}H_{23}F_6N_2OP$
Formula weight	452.37
Temperature/K	100.00(10)
Crystal system	triclinic
Space group	P-1
a/Å	9.8930(3)
b/Å	11.1547(4)
c/Å	11.1640(4)
α/°	60.849(4)
β/°	84.409(3)
γ/°	71.453(3)
Volume/Å^3	1017.60(7)
Z	2
ρ_{calc}g/cm^3	1.476
μ/mm^{-1}	1.834
F(000)	468.0
Crystal size/mm^3	0.3 × 0.23 × 0.21
Radiation	Cu Kα (λ = 1.54184)
2Θ range for data collection/°	9.092 to 157.666
Index ranges	$-12 \leq h \leq 11$, $-14 \leq k \leq 12$, $-14 \leq l \leq 13$
Reflections collected	23177
Independent reflections	4125 [R_{int} = 0.0370, R_{sigma} = 0.0187]
Data/restraints/parameters	4125/0/275
Goodness-of-fit on F^2	1.032
Final R indexes [I>=2σ (I)]	R_1 = 0.0319, wR_2 = 0.0814
Final R indexes [all data]	R_1 = 0.0335, wR_2 = 0.0824
Largest diff. peak/hole / e Å^{-3}	0.25/-0.33

Table S2. Fractional Atomic Coordinates (×10^4) and Equivalent Isotropic Displacement Parameters (Å^2×10^3) for **1c[PF$_6$]**. U_{eq} is defined as 1/3 of of the trace of the orthogonalised U_{IJ} tensor.

Atom	*x*	*y*	*z*	U(eq)
P1	2766.0(3)	8747.8(3)	7214.8(3)	20.69(10)
F2	1670.0(9)	8426.6(10)	8402.1(8)	30.6(2)
F4	3859.4(9)	9073.5(9)	6018.3(8)	32.0(2)
F6	2457.6(10)	10344.3(9)	7008.0(9)	32.3(2)
F3	4038.6(8)	8139.6(9)	8335.0(8)	28.26(19)
F7	3075.3(10)	7160.5(9)	7412.8(9)	33.0(2)
F5	1489.4(9)	9366.7(10)	6084.3(9)	34.4(2)
O22	9809.2(10)	5368.3(11)	2790.5(10)	28.0(2)
N3	7884.1(12)	7517.5(12)	7429.1(11)	20.9(2)
N1	6757.1(11)	6650.8(11)	6643.2(11)	19.7(2)
C5	6873.1(13)	5815.8(13)	8065.8(13)	19.6(3)
C16	7625.2(14)	9479.5(14)	7973.4(13)	21.7(3)
C7	6974.7(14)	5481.7(14)	5180.5(13)	20.9(3)
C4	7577.1(14)	6368.0(14)	8566.0(13)	20.9(3)
C13	6270.4(15)	4598.2(15)	8785.3(14)	25.6(3)
C9	8824.8(14)	5018.5(15)	3748.1(13)	22.4(3)
C2	7373.7(14)	7661.8(14)	6291.1(13)	21.7(3)
C21	8232.6(15)	9842.5(15)	8778.2(14)	25.9(3)
C19	5915.7(16)	11302.8(15)	8946.3(14)	27.0(3)
C6	6018.5(14)	6506.9(15)	5656.7(14)	23.2(3)
C17	6158.1(15)	10050.7(15)	7648.6(14)	24.0(3)
C15	8578.7(15)	8495.5(15)	7450.5(15)	25.7(3)
C10	8677.6(15)	3659.5(15)	4215.5(15)	26.3(3)
C12	6827.3(15)	4133.5(15)	5639.3(14)	24.4(3)
C8	7981.5(14)	5930.6(14)	4232.7(13)	21.1(3)
C20	7376.3(16)	10750.3(16)	9260.2(15)	28.7(3)
C18	5304.4(15)	10958.9(15)	8133.5(14)	25.9(3)
C11	7687.1(15)	3223.3(15)	5155.1(15)	27.0(3)
C14	7944.5(16)	5960.7(16)	10004.9(14)	28.1(3)
C23	9932.5(16)	6778.7(17)	2243.5(16)	32.4(3)

Table S3. Anisotropic Displacement Parameters (Å^2×10^3) for **1c[PF$_6$]**. The Anisotropic displacement factor exponent takes the form: $-2\pi^2[h^2a^{*2}U_{11}+2hka^*b^*U_{12}+\ldots]$.

Atom	U_{11}	U_{22}	U_{33}	U_{23}	U_{13}	U_{12}
P1	24.02(18)	22.32(18)	19.80(18)	-11.78(14)	2.73(13)	-9.92(13)
F2	28.0(4)	43.6(5)	26.1(4)	-18.2(4)	7.4(3)	-17.7(4)
F4	34.5(5)	31.4(4)	26.9(4)	-13.0(4)	10.7(3)	-10.7(4)
F6	42.6(5)	24.4(4)	33.3(5)	-16.8(4)	3.9(4)	-10.1(4)
F3	27.7(4)	31.8(4)	27.3(4)	-12.3(4)	-1.3(3)	-14.3(3)
F7	48.8(5)	26.6(4)	30.8(4)	-16.6(4)	1.9(4)	-16.0(4)

Atom	U_{11}	U_{22}	U_{33}	U_{23}	U_{13}	U_{12}
F5	33.8(5)	43.7(5)	26.7(4)	-16.5(4)	-4.3(3)	-12.1(4)
O22	27.7(5)	35.4(5)	27.8(5)	-19.5(4)	7.0(4)	-12.6(4)
N3	25.1(6)	22.4(5)	22.1(5)	-14.4(5)	4.8(4)	-10.8(4)
N1	23.7(5)	20.6(5)	19.0(5)	-12.3(4)	2.5(4)	-7.8(4)
C5	22.8(6)	19.2(6)	18.0(6)	-10.3(5)	3.2(5)	-6.4(5)
C16	28.2(7)	21.2(6)	20.9(6)	-11.2(5)	5.8(5)	-13.7(5)
C7	22.3(6)	25.2(6)	19.5(6)	-13.4(5)	-1.4(5)	-7.4(5)
C4	24.3(6)	21.1(6)	19.0(6)	-11.0(5)	3.3(5)	-7.6(5)
C13	32.7(7)	24.3(7)	24.7(7)	-13.1(6)	5.5(5)	-14.0(6)
C9	22.2(6)	29.1(7)	20.9(6)	-15.0(6)	0.3(5)	-9.0(5)
C2	27.5(7)	20.9(6)	19.7(6)	-11.6(5)	4.3(5)	-8.8(5)
C21	27.9(7)	28.5(7)	27.7(7)	-15.5(6)	1.7(5)	-13.2(6)
C19	34.9(8)	25.3(7)	27.8(7)	-16.9(6)	9.1(6)	-13.4(6)
C6	24.5(6)	27.6(7)	22.8(6)	-15.9(6)	-0.3(5)	-7.8(5)
C17	29.1(7)	26.7(7)	23.2(6)	-13.9(6)	3.6(5)	-14.9(5)
C15	27.9(7)	28.8(7)	31.9(7)	-19.9(6)	7.8(6)	-15.9(6)
C10	28.4(7)	29.1(7)	30.2(7)	-21.4(6)	-0.1(6)	-7.6(6)
C12	27.8(7)	29.4(7)	22.9(6)	-14.5(6)	2.2(5)	-14.4(6)
C8	24.4(6)	22.6(6)	21.1(6)	-12.3(5)	-0.9(5)	-9.3(5)
C20	38.6(8)	31.7(7)	27.8(7)	-19.6(6)	3.4(6)	-17.4(6)
C18	26.9(7)	25.6(7)	26.8(7)	-12.6(6)	4.2(5)	-11.0(5)
C11	33.9(7)	24.7(7)	30.0(7)	-16.4(6)	-0.5(6)	-12.5(6)
C14	34.8(8)	31.3(7)	21.6(7)	-14.4(6)	0.3(5)	-11.6(6)
C23	31.5(8)	36.0(8)	30.0(8)	-14.3(7)	6.3(6)	-15.0(6)

Table S4. Bond Lengths for **1c[PF$_6$]**.

Atom	Atom	Length/Å	Atom	Atom	Length/Å
P1	F2	1.6018(8)	C16	C21	1.3952(18)
P1	F4	1.6068(8)	C16	C17	1.3905(19)
P1	F6	1.6079(8)	C16	C15	1.5145(18)
P1	F3	1.5956(8)	C7	C6	1.5137(18)
P1	F7	1.6015(8)	C7	C12	1.3856(18)
P1	F5	1.6037(8)	C7	C8	1.3993(18)
O22	C9	1.3697(16)	C4	C14	1.4888(18)
O22	C23	1.4265(18)	C9	C10	1.3951(19)
N3	C4	1.3909(17)	C9	C8	1.3900(18)
N3	C2	1.3300(17)	C21	C20	1.389(2)
N3	C15	1.4725(16)	C19	C20	1.383(2)
N1	C5	1.3891(16)	C19	C18	1.389(2)
N1	C2	1.3257(16)	C17	C18	1.3905(19)
N1	C6	1.4752(16)	C10	C11	1.380(2)
C5	C4	1.3638(18)	C12	C11	1.3936(19)
C5	C13	1.4848(18)			

Table S5. Bond Angles for **1c[PF$_6$]**.

Atom	Atom	Atom	Angle/°	Atom	Atom	Atom	Angle/°
F2	P1	F4	179.73(4)	C21	C16	C15	119.41(12)
F2	P1	F6	90.39(5)	C17	C16	C21	119.18(12)
F2	P1	F5	90.20(5)	C17	C16	C15	121.39(12)
F4	P1	F6	89.59(5)	C12	C7	C6	120.64(12)
F3	P1	F2	89.99(4)	C12	C7	C8	120.19(12)
F3	P1	F4	90.28(5)	C8	C7	C6	119.13(12)
F3	P1	F6	90.15(5)	N3	C4	C14	122.74(12)
F3	P1	F7	89.99(5)	C5	C4	N3	106.36(11)
F3	P1	F5	179.70(5)	C5	C4	C14	130.84(12)
F7	P1	F2	89.87(5)	O22	C9	C10	115.59(12)
F7	P1	F4	90.15(5)	O22	C9	C8	124.24(12)
F7	P1	F6	179.71(5)	C8	C9	C10	120.16(13)
F7	P1	F5	90.25(5)	N1	C2	N3	108.59(11)
F5	P1	F4	89.53(5)	C20	C21	C16	120.23(13)
F5	P1	F6	89.61(5)	C20	C19	C18	119.80(13)
C9	O22	C23	116.83(11)	N1	C6	C7	113.39(11)
C4	N3	C15	126.52(11)	C16	C17	C18	120.40(12)
C2	N3	C4	109.16(11)	N3	C15	C16	112.01(11)
C2	N3	C15	124.22(11)	C11	C10	C9	119.91(13)
C5	N1	C6	126.64(11)	C7	C12	C11	119.77(13)
C2	N1	C5	109.12(10)	C9	C8	C7	119.52(12)
C2	N1	C6	124.19(11)	C19	C20	C21	120.34(12)
N1	C5	C13	122.26(11)	C19	C18	C17	120.04(13)
C4	C5	N1	106.77(11)	C10	C11	C12	120.43(13)
C4	C5	C13	130.96(12)				

Table S6. Torsion Angles for **1c[PF$_6$]**.

A	B	C	D	Angle/°	A	B	C	D	Angle/°
O22	C9	C10	C11	-178.37(12)	C6	N1	C5	C4	-177.01(12)
O22	C9	C8	C7	177.94(11)	C6	N1	C5	C13	1.83(19)
N1	C5	C4	N3	-0.62(14)	C6	N1	C2	N3	177.46(11)
N1	C5	C4	C14	176.81(13)	C6	C7	C12	C11	177.96(12)
C5	N1	C2	N3	-0.07(15)	C6	C7	C8	C9	-177.45(11)
C5	N1	C6	C7	-89.91(15)	C17	C16	C21	C20	0.7(2)
C16	C21	C20	C19	0.0(2)	C17	C16	C15	N3	-37.36(18)
C16	C17	C18	C19	0.1(2)	C15	N3	C4	C5	176.99(12)
C7	C12	C11	C10	-0.4(2)	C15	N3	C4	C14	-0.7(2)
C4	N3	C2	N1	-0.33(15)	C15	N3	C2	N1	-176.82(11)
C4	N3	C15	C16	-69.54(17)	C15	C16	C21	C20	179.62(13)
C13	C5	C4	N3	-179.33(13)	C15	C16	C17	C18	-179.62(12)
C13	C5	C4	C14	-1.9(2)	C10	C9	C8	C7	-0.68(19)

A	B	C	D	Angle/°	A	B	C	D	Angle/°
C9	C10	C11	C12	0.2(2)	C12	C7	C6	N1	107.05(14)
C2	N3	C4	C5	0.60(15)	C12	C7	C8	C9	0.45(18)
C2	N3	C4	C14	-177.10(12)	C8	C7	C6	N1	-75.06(15)
C2	N3	C15	C16	106.33(14)	C8	C7	C12	C11	0.09(19)
C2	N1	C5	C4	0.45(14)	C8	C9	C10	C11	0.4(2)
C2	N1	C5	C13	179.29(12)	C20	C19	C18	C17	0.6(2)
C2	N1	C6	C7	92.99(15)	C18	C19	C20	C21	-0.6(2)
C21	C16	C17	C18	-0.8(2)	C23	O22	C9	C10	176.99(12)
C21	C16	C15	N3	143.79(12)	C23	O22	C9	C8	-1.68(18)

Table S7. Hydrogen Atom Coordinates (Å×10^4) and Isotropic Displacement Parameters (Å^2×10^3) for **1c[PF$_6$]**.

Atom	*x*	*y*	*z*	U(eq)
H13A	5230.68	4966.02	8596.29	38
H13B	6498.26	4128.01	9777.58	38
H13C	6684.55	3896.86	8457.03	38
H2	7439.93	8370.75	5378.66	26
H21	9235.3	9467.47	8997.23	31
H19	5332.19	11915.7	9285.44	32
H6A	5632.04	7471.14	4845.47	28
H6B	5200.79	6159.07	6091.33	28
H17	5736.3	9819.11	7092.01	29
H15A	8828.9	9090.49	6509.44	31
H15B	9475.96	7915.4	8047.05	31
H10	9258.79	3035.39	3887.22	32
H12	6142.37	3830.62	6281.72	29
H8	8087.87	6852.01	3922.75	25
H20	7796.12	10992.9	9808.27	34
H18	4302.5	11344.48	7908.85	31
H11	7590.48	2296.41	5474.19	32
H14A	8949.2	5876.44	10102.55	42
H14B	7790.83	5032.64	10637.22	42
H14C	7335.25	6704.91	10221.98	42
H23A	10613.46	6929.55	1525.43	49
H23B	10272.26	6873.53	2979.56	49
H23C	8996.34	7502.21	1849.89	49

Crystal structure determination of compound 1c[PF6]

Crystal Data for $C_{20}H_{23}F_6N_2OP$ (M =452.37 g/mol): triclinic, space group P-1 (no. 2), a = 9.8930(3) Å, b = 11.1547(4) Å, c = 11.1640(4) Å, α = 60.849(4)°, β = 84.409(3)°, γ = 71.453(3)°, V = 1017.60(7) Å^3, Z = 2, T = 100.00(10) K, μ(Cu Kα) = 1.834 mm^{-1}, $Dcalc$ = 1.476 g/cm^3, 23177 reflections measured (9.092° ≤ 2Θ ≤ 157.666°), 4125 unique (R_{int} = 0.0370, R_{sigma} = 0.0187) which

were used in all calculations. The final R_1 was 0.0319 (I > 2σ(I)) and wR_2 was 0.0824 (all data).

Refinement model description

Number of restraints - 0, number of constraints - unknown.

Details:

```
1. Fixed Uiso
 At 1.2 times of:
  All C(H) groups, All C(H,H) groups
 At 1.5 times of:
  All C(H,H,H) groups
2.a Secondary CH2 refined with riding coordinates:
 C6(H6A,H6B), C15(H15A,H15B)
2.b Aromatic/amide H refined with riding coordinates:
 C2(H2), C21(H21), C19(H19), C17(H17), C10(H10), C12(H12), C8(H8), C20(H20),
 C18(H18), C11(H11)
2.c Idealised Me refined as rotating group:
 C13(H13A,H13B,H13C), C14(H14A,H14B,H14C), C23(H23A,H23B,H23C)
```

This report has been created with Olex2, compiled on 2020.11.27 svn.r5f609507 for Rigaku Oxford Diffraction. Please let us know if there are any errors or if you would like to have additional features.

References

[1] Dolomanov, O.V., Bourhis, L.J., Gildea, R.J, Howard, J.A.K. & Puschmann, H. (2009), J. Appl. Cryst. 42, 339-341.

[2] Sheldrick, G. M. (2015). Acta Cryst. C71, 3-8. doi:10.1107/s2053229614024218

[3] Sheldrick, G. M. (2008). Acta Cryst. A64, 112-122. doi:10.1107/S0108767307043930
