# Supplementary figures and images for: Synthesis and Cytotoxic Activity of Lepidilines A–D: Comparison with Some 4,5-Diphenyl Analogues and Related Imidazole-2-thiones
Source: J Nat Prod. 2021 Nov 22;84(12):3071–9. doi: 10.1021/acs.jnatprod.1c00797 (PMC8713287; doi:10.1021/acs.jnatprod.1c00797)

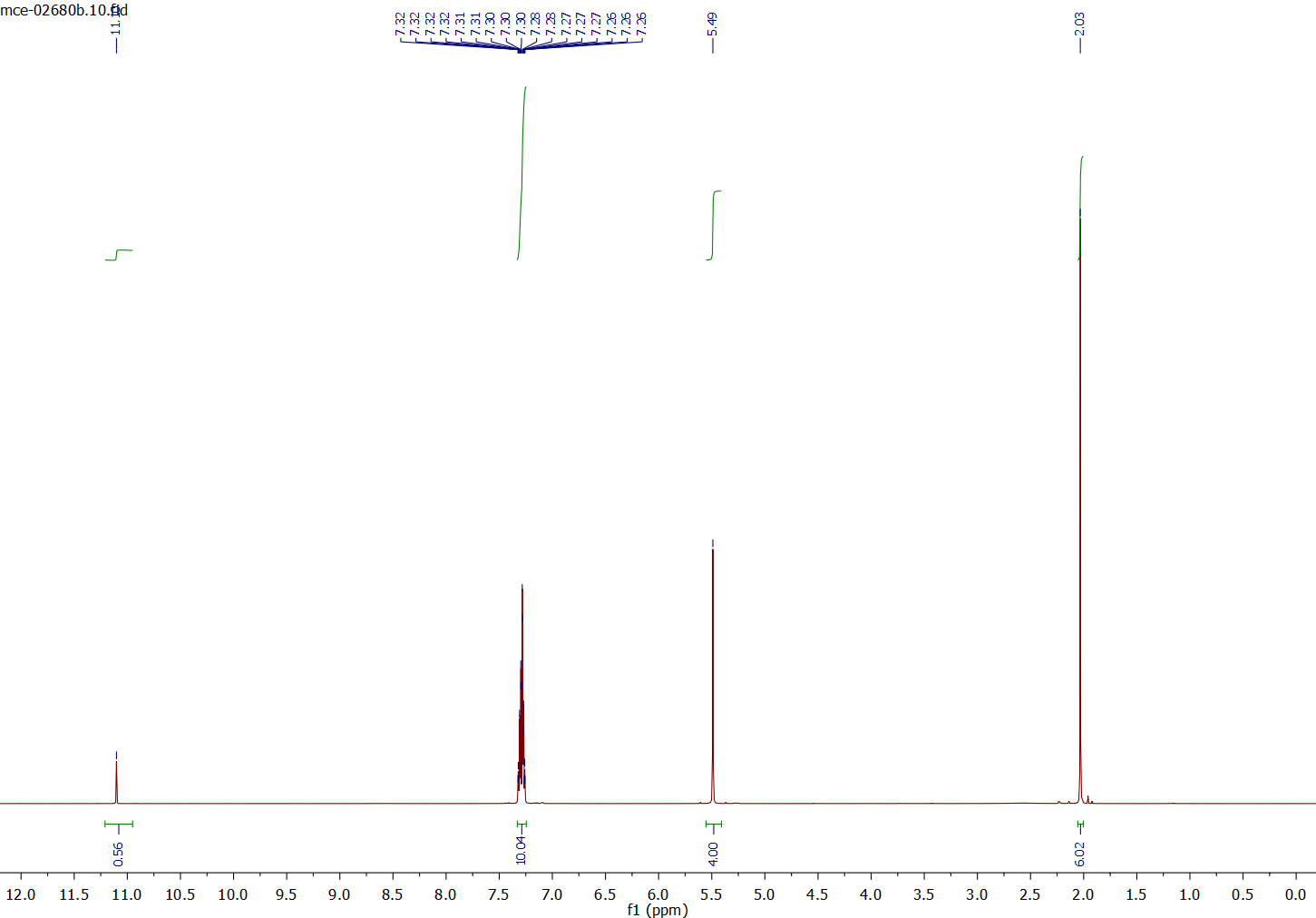

Supplement: Supplementary file 3 — np1c00797_si_003.zip [file np1c00797_si_003.zip › NMR data/1a (lepidiline A)/mce-02680b/10/1a 1hnmr.png]

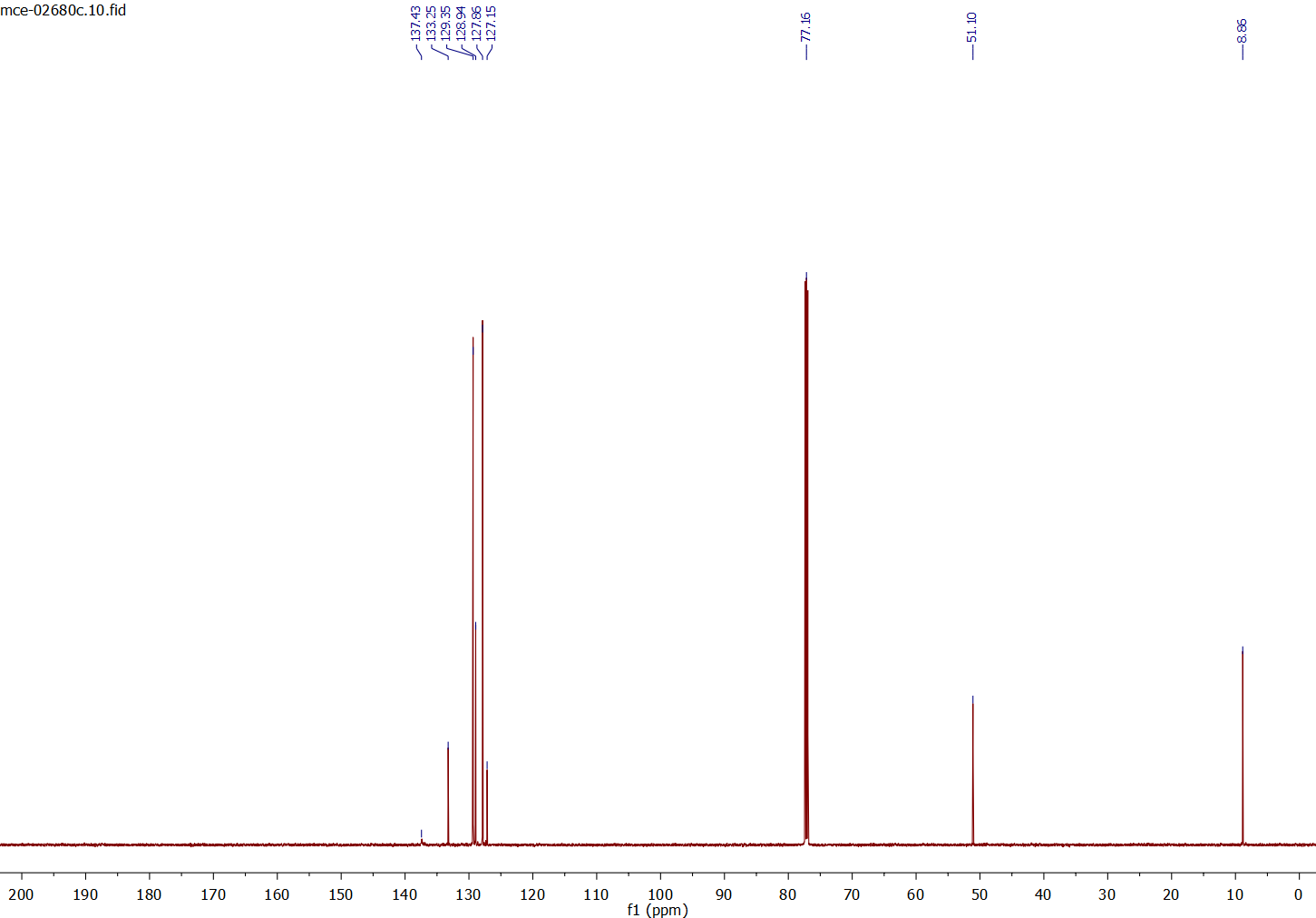

Supplement: Supplementary file 3 — np1c00797_si_003.zip [file np1c00797_si_003.zip › NMR data/1a (lepidiline A)/mce-02680c/10/1a 13CNMR.png]

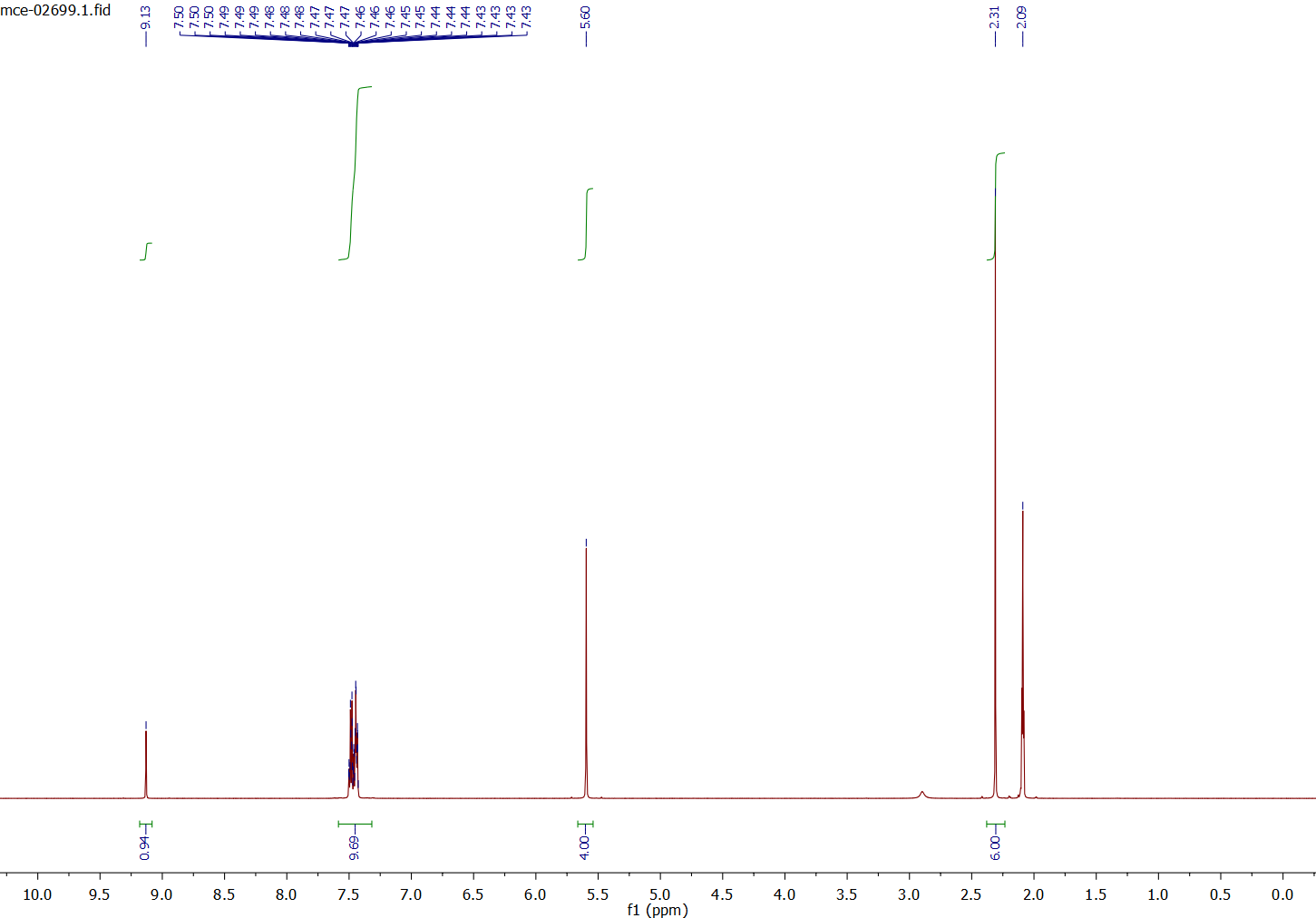

Supplement: Supplementary file 3 — np1c00797_si_003.zip [file np1c00797_si_003.zip › NMR data/1a[PF6]/mce-02699/1/1a[PF6] 1HNMR.png]

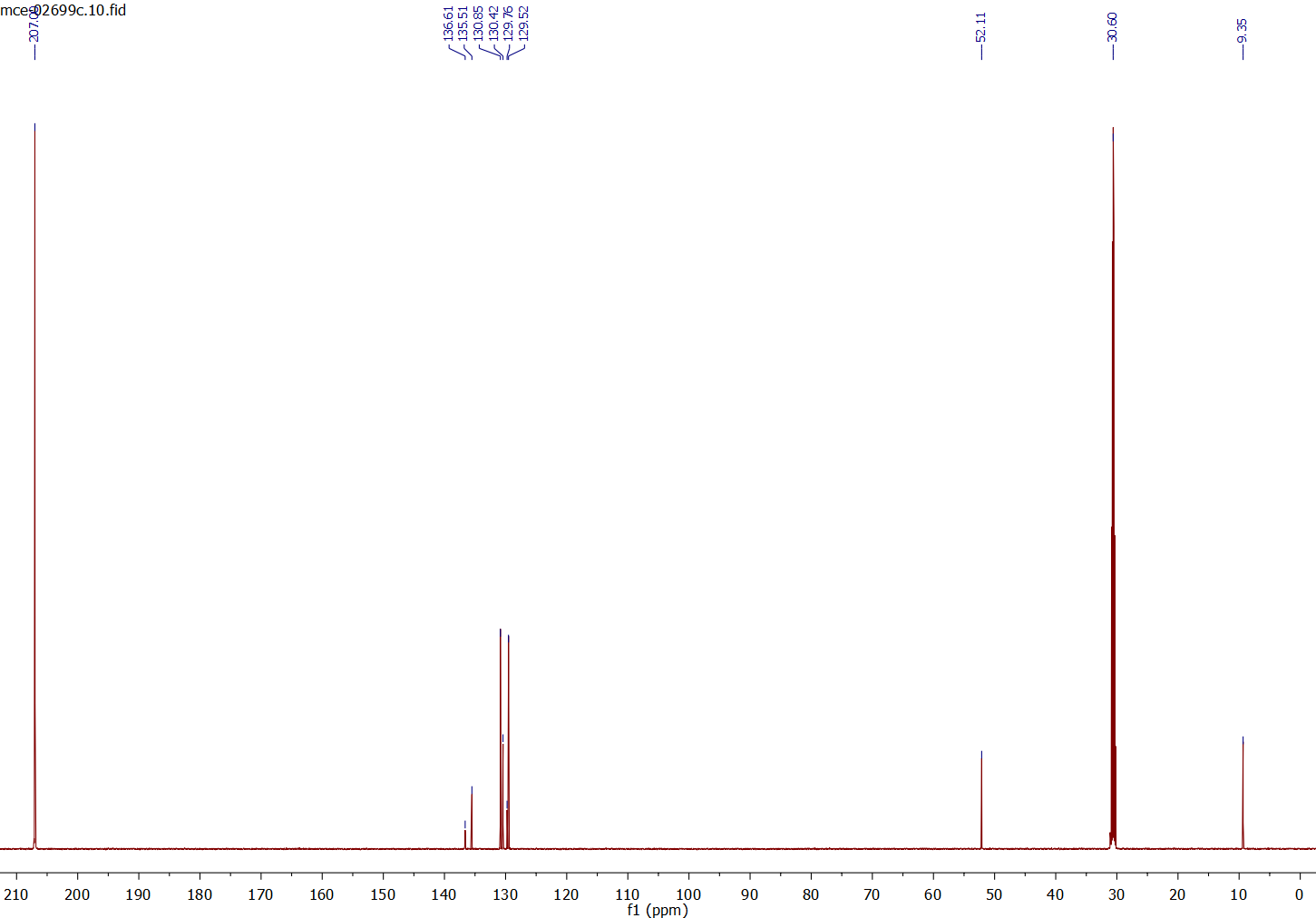

Supplement: Supplementary file 3 — np1c00797_si_003.zip [file np1c00797_si_003.zip › NMR data/1a[PF6]/mce-02699c/10/1a[PF6] 13CNMR.png]

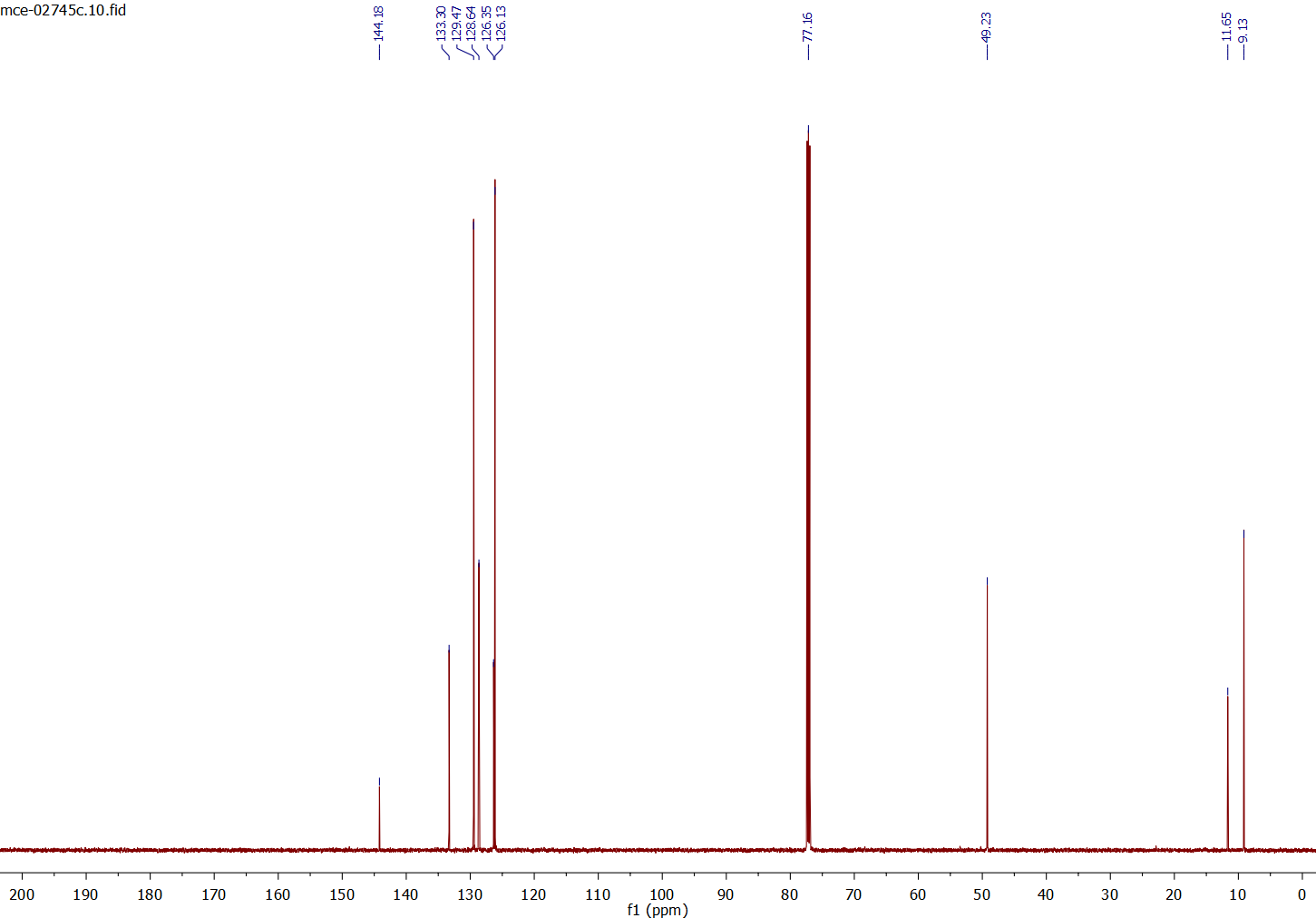

Supplement: Supplementary file 3 — np1c00797_si_003.zip [file np1c00797_si_003.zip › NMR data/1b (lepidiline B)/mce-02745c/10/1b 13CNMR.png]

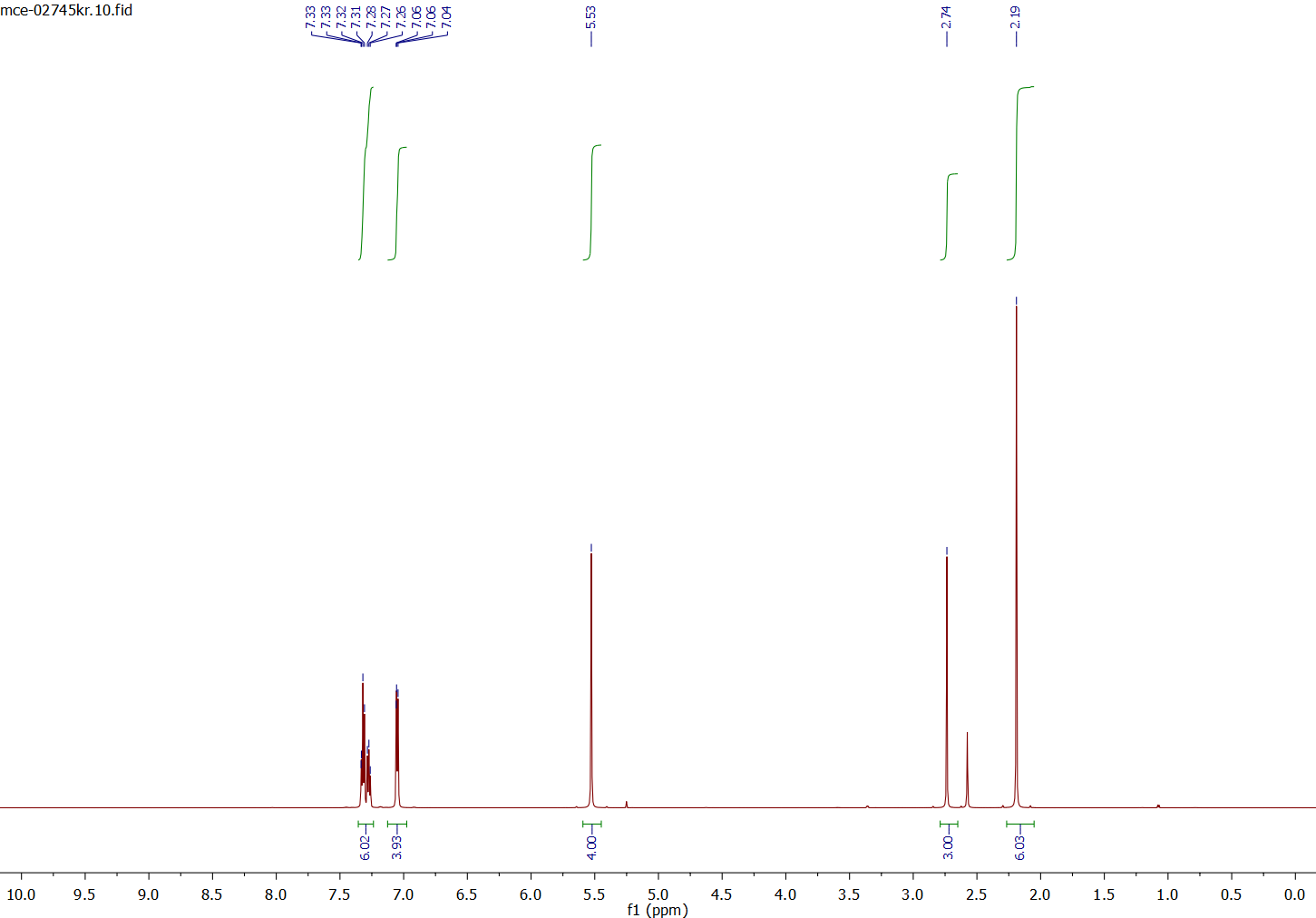

Supplement: Supplementary file 3 — np1c00797_si_003.zip [file np1c00797_si_003.zip › NMR data/1b (lepidiline B)/mce-02745kr/10/1b 1hnmr.png]

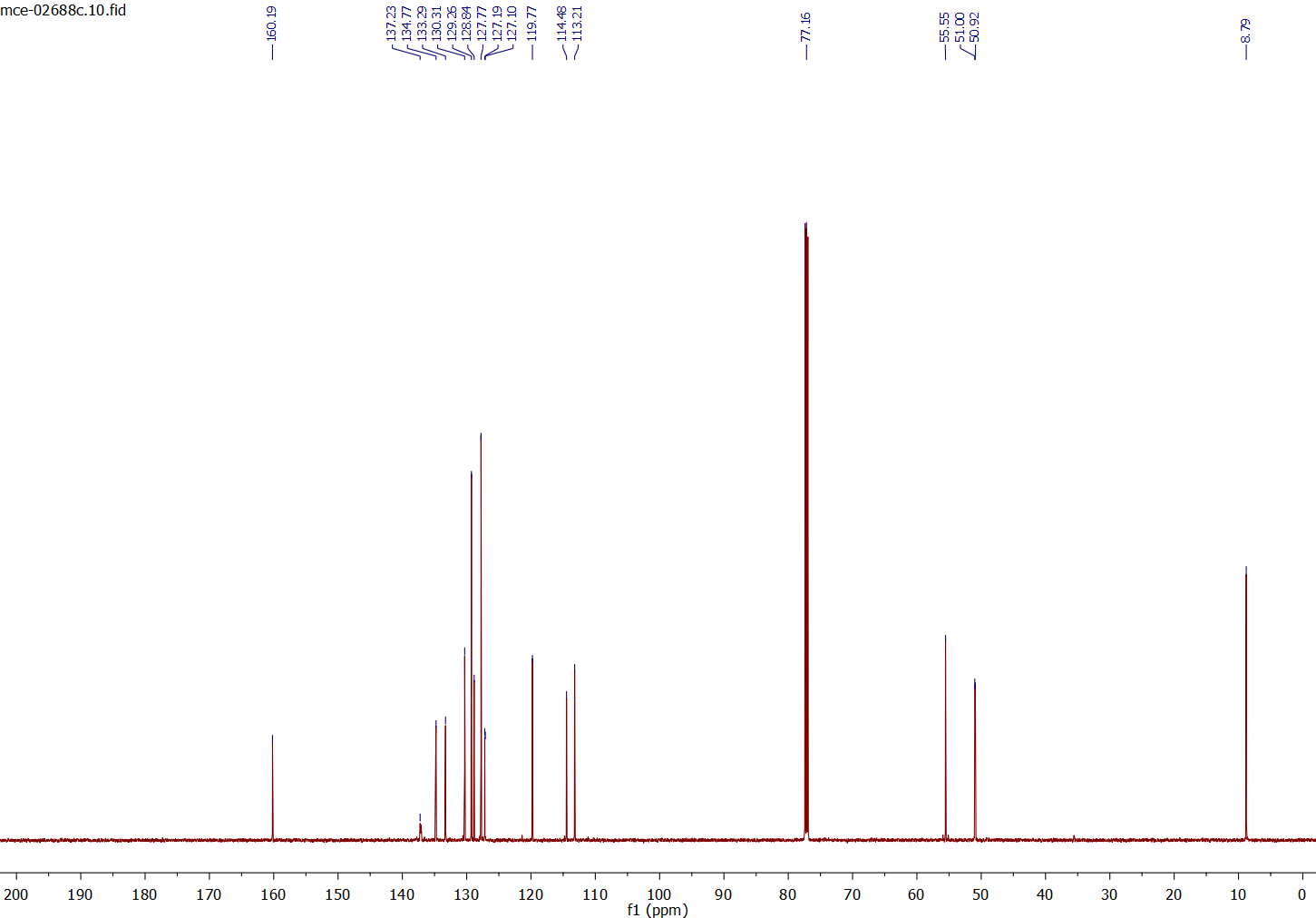

Supplement: Supplementary file 3 — np1c00797_si_003.zip [file np1c00797_si_003.zip › NMR data/1c (lepidiline C)/mce-02688c/10/1c 13nmr.png]

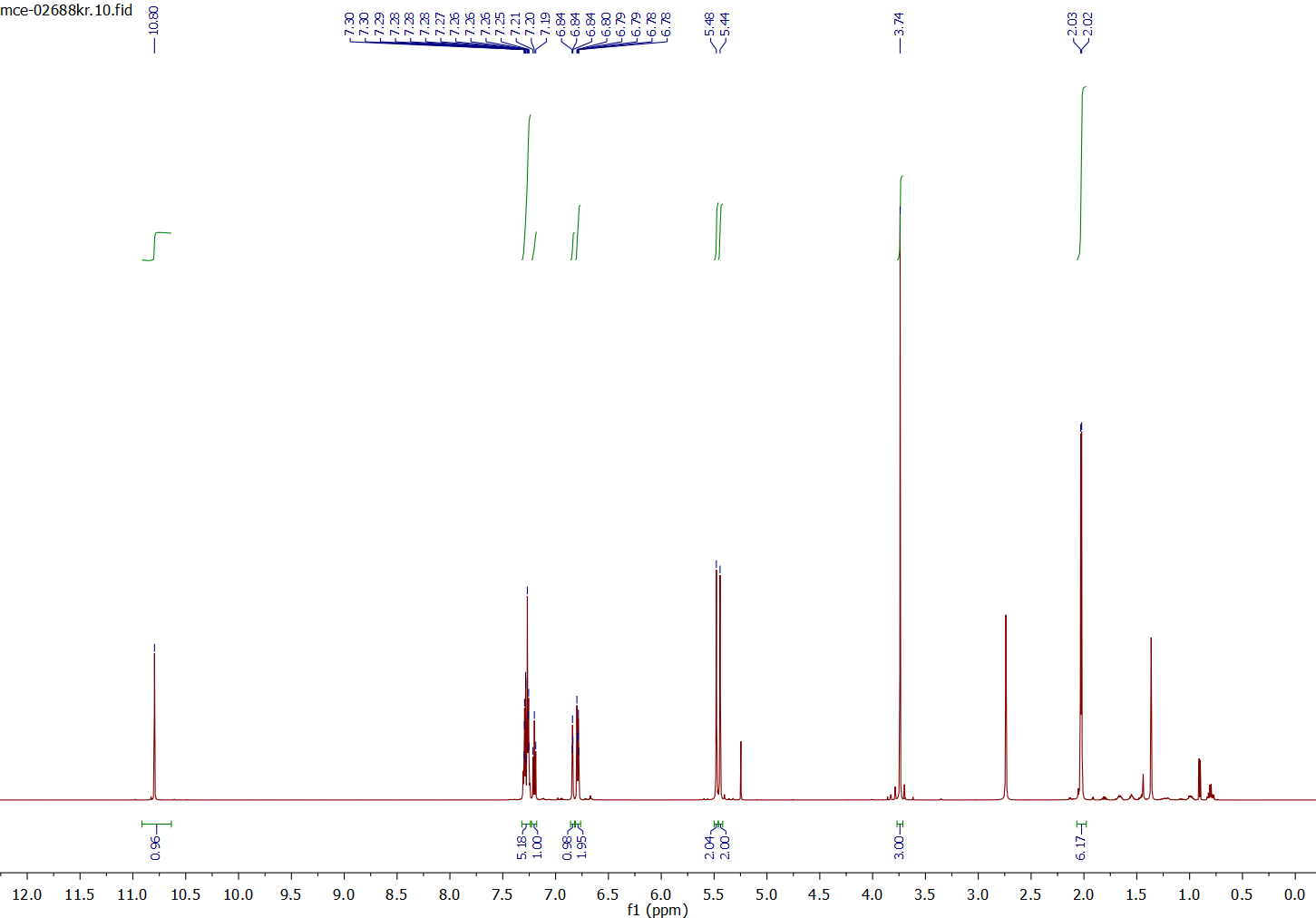

Supplement: Supplementary file 3 — np1c00797_si_003.zip [file np1c00797_si_003.zip › NMR data/1c (lepidiline C)/mce-02688kr/10/1c 1hNMR.png]

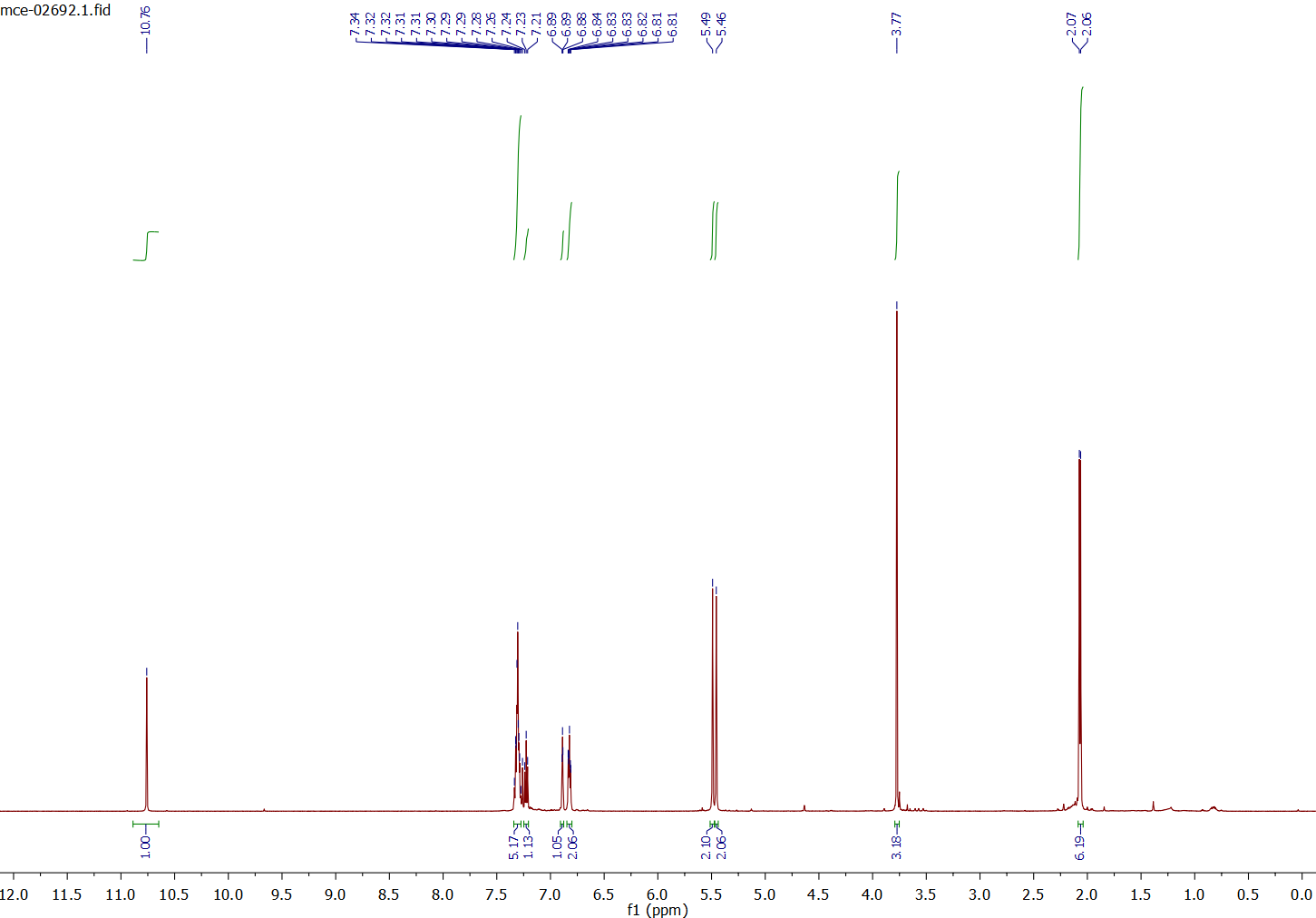

Supplement: Supplementary file 3 — np1c00797_si_003.zip [file np1c00797_si_003.zip › NMR data/1c[Br]/mce-02692/1/1c[Br] 1hnmr.png]

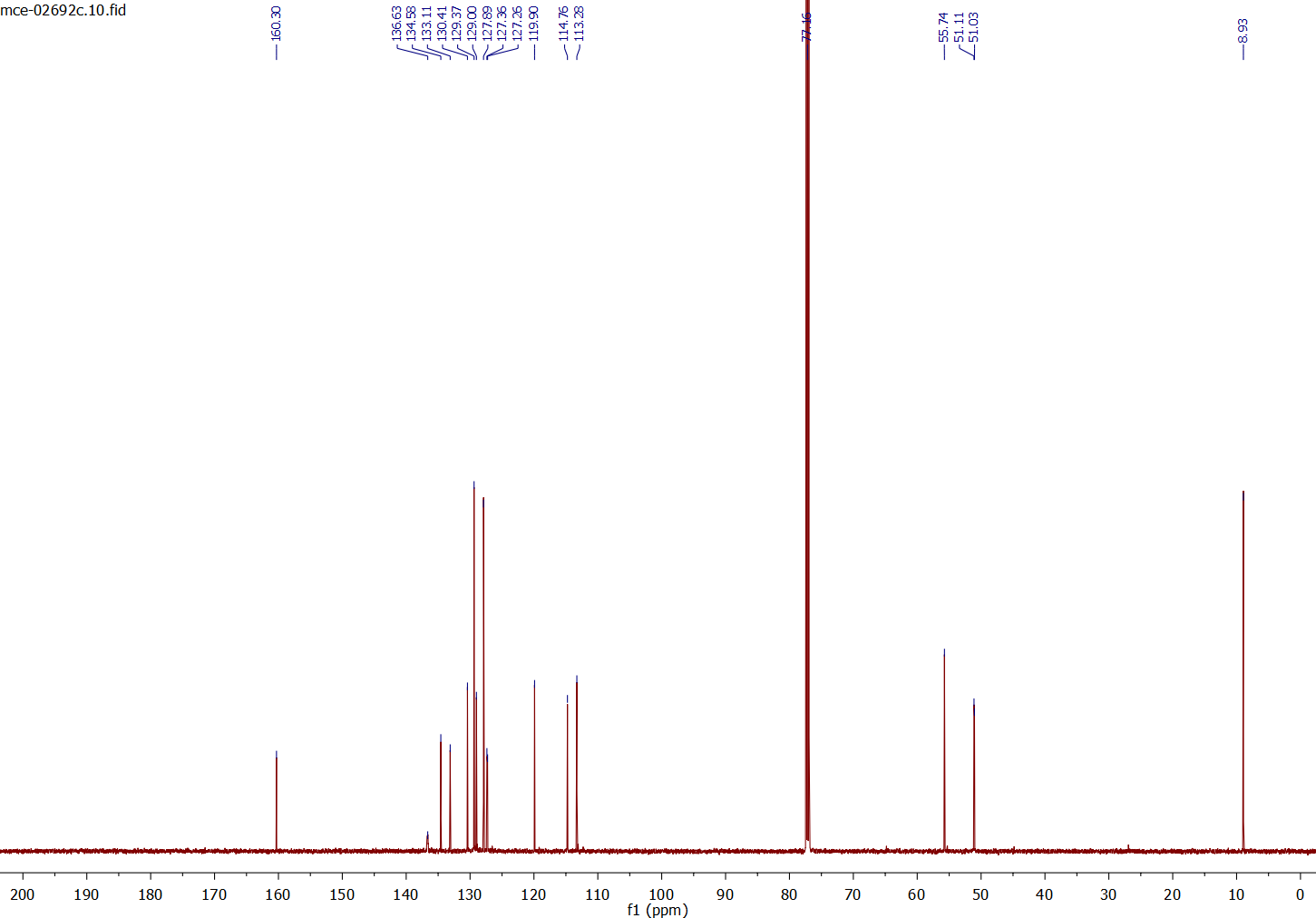

Supplement: Supplementary file 3 — np1c00797_si_003.zip [file np1c00797_si_003.zip › NMR data/1c[Br]/mce-02692c/10/1c[Br] 13CNMR.png]

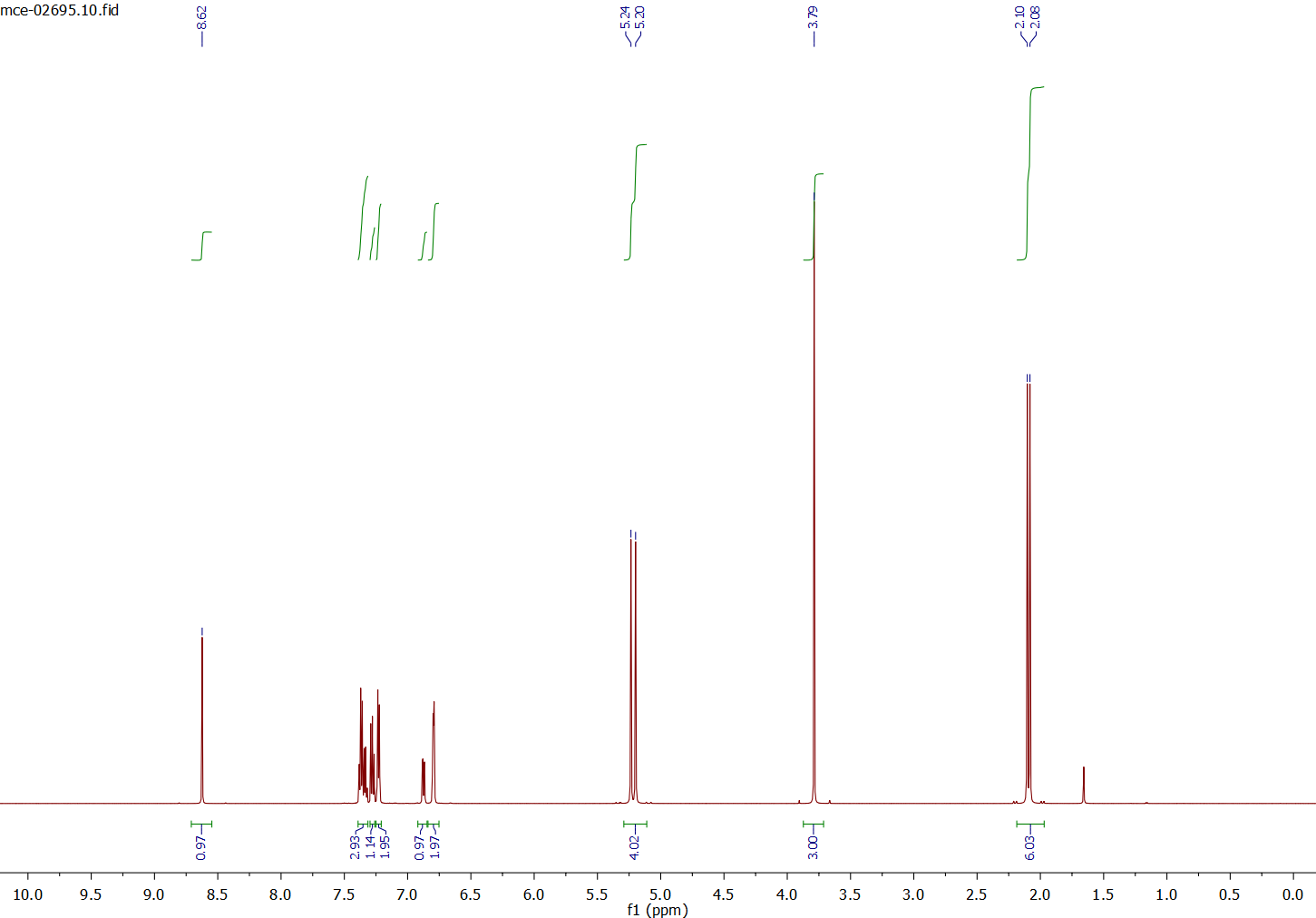

Supplement: Supplementary file 3 — np1c00797_si_003.zip [file np1c00797_si_003.zip › NMR data/1c[PF6]/mce-02695/10/1c[PF6] 1HNMR.png]

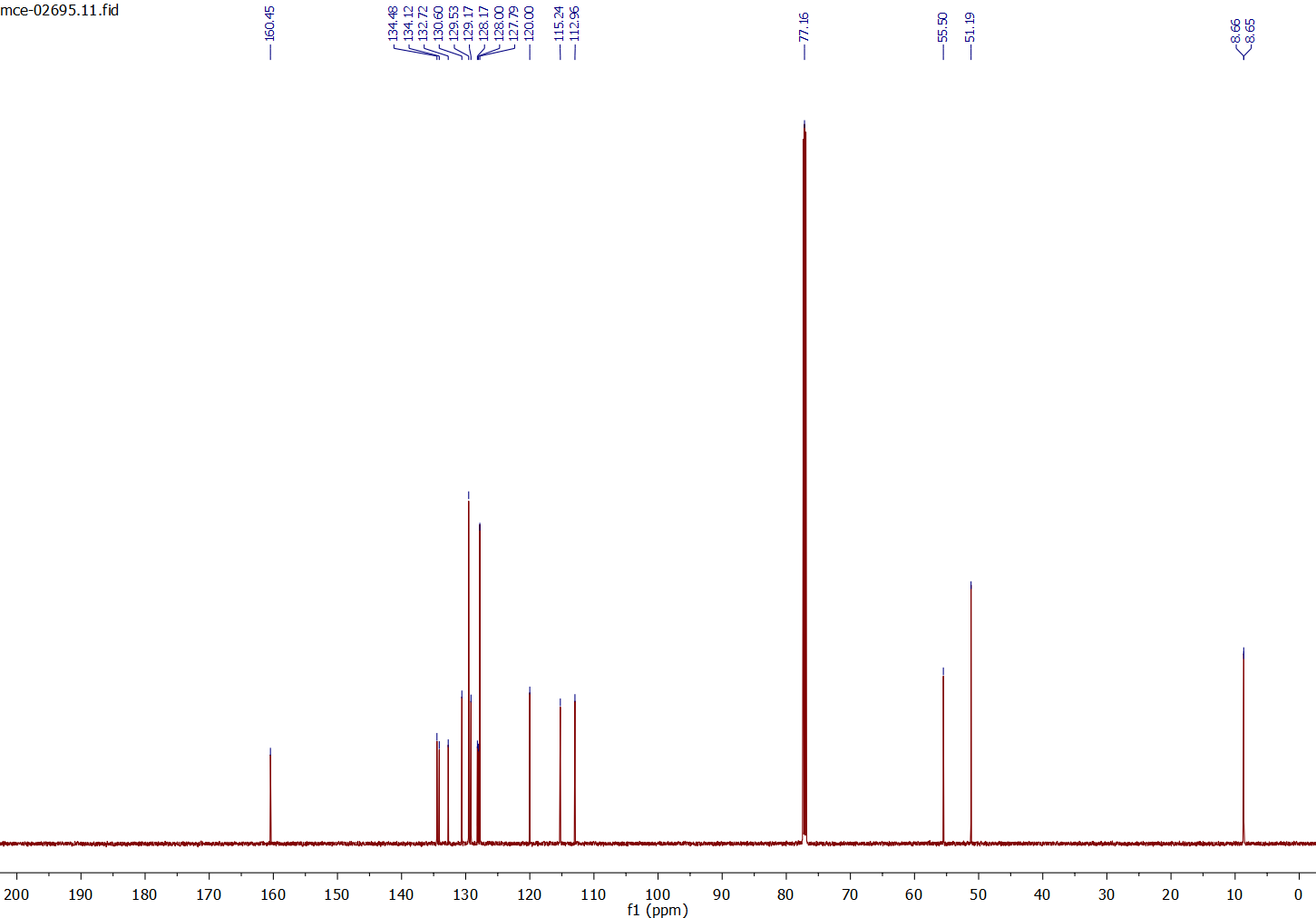

Supplement: Supplementary file 3 — np1c00797_si_003.zip [file np1c00797_si_003.zip › NMR data/1c[PF6]/mce-02695/11/1c[PF6] 13CNMR.png]

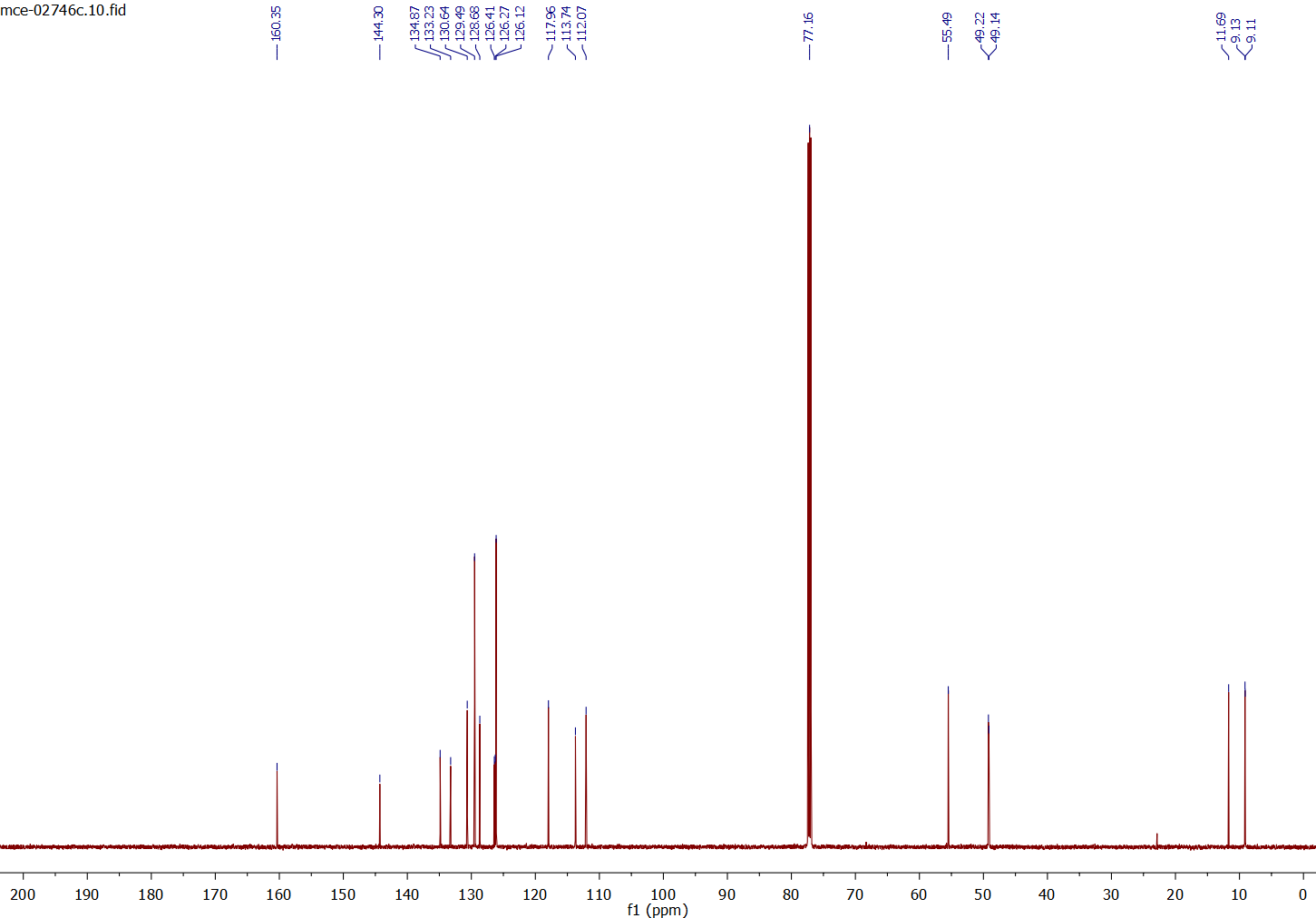

Supplement: Supplementary file 3 — np1c00797_si_003.zip [file np1c00797_si_003.zip › NMR data/1d (lepidiline D)/mce-02746c/10/1d 13CNMR.png]

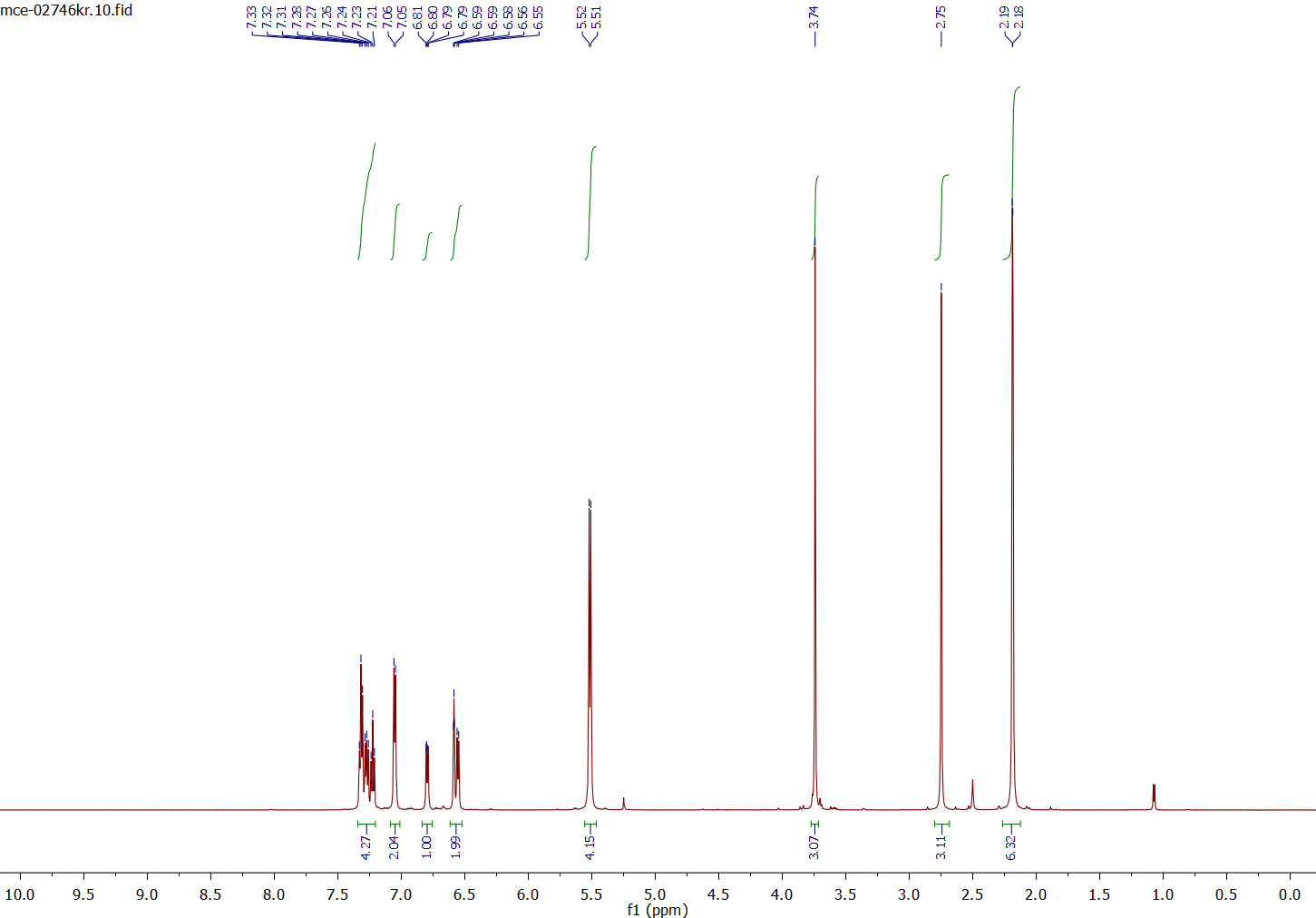

Supplement: Supplementary file 3 — np1c00797_si_003.zip [file np1c00797_si_003.zip › NMR data/1d (lepidiline D)/mce-02746kr/10/1d 1hnmr.png]

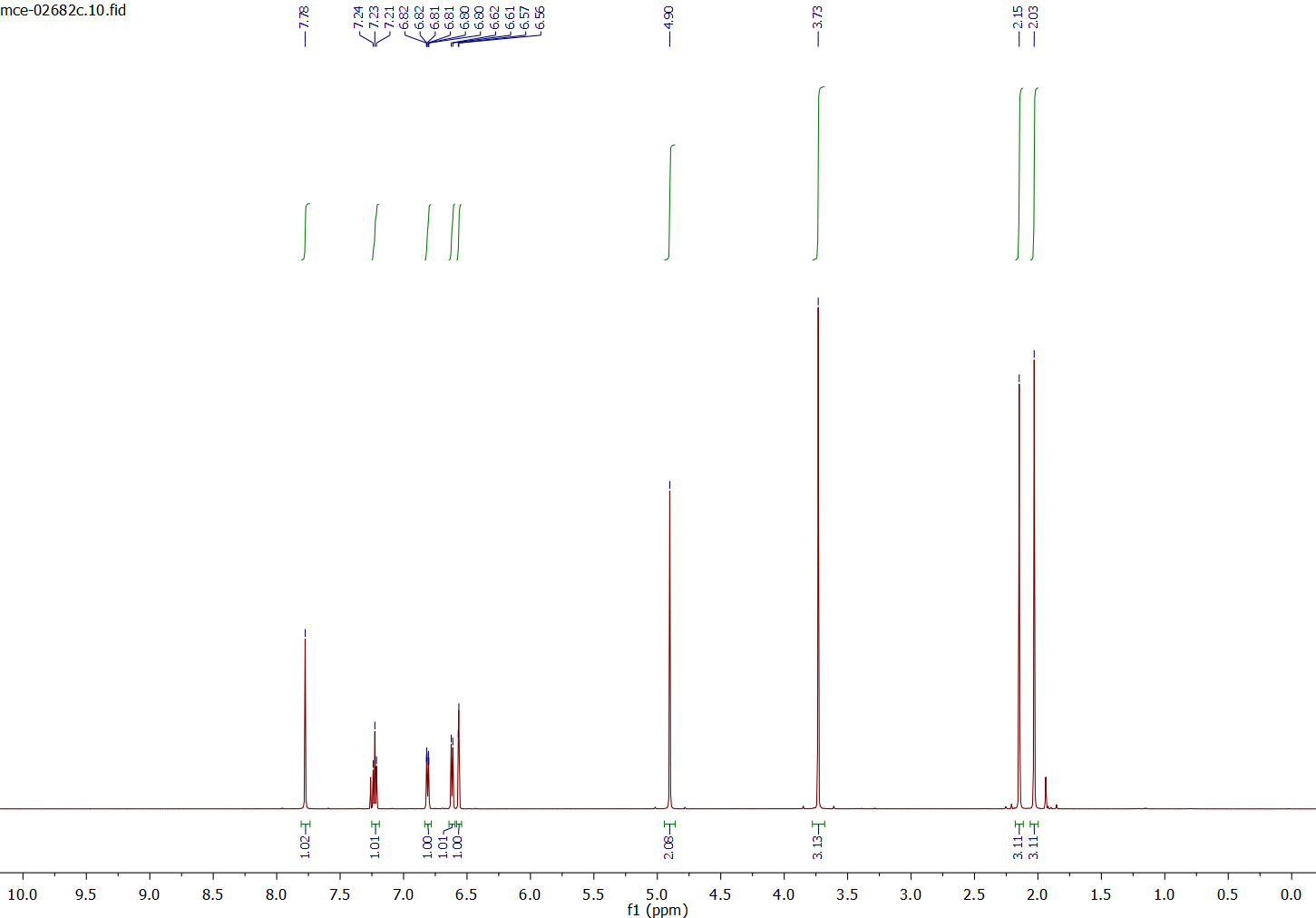

Supplement: Supplementary file 3 — np1c00797_si_003.zip [file np1c00797_si_003.zip › NMR data/2c/mce-02682c/10/2c 1hnmr.png]
